# Effects of School-Based Educational Interventions for Enhancing Adolescents Abilities in Critical Appraisal of Health Claims: A Systematic Review

**DOI:** 10.1371/journal.pone.0161485

**Published:** 2016-08-24

**Authors:** Lena V. Nordheim, Malene W. Gundersen, Birgitte Espehaug, Øystein Guttersrud, Signe Flottorp

**Affiliations:** 1 Faculty of Health and Social Sciences, Centre for Evidence Based Practice, Bergen University College, Bergen, Norway; 2 Faculty of Medicine and Dentistry, Department of Global Public Health and Primary Care, University of Bergen, Bergen, Norway; 3 Oslo and Akershus University College of Applied Sciences, Oslo, Norway; 4 Norwegian Centre for Science Education, University of Oslo, Oslo, Norway; 5 Department for Evidence Synthesis, Norwegian Institute of Public Health, Oslo, Norway; 6 Institute of Health and Society, University of Oslo, Oslo, Norway; Peking University First Hospital, CHINA

## Abstract

**Background and Objective:**

Adolescents are frequent media users who access health claims from various sources. The plethora of conflicting, pseudo-scientific, and often misleading health claims in popular media makes critical appraisal of health claims an essential ability. Schools play an important role in educating youth to critically appraise health claims. The objective of this systematic review was to evaluate the effects of school-based educational interventions for enhancing adolescents’ abilities in critically appraising health claims.

**Methods:**

We searched MEDLINE, Embase, PsycINFO, AMED, Cinahl, Teachers Reference Centre, LISTA, ERIC, Sociological Abstracts, Social Services Abstracts, The Cochrane Library, Science Citation Index Expanded, Social Sciences Citation Index, and sources of grey literature. Studies that evaluated school-based educational interventions to improve adolescents’ critical appraisal ability for health claims through advancing the students’ knowledge about science were included. Eligible study designs were randomised and non-randomised controlled trials, and interrupted time series. Two authors independently selected studies, extracted data, and assessed risk of bias in included studies. Due to heterogeneity in interventions and inadequate reporting of results, we performed a descriptive synthesis of studies. We used GRADE (Grading of Recommendations, Assessment, Development, and Evaluation) to assess the certainty of the evidence.

**Results:**

Eight studies were included: two compared different teaching modalities, while the others compared educational interventions to instruction as usual. Studies mostly reported positive short-term effects on critical appraisal-related knowledge and skills in favour of the educational interventions. However, the certainty of the evidence for all comparisons and outcomes was very low.

**Conclusion:**

Educational interventions in schools may have beneficial short-term effects on knowledge and skills relevant to the critical appraisal of health claims. The small number of studies, their heterogeneity, and the predominantly high risk of bias inhibit any firm conclusions about their effects. None of the studies evaluated any long-term effects of interventions. Future intervention studies should adhere to high methodological standards, target a wider variety of school-based settings, and include a process evaluation.

**Systematic Review Registration:**

PROSPERO no. CRD42015017936.

## Introduction

The multitude of channels distributing health information and products that claim to cure everything from acne to various forms of cancer place demands on children’s and adolescents’ health literacy [1]. The average time youth aged 8 to18 spent using any kind of media increased from 6 hours and 19 minutes to 7 hours and 38 minutes between 1999 and 2009 [2]. Whether purposeful or not, adolescents may encounter health claims through various media, including the Internet, social media, television, and magazines [3–5].

A health claim typically suggest that a causal factor (a medical treatment, a diet, a hazard) increases or reduces the chance of a certain outcome. Even if claims appear to be scientifically sound, they are often based on preliminary or poorly designed and executed studies, pseudo-scientific facts, or inflated expert opinions [6, 7]. Health claims in the media might influence peoples’ actions and behaviour [8–10]. Relying on misleading and unsubstantiated claims may thus adversely affect individual health and lead to unnecessary use of health care resources. Several studies have reported that adolescents lack abilities in judging the trustworthiness and scientific soundness of claims [5, 11, 12], and this deficiency continues during higher education and adulthood [13]. Critical appraisal skills are crucial to enable adolescents to distinguish reliable from unreliable claims. Schools are essential for fostering these skills, given their relevance for students' present and future lives [1, 14].

The term *critical appraisal* is often used to describe the evaluation of the validity of scientific papers for application in health care settings; however, it could equally apply to evaluating health claims in contemporary media [15]. For both health professionals and laypersons, knowledge about the strengths and limitations of methods used to produce scientific knowledge is important to critically appraise health claims. Ryder [16] analysed case studies on public understanding of science, the majority of them health-related. He concluded that scientific content knowledge (e.g. understanding how the human body digests and absorbs carbohydrates) was important, but not as central to decision-making as was knowledge *about* science. Accordingly, he suggested a framework of learning aims for school science that encompasses knowledge about science; including knowledge about the methods scientists use to obtain valid and precise data, uncertainty in science, and issues of science communication in the media and elsewhere [17]. Critical appraisal therefore involves using knowledge about science to decide whether health claims in contemporary media and elsewhere in society can be trusted. This in turn will help in handling the problem of information overload ([15], p. 4). Instruction in critical appraisal of health claims is relevant to school subjects such as science, mathematics, health and physical education, and can take various forms and contents. Relevant teaching topics include epidemiology and aspects related to evidence-based health care, including the principles of causal reasoning (e.g. how to distinguish causation from correlation), recognising the need for fair comparisons of treatments, and understanding probabilities and risks [18–21].

The terms critical thinking and critical appraisal are sometimes used interchangeably. Both are disciplines concerned with how claims are developed and justified. Critical thinking may or may not involve evaluating the scientific validity of claims, and it is therefore a broader concept than critical appraisal [22]. A recent review and meta-analysis identified many studies on the effects of teaching critical thinking in primary, secondary and higher education. Constructivist-teaching approaches such as teacher-led discussions, authentic problem solving, and mentorship, were particularly effective in promoting critical thinking regardless of educational level [23]. It was not possible to derive from the review whether these teaching approaches improved students’ understanding of science and critical appraisal abilities specifically, and the review did not address health claims as such. Likewise, the topic of health claims was absent in two other reviews of school-based interventions that aimed to increase students’ understanding of science in contexts relevant to everyday life; and there was insufficient evidence to support or refute any specific teaching method [24, 25].

Critical appraisal skills are important to a person’s overall health literacy [26]. In Nutbeam’s health literacy framework [27], abilities in critical appraisal reflect the category of “critical health literacy”, i.e. the more advanced cognitive abilities required to critically analyse and use health information (and claims herein) to improve health and well-being. Interventions to improve health literacy have mostly emphasised “functional health literacy”, a term Nutbeam uses to describe basic literacy and numeracy skills to understand information about how to use medications and health care services, as well as knowledge of health conditions [27]. For instance, a systematic review showed mixed results for strategies to enhance understanding of scientific information, such as risks and benefits of treatments, among individuals with low health literacy. However, the included studies emphasised comprehension rather than critical appraisal, and only involved adults in clinical settings [28]. The few systematic reviews that address critical health literacy as an outcome have found weak and inconclusive evidence as to which interventions are effective [29, 30]. The interventions mainly aimed at teaching people how to evaluate the authority behind claims, such as authors’ credentials and motivations, rather than their scientific soundness.

Cusack and colleagues have recently published a protocol for a systematic review of educational interventions aimed at improving the general public’s ability to evaluate claims about the effects of health interventions [31]. However, we have not identified any reviews of school-based interventions to improve adolescents’ abilities in critical appraisal of claims, irrespective of health topic. Therefore, our objective was to conduct a systematic review of the effectiveness of educational interventions in schools aimed at enhancing adolescents’ abilities to critically appraise health claims.

## Methods

### Protocol and registration

The review protocol was registered in the PROSPERO International prospective register of systematic reviews (identification number CRD42015017936). We followed the recommendations of the Cochrane Collaboration [32] and PRISMA checklist for reporting systematic reviews [33].

### Eligibility criteria

We included studies of adolescents aged 11 to 18 that evaluated school-based educational interventions to improve critical appraisal ability for health claims through advancing students’ knowledge about science. Eligible study designs were randomised and non-randomised controlled trials, and interrupted time series. Detailed eligibility criteria for studies are presented in Table 1.

**Table 1:** Study eligibility and exclusion criteria.

**Inclusion criteria**	
***Design***	Randomised and non-randomised controlled trials that allocated students individually or in clusters (i.e. teachers, classrooms, schools), and that used pre-test/post-test, post-test only, and interrupted time series designs
***Setting***	Middle schools, secondary schools, high schools or other equivalent educational institutions
***Participants***	Children and adolescents aged 11 to 18
***Intervention***	All types of educational interventions meant to facilitate abilities in critical appraisal of health claims^a^ by advancing knowledge about science in one or more of the following learning areas as defined by Ryder [17]: • Study design issues (e.g. experimental studies, blinding, placebos, control groups, observational studies) • Assessing the certainty of data (e.g. variability and uncertainty of measurement, estimates of measurement variability) • Interpretation of data (e.g. distinction of correlation and causation, sample size and sampling errors) • Uncertainty in science (e.g. complexity of variables, restrictions on study designs, estimates of risks) • Science communication (e.g. the role of peer review, conflicts of interest, deficiencies in media reports of research findings)
***Comparison***	All comparisons: different educational intervention; different methods of delivery, educational contents, intervention dosages, or the like; regular classes (‘usual care’); no intervention
***Outcomes***	Primary: Critical appraisal abilities within at least one of the following domains [34]: • Knowledge and understanding: retention of facts and concepts related to critical appraisal (e.g. recognise the need for control groups to justify health claims about causality; understanding that health claims can never be proven, and accordingly health decisions may be based on estimates of risk). • Skills: ability to apply knowledge (e.g. ability to judge the credibility of a media report about a health risk). • Behaviour: transferring the knowledge and skills specified above to everyday situations (e.g. when scanning Web pages for information on a health problem or lifestyle issue). Secondary: • Attitudes, values, and beliefs related to the importance and usefulness of critical appraisal to inform decisions about health. • Participation in or completion of, attendance at, and reactions to the learning experience (e.g. participation in class, time spent on class activities, and satisfaction with the educational intervention). Outcome measurements: self-report and direct measures; validated and non-validated measurement instruments.
**Exclusion criteria**	Studies • of adolescents who were in the target age range, but attending post-secondary education. • that evaluated interventions aimed at teachers, but did not measure relevant student outcomes. • for which the educational intervention was part of a complex intervention or larger study, and it was not possible to extract results from that specific intervention separately. • of regular health education interventions (e.g. teaching about the benefits of healthy eating or the dangers of smoking) • of intervention to facilitate scientific content knowledge (e.g. basic principles of gene inheritance or human organ system functioning) • of health-related media literacy interventions involving critical examination of claims without addressing the learning areas related to knowledge about science as defined above

^a^Claims about conventional medical treatments, complementary and alternative treatments, risks/harms, health conditions, diseases, and physical or mental well-being

### Information sources and search strategy

We searched the following databases from their inception through April 15, 2016: MEDLINE, Embase, PsycINFO, AMED, Cinahl, Teachers Reference Centre, LISTA, ERIC, Sociological Abstracts, Social Services Abstracts, The Cochrane Library, Science Citation Index Expanded and Social Sciences Citation Index.

To identify grey literature, we searched OpenGrey, Social Care Online, Social Science Research Network Library, and Google Scholar. Clinicaltrials.gov and the International Clinical Trials Registry Platform Search Portal were searched for ongoing studies. Additionally, we searched reference lists of relevant reviews and citations of included studies to identify other potentially relevant references.

MWG and LVN developed a highly sensitive search strategy for MEDLINE and ERIC using search terms relevant to the population and intervention. MWG modified the search strategy for the other databases, and ran all searches. A search filter was applied as appropriate. No language restrictions were applied. See S2 File for the complete search strategy.

### Study selection

One review author (MWG) performed an initial screening of references identified by the search strategy to exclude obviously irrelevant studies. In cases of doubt, references were not excluded at this stage. Two review authors (MWG and LVN) then independently screened the remaining references and checked the full text versions of potentially relevant references. Any disagreements were resolved by consensus or through a third reviewer.

### Data collection process

Two review authors (MWG and LVN) independently extracted data from included studies using a standardised data extraction form. Disagreements were resolved by consensus. When necessary, we contacted study authors for additional information.

### Data items

We extracted the following data: methods, setting, student and education provider characteristics, interventions and comparisons (e.g. learning objectives, teaching contents, frequency), outcomes, and results.

### Risk of bias in individual studies

Two review authors (MWG and LVN) independently assessed risk of bias in included studies using a modified version of the Cochrane risk of bias tool. Modifications were based on guidelines of the Cochrane Consumers and Communication Review Group [35] and the ACROBAT guidelines for non-randomised studies [36]. We assessed risk of bias in 11 domains: sequence generation, allocation concealment, comparability of baseline characteristics and outcome measurements, blinding of students and education providers, blinding of outcome assessments, departures from intended interventions, incomplete outcome data, selective outcome reporting, reliability and validity of outcome measures (assessed by ØG and LVN), and other sources of bias. Each domain was assessed as low, unclear, or high risk of bias. The risk of bias assessments were used to assess the overall certainty of evidence for each outcome (see below). We solved disagreements by consensus or through a third reviewer.

### Synthesis of results

We considered it inappropriate to conduct a meta-analysis due to differences in interventions and designs between the studies, and insufficient reporting of study results. Thus, we synthesized results descriptively. RevMan 5.3 [37] was used to recalculate effect estimates if this improved their reporting. We used GRADE (Grading of Recommendations, Assessment, Development, and Evaluation) to assess and grade the overall certainty of evidence for each outcome, taking into account risk of bias within the studies, directness of evidence, heterogeneity, precision of effect estimates, and risk of publication bias [38].

## Results

### Study selection

**Fig 1:**
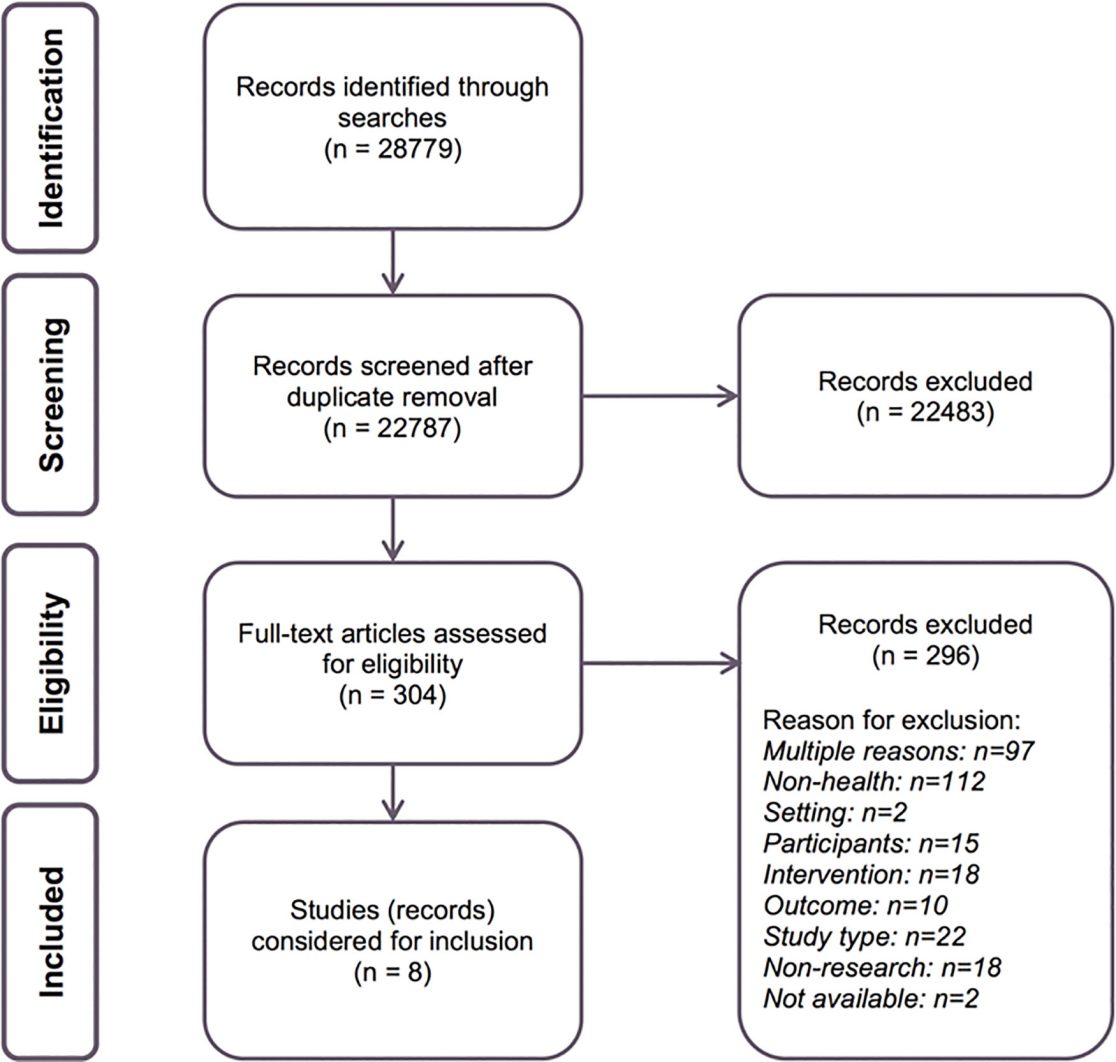
Flow chart of the search results and screening process.

The literature search identified 22787 unique references. Due to the sensitivity of the search, many of these references (11684 or 51%) were irrelevant and excluded by title only. Following title and abstract screening of the remaining 11103 references, full-texts of 304 were screened. Of these, we excluded 296 publications. We provide reasons for exclusion for the publications that would have been expected to be included, as recommended by EPOC [39] (see S1 Table). We included eight studies in the review [40–47]. Two publications represent one study: a doctoral dissertation [41] and a journal article [42]. One journal article describes two similar, but separate, studies [44]. The selection process is outlined in Fig 1.

### Study characteristics

We classified interventions across studies into two main categories: *Educational interventions comparing different teaching modalities* and *Educational interventions compared to instruction as usual*. Tables 1 and 2 provide descriptions of included studies in terms of these comparisons. Summaries of findings are provided in S2 Table. Detailed study characteristics, including risk of bias assessments, can be found in S3 Table.

**Table 2:** School-based educational interventions: comparing different teaching modalities for enhancing adolescents’ critical appraisal abilities.

Study ID [ref] *design*; Allocation unit	Setting	Students	Education providers	Intervention and comparison	Dosage	Pedagogical framework; Teaching methods	Science area (Ryder [14])	Health topics (examples)	Outcome [domain]	Type of measurement
**Hill 1998 –Part A** [42] *Randomised controlled study with post-test only*; Students	One lower secondary school, US	220 students in 7^th^ grade. Males: 48%. Age: Not reported.Ethnicity: 56% African, 40% Caucasian. SES^*^: Diverse. SP^**^: Low	Two first-year female science teachers (one per instruction group)	*Active learning (situated) instruction in causal reasoning*: Causality vs. correlation, role of random assignment, causality cues in media reports of research *Comparison*: Authoritative (abstracted) instruction; Same topics as the active learning instruction	Four 50-min lessons over 2 weeks in both teaching modalities	Active learning: Situated cognition (e.g. Vygotsky); Teacher as guide/mentor rather than lecturer; Classroom or small group discussions, reflective activities using authentic examples *Comparison*: Traditional lecture-based approach emphasising knowledge transmission; No/few reflective activities; Constructed examples	Study design; Interpreting data; Science communication	Exercise, stress	Basic knowledge and understanding of causality [Knowledge]	*Basic knowledge*: Selected-response and short-answer open-response test comprising three constructed media reports of research. Students determined cause-and-effect variables, use of random assignment, and whether a cause-effect relationship was shown. *Understanding*: Percentage score ≥80 + correct answer to open-response question about cause and effect
**Hill 1998 –Part B** [42]. *Non-randomised controlled study with post-test only*; Class periods	Same as above	194 of the 220 participating students described above.	Same as above	*Transfer instruction*: Applying causal reasoning in authentic situations (e.g. media reports of research, medical studies)*Comparison*: Active (situated) learning or authoritative (abstracted) instruction (see above) but no transfer instruction	One 50-min lesson one week after situated/abstracted instruction	Small groups, authentic examples	Same as above		Ability to scientifically evaluate claims [Skills]	Open-response test including an authentic news report from CNN (‘Grapes inhibit cancer’); Students judged believability of the claim in the story and supported their conclusions; Scale: 0 to 3 points
**Powell 2014** [47] *Non-randomised group study with pre- and post-test;* Classes	2 classes in one suburban high school, US	45 students in 9th grade. Males: Not reported. Age: Not reported. Ethnicity: 61% Caucasian, 25% Hispanic, 10% African American, 5% Asian/others. SES^*^: Diverse. SP^**^: Biology Honors students. School graduation rate 87%.	One science teacher taught both groups. BSc, 4 yrs experience	*Evaluation of Evidence Unit* Topics: Sensational scientific news, basic elements of scientific studies, making observations, evaluating evidence, peer review of research proposals, determine scientific accuracy of news reports of science *Comparison*: Some elements from the Evaluation of Evidence unit integrated into the traditional biology curriculum. Topics: Sensational scientific news, making observations, peer review	Twelve 55-min lessons over 5 weeks	Socioscientific issues (SSI) instruction: Using real-world situations to illuminate e.g. ethical dimensions of science. Teacher as mentor. Individual/group presentations and assignments, inquiry activities, discussions, use of authentic news stories, advertisements etc.*Comparison*: Non-SSI. Traditional biology curriculum introducing topics (properties of life, cells, genetics) as organised in students’ textbook.	Study design; Interpreting data; Science communication	Cancer, stem cell therapy	Ability to scientifically evaluate claims [Skills]	*Direct skills*: Constructed open-response test including a fictitious news brief that reported a scientific study about stem cell therapy. Students generated requests for information needed to judge the believability of the claim. Score: 0–34 points. *Self-reported skills*: Rating scale 0–100 points

*SES = Socioeconomic status.

**SP = School performance

#### Setting and participants

Seven of the studies took place in lower and upper secondary schools in the US [40, 41, 46, 47]; one study took place in upper secondary schools in Germany [48]. The total number of students across seven of the studies was 1148 [42–48]. One study did only provide the number of participating classes (n = 9) [40]. All studies included both female and male students, and grade levels ranged from seventh to 12th grade. Student populations in the seven US studies were ethnically diverse [40, 42–47], and the majority of the students came from low- or middle-income households [42, 43, 45, 47, 48]. In one study, the intervention and control groups comprised students from socioeconomically disadvantaged and advantaged backgrounds, respectively [44]. In the German study, the percentage with a migration background was 16%, and socioeconomic status was not reported [48]. Students’ school performance was either not reported [40, 45, 48], reported as diverse [43, 44] or reported as low [42, 46, 47]. In one study, the intervention group was students with learning disabilities whose achievement levels ranged from second to 10th grade, while the control group comprised general education students in 11th grade [46].

#### Content and delivery of interventions

Interventions addressed miscellaneous health topics, such as nutrition, exercise, cancer and smoking. They varied substantially in terms of scientific topics covered. Nonetheless, we found some similarities across studies using Ryder’s [17] framework for knowledge about science. All studies addressed aspects of study design and data interpretation, and use of control variables and differences between causality and correlation were common topics across studies. Five studies addressed science communication, most often related to deficiencies in media reports of science [40, 42, 46–48].

Pedagogical principles underpinning curriculum development and teaching methods varied across the studies. Irrespective of pedagogical perspective, all study interventions used active or dialogic approaches rather than more traditional or authoritative approaches to instruction. Active approaches took various forms such as small-group work and investigations [21, 40, 44, 45, 47, 48], worksheets [48], and teacher-guided discussions [46, 47]. Another predominant feature throughout studies was authentic problem solving to engage students in the learning process.

In general, little information was provided about education providers in terms of age, years of experience, and competence in the area studied. The researchers either delivered all or substantial parts of the interventions themselves [40, 44–46, 48], or teachers were instructed prior to the interventions [42, 43, 47]. In one study involving two teachers, the teacher who preferred an active teaching style was assigned to teach the student-centred (situated) intervention group, and the teacher who preferred a passive teaching style was assigned to the group receiving a lecture-based approach [42]. In another study, the teacher was selected to deliver the intervention because she was continuously updating herself on new pedagogical approaches with her students [47].

#### Reported outcomes

Three studies assessed knowledge and skills relevant to critical appraisal, such as understanding of epidemiological research [42, 43, 48]. Five studies assessed critical appraisal-related outcomes more directly in terms of applying causal or scientific reasoning to constructed health scenarios or actual news reports of research [40, 42, 44–47]. All studies measured outcomes immediately or shortly following interventions, and only three studies used pre- and post-intervention assessment of outcomes [40, 43, 47]. None of the studies assessed behaviour, attitudes, or satisfaction related to critical appraisal of health claims.

#### Risk of bias within studies

**Fig 2:**
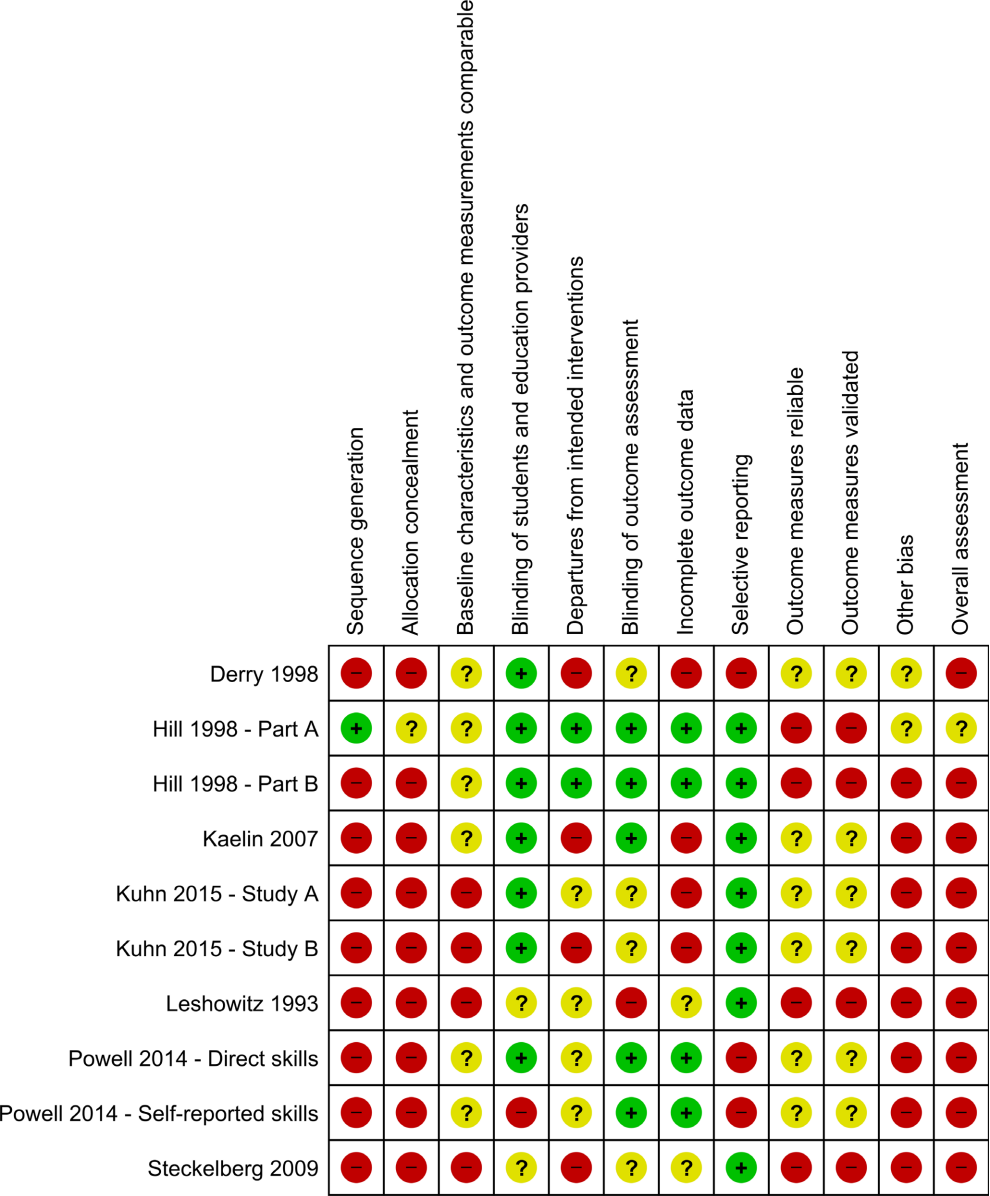
Risk of bias for each domain in included studies.

The risk of bias in the included studies is summarised in Fig 2 and S3 Table. All studies had high risk of bias in two or more key domains. In one study, individual students were randomly assigned to active (situated) learning or authoritative (abstracted) instruction in causal reasoning [42]. In the same study, some students in each of the two instructional conditions attended class periods where they received additional training about how to transfer their causal understanding to authentic health claims (transfer instruction). The researcher assigned class periods to the transfer conditions in a non-random manner (i.e. based on size of class periods). Thus, we classified this part of the study as being non-randomised. We assessed the two study parts to have moderate and high risk of bias, respectively (see Fig 2, Hill 1998 Part A and B).

In the remaining seven studies, assignment to conditions was non-random [40, 43–48]. In one study, the authors randomly allocated teachers to the intervention and control conditions, but also included a group of non-volunteer teachers whose classes participated only as controls and completed pre- and post-tests. We classified this study as a non-randomised study [43]. None of the non-randomised studies attempted to increase methodological robustness at the design level, for instance by matching groups on characteristics such as school performance. Only one study controlled for potentially confounding student factors at the analysis, including age, ethnicity, socioeconomic status, and school performance [43].

Blinding of students and education providers was generally not possible in the studies. However, because studies largely measured students’ knowledge and skills directly by testing them shortly after the end of the intervention, we assessed the risk of bias due to lack of participant blinding as low in most studies. In one study, students’ abilities to evaluate claims and evidence were measured by direct testing and self-report [47]. We assessed the two outcomes to have low and high risk of bias due to lack of blinding, respectively. Still, overall risk of bias was high for both the direct measured and the self-reported outcome.

An issue of concern was the reliability and the validity of outcome measures used in the studies. Overall, studies measured student outcomes using unique, non-standardised instruments designed for the specific interventions. We assessed the instruments across studies to have insufficient reliability, and consequently questionable validity, for the following reasons: artificially high reliability indices due to dependent items, violating the assumption of local independence and thus resulting in inefficient measures and redundancy in the data [42], small sample size [43], invalid reliability measures [45], or unacceptable scalability at the time of testing [47]. Four studies did not provide sufficient information about reliability and validity of the assessments and the data [40, 44, 45, 47].

#### Certainty of evidence

Using the GRADE criteria, we judged the certainty of evidence to be very low for all comparisons and all outcomes (See S2 Table). We downgraded the certainty of the evidence because of a high or moderate risk of bias in studies. Additionally, indirectness was a problem because studies included restricted study populations (e.g. low achievement students) or interventions (the researchers provided the instruction, not the teachers). We also downgraded because of imprecision, since most outcomes were addressed in one study only.

### Effects of educational interventions

The studies reported different summary statistics, and only four reported their results in adequate detail [42, 44, 45, 48]. For the remaining four studies, we could not calculate the effect size of the intervention as the authors did not present standard deviations and were unable to provide further data on request [40, 43, 46, 47].

#### Educational interventions comparing different teaching modalities

We identified two studies that compared different teaching modalities: a randomized controlled trial of individual 7th-grade students [42] and a non-randomised group study of two 9th-grade classes [47]. Both studies compared student-active, dialogic instruction approaches to more authoritative or textbook-oriented approaches for teaching about the evaluation of scientific evidence and appraisal of claims in the media.

Hill [42] found that 7th-grade students who engaged in active (situated) learning activities were 71% more likely to demonstrate basic knowledge of causality based on a test that comprised fictitious reports of health research compared to students who received authoritative (abstracted) instruction (RR 1.71, 95% CI: 1.35 to 2.16, p < 0.01). Among those demonstrating basic knowledge, the proportion of students who understood the concept of causality (i.e. could explain cause-effect relationships in their own words) was three times higher in the active learning group compared to the authoritative instruction group (RR 3.03, 95% CI: 1.83 to 5, p < 0.01). Sixty students in the active learning group and 34 students in the authoritative instruction group received additional training about how to transfer their causal understanding to real-life situations (transfer instruction). Only two active learning students could transfer their understanding to an authentic media report about health research two weeks after the instruction, while none of the traditional instruction students could (see Table 2).

Findings from a more recent study indicated that students exposed to active learning approaches rated their abilities to evaluate evidence significantly higher than did those exposed to traditional methods (p = 0.028, means and CIs not provided). However, when directly tested, there was no statistically significant difference between groups in their abilities to critically appraise a fictitious media report about health research (means, CIs, and p-values not provided) [46].

We graded the certainty of the evidence for the results of this comparison as very low (see S2 Table).

#### Educational interventions compared to instruction as usual

Six studies compared various educational interventions to instruction as usual. All were non-randomised controlled studies with teachers [40, 43, 46], classes [45, 47], or schools [44] as the unit of allocation. Interventions across studies varied considerably in content and dosage but they all involved science instruction in causal reasoning, including the basics of epidemiology [43] and evidence-based medicine [48] (Table 3).

**Table 3:** School-based educational interventions compared to instruction as usual for enhancing adolescents’ critical appraisal abilities.

Study ID [ref] *design*, allocation unit	Setting	Students	Education providers	Intervention	Dosage	Pedagogical framework; Teaching methods	Science area (Ryder [14])	Health topics (examples)	Outcome [domain]	Type of measurement
**Derry 1998** [40] *Non-randomised group study with pre- and post-test*; Teachers	9 classes in one lower secondary school, US	8^th^ grade (N not reported). Males: Not reported. Age: Not reported. Ethnicity: Diverse. SES^*^: Diverse. SP^**^: Not reported	One science and one social studies teacher; expert scientist; lead researchers	*Simulation gaming (role play of legislation hearing) in causal reasoning*. Topics: Single-case observation vs. RCTs, governmental regulations of scientific and lay community, valid statistical inference	Fifteen 70-min lessons over 3 weeks	Situated cognition (e.g. Vygotsky), radical constructionism; Small groups, lectures + class discussions, extensive project work, role play, teachers as mentors and models	Study design; Interpreting data; Uncertainty in science; Science communication	Cancer; dietary supplements; violence	Causal reasoning [Skills]	Test scenarios of a court trial that presented various forms of evidence and counterarguments; Students answered one open-response question that required causal reasoning (Q1), and one that did not (Q2); Q1: Scale -1 to 13 points, mean score, proportion of inappropriate responses; Q2: Proportion of responses that involved inappropriate causal reasoning
**Kaelin 2007** [43] *Non-randomised group study with pre- and post-test*; Teachers	16 lower secondary schools, US	998 students in 7th grade. Males: 47%. Age (mean): 12.2. Ethnicity: 54% Hispanic, 37% African American, 10% Caucasian, 2% Asian/Pacific. SES: Low. SP: Diverse	Six female and two male science teachers	*Epidemiology curriculum*. Topics: Descriptive and analytical epidemiology, flaws in observational studies, societal role of epidemiology, evaluating prevention strategies	34 lessons (1–2 class periods) over six months. No. of lessons taught varied (see S2 and S3 Tables)	Understanding by Design: Enduring understanding that has lasting value outside the classroom; Small groups (Epi teams investigations), lectures, worksheets, portfolios, pre/post assessments	Study design; Interpreting data; Uncertainty in science	Acne, back pain	Epidemiological knowledge and understanding [Knowledge]	*Self-reported understanding*: Likert scale, 5 to 25 points; *Direct knowledge*: Multiple-choice test, 0 to 11 points
**Kuhn 2015 Study A** [44] *Non-randomised group study with post-test only*; Schools	One public and one independent middle school, New York, US	106 students in 8th grade. Males: Not reported. Age: Not reported. Ethnicity: Diverse. SES^*^: Diverse. SP^**^: Diverse	Two of the researchers and an assistant	*Causal reasoning unit* Topics: Identifying multiple variables that may influence an outcome, the role of control groups	24 lessons over 4 weeks	No pedagogical framework specified; Class discussions, authentic data collection and analysis, report writing, blackboard logs, individual assignments.	Study design; Interpreting data; Uncertainty in science	Obesity	Causal reasoning [Skills]	Constructed open-response test including a scenario about the prevalence of cancer. Students described a study to identify potential risk factors. Proportion of students who recognised (1) the influence of multiple variables, and (2) the need for control groups
**Kuhn 2015 Study B** [45] N*on-randomised group study with post-test only*; Classes	One public middle school, New York, US	89 students in 7th grade. Males: Not reported. Age: Not reported. Ethnicity: Diverse, mainly Hispanic and African-American. SES^*^: Mainly low. SP^**^: Not reported	Science teacher taught both groups, assisted by one researcher in intervention group	Same as study A	9 lessons over 3 weeks	Same as study A	Same as study A	Same as study A	Same as study A	Same as study A
**Leshowitz 1993** [46] *Non-randomised group study with post-test only*; Teachers	5 classes in one lower and one upper secondary school, US	55 special (SE) and general education (GE) students in grades 7–12. Males: 68% (SE only). Age: 16–20. Ethnicity: 68% Caucasian, 32% Hispanic (SE only, GE reported to be similar in characteristics). SES^*^: Low. SP^**^: SE students performed at 2–10 grade level, not reported for GE students	Two pre-service special education teachers aged approx. 30 and 40 years	*Causal reasoning instruction*. Topics: Applying principles of causality to advertisements and news reports of research; Independent and dependent variables, control groups, confounding	Twenty-five 45-min lessons over 4–6 weeks	Socratic dialogue class discussions using authentic examples	Study design; Interpreting data; Uncertainty in science; Science communication	Cancer	Causal reasoning [Knowledge/Skills]	Constructed short open-response test including an advertisement and short news report of a research study; Students identified claims, graphed cause-effect variables, and explained whether data proved the claim; 0 to 6 points
**Steckelberg 2009** [48] *Non-randomised group study with post-test only*; Classes	12 classes in upper secondary schools, Germany	255 students in 11^th^ grade. Males: 38%. Age (mean): 17.5. Ethnicity: 82% had German as first language. SES^*^ and SP^**^ not reported	Two lead researchers; class teachers offered to be present	*Evidence-based medicine curriculum*. Topics: Expert vs evidence-based information, study designs (e.g. RCTs, diagnostic studies), epidemiological statistics, systematic reviews, question formulation and Internet/database searching	22 lessons over one week	Klafki’s framework for reflection of aims and instruction; Small groups, lectures, class discussions, worksheets, extensive project work using authentic examples	Study design; Assessing data certainty; Interpreting data; Uncertainty in science; Science communication	Nutrition, smoking	Understanding EBM aspects [Knowledge/Skills]	Critical Health Competence test [42]; Multiple-choice and short-answer open-response items; Total score in person parameters (Rasch model)

*SES = Socioeconomic status.

**SP = School performance.

Kaelin and colleagues [43] tested the effectiveness of an epidemiology curriculum for 7th-grade students using self-reports (questionnaire) and direct testing (multiple-choice test). Study authors provided students’ results for sub-groups only; these were mainly based on intensity (i.e. number of lessons received). The students who received more than ten lessons had small improvements in epidemiological knowledge, but not the students receiving less than 10 lessons of instruction (p < 0.05). Overall, students’ mean scores across all groups were generally low (less than 50% correct answers). These scores contrasted students’ self-reports of epidemiological understanding, which were generally high in both the intervention and control sub-groups.

Four studies evaluated instructional units aimed at improving students’ causal reasoning skills in general education [40, 44, 45] or special education [46]. All studies used open-response tests with fictitious health-related scenarios or news reports of health research to test skills. In a recent study, Kuhn and colleagues [44] evaluated an extended causal reasoning unit on 8th-graders from low socioeconomic status backgrounds attending a public school. They found that the students were almost two times more likely to recognise that multiple variables may influence cancer outcomes, when compared to a non-instructed group of students from high socioeconomic status backgrounds in an independent school (RR 1.96, 95% CI 1.32 to 2.92, p = 0.0009). Likewise, they were 51% more likely to understand the need for comparisons to make inferences about causation (RR 1.51, 95% CI 1.11 to 2.06, p = 0.009). The same authors also evaluated a short version of the same unit by comparing 7th-grade classes in the public middle school [45]. They found statistically significant results in favour of the instructed group (multiple variables RR 2.21, 95% CI 1.10 to 4.46, p = 0.03; need for comparisons RR 1.34, 95% CI 1.01 to 1.77, p = 0.04).

Similarly, Derry and colleagues [40] evaluated a simulation-based causal reasoning unit for 8th-grade students. Intervention classrooms had a higher reasoning score compared to control classrooms for the question requiring causal reasoning (mean difference in adjusted post-test scores of 1.34 points on a scale spanning -1 to 13 points, reported to be statistically significant, no CIs or p-values provided). They also provided fewer inappropriate responses, such as personal beliefs and unsubstantiated opinions, to this question than control classrooms (27% vs 43% respectively, reported to be statistically significant, no CIs or p-values provided). There were no statistically significant differences between intervention and control classrooms in proportions of inappropriate responses to a test question not requiring causal reasoning (percentages, CIs, and p-values not provided). Finally, Leshowitz and colleagues [45] found that test scores in special education students in grades 7 to 12 who received instruction in causal reasoning exceeded the scores of the control group of non-instructed general students (mean difference of 1.26 points on a scale spanning 0 to 6 points, p < 0.01).

Steckelberg and colleagues [48] pilot-tested an evidence-based medicine curriculum for 11th-grade students. Students’ competences in terms of knowledge and skills were assessed using a multiple-choice and short-answer open-response tests that measured competences in subareas such as basic statistics and experimental design [49]. The intervention group had higher competences than the control group at post-test (mean difference in person parameters of 114, 95% CI: 86 to 142, p < 0.01). A difference of 100 person parameters was considered relevant [48].

We graded the certainty of the evidence for all results within this comparison as very low (see S2 Table).

## Discussion

### Summary of evidence

We included eight studies that met the inclusion criteria [40, 42–48]. The studies reflect two lines of comparative intervention studies commonly found in the educational literature. The first line is concerned with comparing different teaching modalities [42, 47], while the second line compares a new educational intervention with instruction as usual [40, 43–46, 48]. The studies evaluated interventions that varied considerably in their scope, contents, delivery, and intensity. Studies mostly reported positive short-term effects on critical appraisal-related knowledge and skills in favour of the educational interventions. However, the certainty of the evidence for all comparisons and outcomes was very low. None of the studies measured students’ appraisal behaviour in everyday contexts outside the classroom, which would be the ultimate goal of improving students’ abilities to critically appraise health claims in society. This is perhaps not surprising given that most educational studies of students’ performance are mainly concerned with measuring cognitive learning outcomes [50].

The findings of our review are disappointing but highlight an important knowledge gap. In our digital society, there is an even greater need for teaching youth to think critically about health claims, not least due to the evolution of social media where claims are spread rapidly and have a far greater reach [51, 52]. Still, we know little about the effectiveness of educational interventions to teach adolescents critical appraisal skills, including what intervention characteristics (teaching methods, delivery, dosage, and duration) are most effective.

To our knowledge, this is the first systematic review assessing the effects of school based educational interventions to improve adolescents’ abilities in critical appraisal of health claims. We have used rigorous methods and a systematic approach, and we have performed an extensive literature search. Thus, we believe that the review makes an important contribution to the field and provide a useful summary of existing evidence for researchers and educators who plan to develop and evaluate similar interventions in the future.

### Limitations

Our review has several limitations. The heterogeneity of interventions across studies and inadequate reporting of results made it inappropriate to conduct valid meta-analyses. Only one of the studies was conducted outside the US [48].

A main reason for downgrading the quality of studies within the GRADE framework was the high risk of bias in studies, implying that we have less confidence in the results. With one exception [42], the review included non-randomised controlled studies that evaluated educational interventions among volunteer teachers or schools. The small number of studies with relatively few students, and insufficient reliability and validity of outcome measurements, were other reasons for downgrading the certainty of the evidence.

We used a comprehensive search strategy to increase the chance of finding relevant studies; however, studies could have been missed due to limitations in database interfaces, inconsistent indexing, and wrong choice of search terms. Additionally, a hand search of relevant scientific journals could have supplemented the electronic searches. The extensive search generated a vast number of references. Due to resource and time constraints, only one review author completed the preliminary screening. Even though this screening only excluded obviously irrelevant references, potentially relevant studies could have been overlooked because of screening fatigue.

We did not find sufficient numbers of studies to estimate the statistical risk of publication bias [32]. Nonetheless, publication bias might exist, as it is possible that studies showing no effect, or even a negative effect, have not been published.

### Interpretation of results in context of other evidence

Previous reviews have been unable to conclude about the effects of educational interventions on outcomes related, but not equivalent, to critical appraisal skills [24, 25, 29, 30]. The lack of well-conducted studies is an issue raised across reviews, including ours. Several reasons may explain why. Firstly, it probably reflects a general absence of randomised controlled studies in educational research [53, 54]. Bennett and colleagues [24] pointed out that participation in school-based interventions highly depends on decisions by policy makers or school departments, which complicates access to schools and force researchers to gather convenience data from entire classes in one or a few schools only. Secondly, inadequate funding of educational research may also contribute to the paucity in the literature [15, 24].

A systematic review and meta-analysis suggested that active learning strategies promote critical thinking in young people and adults [23]. The limited evidence from our review also points in this direction: most studies showed promising effects for the tested interventions; and all interventions comprised learning approaches. These approaches represent a teaching and learning style that differs from the traditional, authoritative approach familiar to many teachers and students. Previous reviews conclude that low level of engagement with tasks and inadequate or incorrect prior knowledge among students may negatively influence the uptake of active learning approaches used to promote students’ understanding of science [25, 55]. It is reasonable to assume this was an issue in the studies included in our review, although studies provided little detail of student populations to allow for analysing characteristics associated with reception and uptake of educational interventions.

In addition to challenges related to implementing new pedagogical techniques in classrooms, studies also indicated that many teachers are unacquainted with the rather sophisticated understanding of science required to critically appraise health claims [56–59]. This may explain why research staff partly or solely delivered the interventions in several of the included studies in our review. Bergsma and Carney [29] suggested that teachers may need at least a year of consistent practice to feel sufficiently prepared to teach new contents and skills to their students. Thus, teachers may need careful guidance to ensure successful implementation in classrooms.

### Implications for practice

Overall, serious limitations in the existing evidence make it difficult to draw definitive conclusions concerning the effect of school-based educational interventions for enhancing adolescents’ abilities to critically appraise health claims. Despite the discouraging results of this review, there are no grounds for discontinuing efforts in schools to increase young students’ appraisal abilities. Considering the potential for both primary gains in students’ knowledge or skills and secondary health-related gains, effective school-based interventions aimed at enhancing critical appraisal skills for health claims could have far-reaching benefits.

### Implications for research

The results of this systematic review indicate that there is a lack of school-based educational interventions for enhancing critical appraisal abilities of health claims among adolescents. Thus, novel interventions that aim to improve and sustain these abilities should be developed and evaluated. Well-designed evaluation studies are needed; preferably pragmatic cluster-randomised controlled trials that take place in a wider variety of school-based settings and that closely resemble normal educational practice [54].

Future studies that investigate instructional interventions concerned with the critical appraisal of health claims should administer interventions for a long enough duration to allow assessment of student outcomes and other factors believed to influence these outcomes. To sustain learning effects, students most likely need to practice skills over at least a semester, or even a year [53]. There is also a need for comparable, reliable, and validated outcome measures to permit firm conclusions about the effects of interventions. Notably, the instrument used in one of the studies (Critical Health Competence Test) has been further validated and improved after the specific intervention was tested [49].

To ensure ecological validity, education providers in future studies should preferably be teachers, not researchers or other experts. Educational interventions should involve an in-service component to enhance appraisal skills in teachers. While the primary goal is improving student outcomes, the impact of professional development activities on teachers’ reactions, learning, and teaching behaviour should be monitored alongside the main study [60]. A process evaluation may, for instance, include classroom observations to evaluate teacher performance and interactions with students. This will provide useful information about factors that support or hinder implementation of the intervention, how it worked, and how it might be improved.

## Supporting Information

S1 File PROSPERO protocol. (PDF)

S2 File Search strategy. (DOCX)

S1 Table Excluded studies. (DOCX)

S2 Table GRADE Summary of findings. (DOCX)

S3 Table Detailed study characteristics. (DOCX)

S4 Table PRISMA checklist. (DOCX)
