# Supplementary material for: Effects of School-Based Educational Interventions for Enhancing Adolescents Abilities in Critical Appraisal of Health Claims: A Systematic Review
Source: PLoS One. 2016 Aug 24;11(8):e0161485. doi: 10.1371/journal.pone.0161485 (PMC4996462; doi:10.1371/journal.pone.0161485)
Supplement: S1 File — (PDF) [file pone.0161485.s001.pdf]

Effects of educational interventions for enhancing adolescents abilities in critical appraisal of health claims: a systematic review

Malene Wøhlk Gundersen, Lena Nordheim, Birgitte Espehaug, Signe Flottorp

Citation

Malene Wøhlk Gundersen, Lena Nordheim, Birgitte Espehaug, Signe Flottorp. Effects of educational interventions for enhancing adolescents abilities in critical appraisal of health claims: a systematic review. PROSPERO 2015:CRD42015017936 Available from
http://www.crd.york.ac.uk/PROSPERO_REBRANDING/display_record.asp?ID=CRD42015017936

Review question(s)

How effective are school-based educational interventions to enhance adolescents' abilities in critical appraisal of health claims?

Searches

An extensive search will be conducted to identify relevant literature. Highly sensitive search strategies will be developed by combining index terms and text words relevant to the population and intervention. Search strategies will be devised and tested by an experienced medical librarian. The following databases will be searched:

Published studies: MEDLINE, EMBASE, PsycINFO, AMED (via Ovid), CINAHL, Teachers Reference Centre, LISTA (via EBSCOhost), ERIC, Sociological Abstracts, Social Science Abstracts (via ProQuest), The Cochrane Library (via Wiley), Science Citation Index Expanded and Social Sciences Citation Index (via Web of Science).

Grey literature: OpenGrey, Social Care Online, Social Science Research Network Library and Google Scholar

Ongoing studies: Clinicaltrials.gov and the International Clinical Trials Registry Platform Search

In addition, reference lists of identified relevant reviews and a citation search on included studies will be searched to identify additional potentially relevant studies.

Types of study to be included

Randomised and non-randomised controlled trials that allocated students individually or in clusters (i.e. teachers, classrooms, schools), using pre-and post-test or post-test only, and interrupted time series.

Condition or domain being studied

This review focuses on health education and the promotion of health and well-being, emphasizing critical appraisal of health information and claims in the popular media and elsewhere in society.

Participants/ population

Children and adolescents aged 11 to 18, which usually corresponds to grades 6 to 12 in middle school, secondary school, high school or other equivalent educational institutions. Studies that include 10-year olds at sixth grade or 19-year olds at 12th grade will be included. Studies aimed at teachers will be included if relevant student outcomes are measured.

Studies comprising the included age group but are undertaken in undergraduate education (e.g. college, university) will be excluded.

Intervention(s), exposure(s)

Any type of school-based educational intervention which aim to improve students' ability to critically appraise health claims and information through advancing their knowledge about science, e.g.

• Study design (e.g. experimental studies, blinding, placebos, control groups, observational studies)

• Assessing the quality of data (e.g. measurement variability)

• Interpretation of data (e.g. distinction of correlation and causation, sample size and sampling errors)

• Uncertainty in science (e.g. complexity of variables, restrictions on study designs, estimates of risks)

• Science communication (e.g. the role of peer review, funding issues, deficiencies in media reports of research findings)

The educational intervention has to involve claims and information about the human body and health, including conventional medical treatments, complementary and alternative treatments, health conditions, diseases, and physical or mental well-being. Studies where health issues are used only as a means to an end will also be included (i.e. studies that primarily aimed to enhance students' knowledge about science, and health-related cases served as examples in lessons and assessment).

No restrictions will be made with respect to teaching and learning method, educational content and materials, intervention dosage, or who administered the intervention. Studies will be included where the educational intervention is part of a complex intervention or larger study, and it is possible to separately extract results from that specific intervention.

Comparator(s)/ control

Regular classes, no intervention or another type of intervention, for example a generic health education intervention with no focus on critical appraisal.

Context

Regular school setting

Outcome(s)

Primary outcomes

Knowledge and understanding: students' retention of facts and concepts related to critical appraisal, for example recognising the need for control groups to justify a health claim about causality.

Skills: ability to apply knowledge, for example being able to identify deficiencies in a media report about a health risk.

Behaviour: transferring the knowledge and skills specified above to everyday situations, for example when sifting through web pages for information on a health problem or lifestyle issue.

Secondary outcomes

Attitudes: students' values/beliefs related to the importance and usefulness of critical appraisal to inform decisions about health.

Participation or completion, attendance at and reactions on the learning experience.

Data extraction, (selection and coding)

Two reviewers will independently extract data from included studies using a standardised data extraction form. Any disagreements will be resolved by consensus.

The following data will be extracted: methods (e.g. type of study, allocation unit), students (e.g. age, gender, ethnicity), education providers (e.g. occupation, working experience), interventions and comparisons (e.g. learning objectives, teaching contents, frequency), outcomes (e.g. type of outcome, measurement method, timing of assessment), results, and potential confounding factors (e.g. distribution across groups).

If necessary, study authors will be contacted for additional information and clarification of study methods.

Risk of bias (quality) assessment
Two reviewers will independently assess risk of bias in included studies by using a modified version of the Cochrane risk of bias tool, considering the following domains: Sequence generation, allocation concealment, comparability of baseline characteristics and outcome measurements, blinding of students and education providers, blinding of outcome assessment, departures from intended interventions, incomplete outcome data, selective outcome reporting, outcome measures reliable, and other sources of bias. Disagreements will be resolved by consensus or by involving a third reviewer.

If appropriate, sensitivity analyses will be conducted to examine the impact on effect estimates of including studies with high or unclear risk of bias.

Strategy for data synthesis
Data will be tabulated and summarised textually to create a narrative synthesis of the included studies. If studies are not considered too heterogeneous with regard to participant characteristics, interventions, and methods, meta-analyses using Cochrane Review Manager Software will be performed. We will calculate mean differences for continuous outcomes, and relative risks for dichotomous outcomes, using a 95 % confidence interval. Any randomised controlled trials will be analysed separately.

Analysis of subgroups or subsets
None planned

Dissemination plans
Findings will be disseminated at conferences and in professional and peer-reviewed journals.

Contact details for further information
Ms Nordheim

Bergen University College, Faculty of Health and Social Sciences, Centre for Evidence Based Practice

P.O. Box 7030, 5020 Bergen, NORWAY

lvn@hib.no

Organisational affiliation of the review
Centre for Evidence Based Practice, Bergen University College

Review team
Ms Malene Wøhlk Gundersen, Centre for Evidence Based Practice, Bergen University College / Norwegian Directorate of Health
Mrs Lena Nordheim, Centre for Evidence Based Practice, Bergen University College
Professor Birgitte Espehaug, Centre for Evidence Based Practice, Bergen University College
Professor Signe Flottorp, Norwegian Knowledge Centre for the Health Services / Institute of Health and Society, University of Oslo

Anticipated or actual start date
01 January 2014

Anticipated completion date
31 December 2015

Funding sources/sponsors
Bergen University College

Conflicts of interest
None known

Language
English

Country
Norway

Subject index terms status
Subject indexing assigned by CRD

Subject index terms
Adolescent; Evidence-Based Medicine; Health; Humans

Stage of review
Ongoing

Date of registration in PROSPERO
27 March 2015

Date of publication of this revision
30 March 2015

DOI
10.15124/CRD42015017936

Stage of review at time of this submission	Started	Completed
Preliminary searches	No	Yes
Piloting of the study selection process	No	Yes
Formal screening of search results against eligibility criteria	No	Yes
Data extraction	Yes	No
Risk of bias (quality) assessment	Yes	No
Data analysis	No	No

PROSPERO
International prospective register of systematic reviews
The information in this record has been provided by the named contact for this review. CRD has accepted this information in good faith and registered the review in PROSPERO. CRD bears no responsibility or liability for the content of this registration record, any associated files or external websites.
