# Supplementary material for: Effects of School-Based Educational Interventions for Enhancing Adolescents Abilities in Critical Appraisal of Health Claims: A Systematic Review
Source: PLoS One. 2016 Aug 24;11(8):e0161485. doi: 10.1371/journal.pone.0161485 (PMC4996462; doi:10.1371/journal.pone.0161485)
Supplement: S2 File — (DOCX) [file pone.0161485.s002.docx]

**S2 File. Search history**

| MEDLINE via Ovid – 15.4.2016 | | |
| --- | --- | --- |
| **Ovid MEDLINE(R) In-Process & Other Non-Indexed Citations, Ovid MEDLINE(R) Daily and Ovid MEDLINE(R) 1946 to Present** | | |
| **#** | **Searches** |  |
| 1 | (age? adj2 ("11" or "12" or "13" or "14" or "15" or "16" or "17" or "18" or eleven or twelve or thirteen or fourteen or fifteen or sixteen or seventeen or eighteen)).tw. |  |
| 2 | Schools/ |  |
| 3 | School Health Services/ |  |
| 4 | Students/ |  |
| 5 | (schoolchild* or school-child* or school student* or pupil?).tw. |  |
| 6 | ((middle or secondary or high) adj school?).tw. |  |
| 7 | ((six or sixth or seven* or eight? or nine or ninth or ten or tenth or eleven* or twelve or twelfth) adj3 grade*).tw. |  |
| 8 | (grade? 6 or grade? 7 or grade? 8 or grade? 9 or grade? 10 or grade? 11 or grade? 12).tw. |  |
| 9 | (year? 6 or year? 7 or year? 8 or year? 9 or year? 10 or year? 11 or year? 12 or year? 13).tw. |  |
| 10 | or/2-9 |  |
| 11 | Education/ |  |
| 12 | Health education/ |  |
| 13 | Health Education, Dental/ |  |
| 14 | Health Fairs/ |  |
| 15 | Sex Education/ |  |
| 16 | Patient Education as Topic/ |  |
| 17 | Education, Distance/ |  |
| 18 | Education, Nonprofessional/ |  |
| 19 | Education, Special/ |  |
| 20 | Competency-Based Education/ |  |
| 21 | Curriculum/ |  |
| 22 | exp Programmed Instruction as Topic/ |  |
| 23 | Teaching/ |  |
| 24 | exp Teaching Materials/ |  |
| 25 | exp Educational Technology/ |  |
| 26 | ed.fs. |  |
| 27 | (educat* or train* or teach* or workshop? or work-shop? or seminar? or course? or curricul* or learn* or instruct* or self-instruct* or selfinstruct* or coach* or skill* or problem-based or pedagog* or class or classes or lesson? or taught or module?).tw. |  |
| 28 | or/11-27 |  |
| 29 | Consumer Health Information/ |  |
| 30 | Information Services/ |  |
| 31 | Research/ |  |
| 32 | Empirical Research/ |  |
| 33 | Research Design/ |  |
| 34 | Research Report/ |  |
| 35 | Statistics as Topic/ |  |
| 36 | Periodicals as Topic/ |  |
| 37 | Epidemiology/ |  |
| 38 | Pharmacoepidemiology/ |  |
| 39 | Pamphlets/ |  |
| 40 | Newspaper as Topic/ |  |
| 41 | Mass Media/ |  |
| 42 | Television/ |  |
| 43 | Radio/ |  |
| 44 | exp Internet/ |  |
| 45 | or/29-44 |  |
| 46 | Information Seeking Behavior/ |  |
| 47 | Problem Solving/ |  |
| 48 | Problem-Based Learning/ |  |
| 49 | Data Interpretation, Statistical/ |  |
| 50 | exp Evidence-Based Practice/ |  |
| 51 | or/46-50 |  |
| 52 | 45 and 51 |  |
| 53 | Science/ or exp Information Literacy/ or Judgment/ or Decision Making/ or Thinking/ |  |
| 54 | 52 or 53 |  |
| 55 | ((health or information or mathematical or quantitative or science or scientific* or media) adj2 (literacy or literate? or illiteracy or illiterate?)).tw. |  |
| 56 | (information adj2 competen*).tw. |  |
| 57 | numeracy.tw. |  |
| 58 | (scientific adj2 (skill? or think* or reason*)).tw. |  |
| 59 | (critical adj2 (think* or reason*)).tw. |  |
| 60 | evidence-based.tw. |  |
| 61 | or/55-60 |  |
| 62 | health litera$2.af. |  |
| 63 | medical literacy.af. |  |
| 64 | (health and literacy).ti. |  |
| 65 | (functional and health and literacy).tw. |  |
| 66 | low-litera$2.ti. |  |
| 67 | litera$2.ti. |  |
| 68 | illitera$2.ti. |  |
| 69 | reading/ |  |
| 70 | comprehension/ |  |
| 71 | or/62-70 |  |
| 72 | *health promotion/ |  |
| 73 | *health education/ |  |
| 74 | *patient education/ |  |
| 75 | *communication barriers/ |  |
| 76 | *communication/ |  |
| 77 | *health knowledge,attitudes,practice/ |  |
| 78 | *attitude to health/ |  |
| 79 | *comprehension/ and *educational status/ |  |
| 80 | (family and literacy).ti. |  |
| 81 | (drug labeling.af. or prescriptions, drug/) and comprehension.af. |  |
| 82 | ((cancer or diabetes or genetics) and (literacy or comprehension)).ti. |  |
| 83 | (limited and (educational status or (educational and status) or literacy)).af. |  |
| 84 | (patient$1 and (educational status or (educational and status) or literacy)).af. |  |
| 85 | (patient$1 and (comprehension or understanding)).ti. |  |
| 86 | or/72-85 |  |
| 87 | 71 and 86 |  |
| 88 | 61 or 87 |  |
| 89 | ((health or consumer or medical or scien*) adj2 information).tw. |  |
| 90 | risk information.tw. |  |
| 91 | ((health or medical or scien*) adj2 (claim? or statement? or message?)).tw. |  |
| 92 | ((health or medical or scien*) adj2 (paper? or article? or report? or literature? or journal? or periodical? or research)).tw. |  |
| 93 | (media* or television* or tv or radio or broadcast* or broadsides or news* or maga?ine? or ((print* or written) adj2 information) or pamphlet? or leaflet? or booklet? or brochure?).tw. |  |
| 94 | (internet* or world wide web or worldwide web or web site? or website? or web portal? or blog* or web log* or bulletin board? or bulletinboard? or message board? or messageboard? or forum? or ehealth or e-health or electronic health or weblog* or moblog* or vlog* or video blog* or microblog* or wiki* or web page? or webpage? or chat room? or chatroom?).tw. |  |
| 95 | or/89-94 |  |
| 96 | 88 and 95 |  |
| 97 | (((critical* adj read*) or apprais* or evaluat* or understand* or assess* or judg* or interpret*) adj7 ((health or consumer or medical or scien*) adj2 information)).tw. |  |
| 98 | (((critical* adj read*) or apprais* or evaluat* or understand* or assess* or judg* or interpret*) adj7 risk information).tw. |  |
| 99 | (((critical* adj read*) or apprais* or evaluat* or understand* or assess* or judg* or interpret*) adj7 ((health or medical or scien*) adj2 (claim? or statement? or message?))).tw. |  |
| 100 | (((critical* adj read*) or apprais* or evaluat* or understand* or assess* or judg* or interpret*) adj7 ((health or medical or scien*) adj2 (paper? or article? or report? or literature? or journal? or periodical? or research))).tw. |  |
| 101 | (((critical* adj read*) or apprais* or evaluat* or understand* or assess* or judg* or interpret*) adj7 (media* or television* or tv or radio or broadcast* or broadsides or news* or maga?ine? or ((print* or written) adj2 information) or pamphlet? or leaflet? or booklet? or brochure?)).tw. |  |
| 102 | (((critical* adj read*) or apprais* or evaluat* or understand* or assess* or judg* or interpret*) adj7 (internet* or world wide web or worldwide web or web site? or website? or web portal? or blog* or web log* or bulletin board? or bulletinboard? or message board? or messageboard? or forum? or ehealth or e-health or electronic health or weblog* or moblog* or vlog* or video blog* or microblog* or wiki* or web page? or webpage? or chat room? or chatroom?)).tw. |  |
| 103 | or/54,96-102 |  |
| 104 | 1 and 28 and 103 |  |
| 105 | 10 and 103 |  |
| 106 | 104 or 105 |  |
| 107 | Randomized Controlled Trial.pt. |  |
| 108 | Controlled Clinical Trial.pt. |  |
| 109 | Multicenter Study.pt. |  |
| 110 | (randomis* or randomiz* or randomly or random allocat*).ti,ab. |  |
| 111 | (group? and (random* or between* or control* or intervent*)).ab. |  |
| 112 | (multicenter or multi center or multicentre or multi centre).ti. or trial.ti,ab. |  |
| 113 | (intervention* or controlled or control group or compare or comparison* or compared or ((prospectiv* or crossover) adj5 (study or studies or design)) or (before adj5 after) or (pre adj5 post) or pretest or pre test or posttest or post test or quasiexperiment* or quasi experiment* or evaluat* or effect? or effectiveness or impact or time series or time point? or repeated measur*).ti,ab. |  |
| 114 | Program Evaluation/ |  |
| 115 | Pilot Projects/ |  |
| 116 | or/107-115 |  |
| 117 | exp Animals/ |  |
| 118 | Humans/ |  |
| 119 | 117 not (117 and 118) |  |
| 120 | 116 not 119 |  |
| 121 | Review.pt. |  |
| 122 | Meta Analysis.pt. |  |
| 123 | News.pt. |  |
| 124 | Comment.pt. |  |
| 125 | Editorial.pt. |  |
| 126 | Letter.pt. |  |
| 127 | cochrane database of systematic reviews.jn. |  |
| 128 | comment on.cm. |  |
| 129 | (systematic review or literature review).ti. |  |
| 130 | or/121-129 |  |
| 131 | 120 not 130 |  |
| 132 | 106 and 131 |  |

| EMBASE via Ovid – 15.4.2016 | | |
| --- | --- | --- |
| **Embase 1974 to 2015 May 29 via Ovid** | | |
| **#** | **Searches** |  |
| 1 | (age? adj2 ("11" or "12" or "13" or "14" or "15" or "16" or "17" or "18" or eleven or twelve or thirteen or fourteen or fifteen or sixteen or seventeen or eighteen)).tw. |  |
| 2 | School/ |  |
| 3 | High school/ |  |
| 4 | Middle school/ |  |
| 5 | School health service/ |  |
| 6 | student/ |  |
| 7 | high school student/ |  |
| 8 | middle school student/ |  |
| 9 | (schoolchild* or school-child* or school student* or pupil?).tw. |  |
| 10 | ((middle or secondary or high) adj school?).tw. |  |
| 11 | ((six or sixth or seven* or eight? or nine or ninth or ten or tenth or eleven* or twelve or twelfth) adj3 grade*).tw. |  |
| 12 | (grade? 6 or grade? 7 or grade? 8 or grade? 9 or grade? 10 or grade? 11 or grade? 12).tw. |  |
| 13 | (year? 6 or year? 7 or year? 8 or year? 9 or year? 10 or year? 11 or year? 12 or year? 13).tw. |  |
| 14 | or/2-13 |  |
| 15 | Education/ |  |
| 16 | Course content/ |  |
| 17 | Curriculum/ |  |
| 18 | Curriculum development/ |  |
| 19 | Education program/ |  |
| 20 | Educational mobility/ |  |
| 21 | Educational model/ |  |
| 22 | Educational technology/ |  |
| 23 | exp health education/ |  |
| 24 | Interdisciplinary education/ |  |
| 25 | Learning environment/ |  |
| 26 | Problem based learning/ |  |
| 27 | Sexual education/ |  |
| 28 | exp special education/ |  |
| 29 | Student assistance program/ |  |
| 30 | Study skills/ |  |
| 31 | Teaching/ |  |
| 32 | Health education/ |  |
| 33 | Dental health education/ |  |
| 34 | nutrition education/ |  |
| 35 | School health education/ |  |
| 36 | Patient education/ |  |
| 37 | (educat* or train* or teach* or workshop? or work-shop? or seminar? or course? or curricul* or learn* or instruct* or self-instruct* or selfinstruct* or coach* or skill* or problem-based or pedagog* or class or classes or lesson? or taught or module?).tw. |  |
| 38 | or/15-37 |  |
| 39 | consumer health information/ |  |
| 40 | information service/ |  |
| 41 | science/ |  |
| 42 | research/ |  |
| 43 | empirical research/ |  |
| 44 | study design/ |  |
| 45 | pharmacoepidemiology/ |  |
| 46 | mass medium/ |  |
| 47 | television/ |  |
| 48 | telecommunication/ |  |
| 49 | internet/ |  |
| 50 | social media/ |  |
| 51 | or/39-50 |  |
| 52 | information seeking/ |  |
| 53 | problem solving/ |  |
| 54 | problem based learning/ |  |
| 55 | decision making/ |  |
| 56 | statistical analysis/ |  |
| 57 | evidence based practice/ |  |
| 58 | evidence based medicine/ |  |
| 59 | or/52-58 |  |
| 60 | 51 and 59 |  |
| 61 | *science/ or health literacy/ or information literacy/ or thinking/ or critical thinking/ |  |
| 62 | 60 or 61 |  |
| 63 | ((health or information or mathematical or quantitative or science or scientific* or media) adj2 (literacy or literate? or illiteracy or illiterate?)).tw. |  |
| 64 | (information adj2 competen*).tw. |  |
| 65 | numeracy.tw. |  |
| 66 | (scientific adj2 (skill? or think* or reason*)).tw. |  |
| 67 | (critical adj2 (think* or reason*)).tw. |  |
| 68 | evidence-based.tw. |  |
| 69 | or/63-68 |  |
| 70 | health litera$2.af. |  |
| 71 | medical literacy.af. |  |
| 72 | (health and literacy).ti. |  |
| 73 | (functional and health and literacy).tw. |  |
| 74 | low-litera$2.ti. |  |
| 75 | litera$2.ti. |  |
| 76 | illitera$2.ti. |  |
| 77 | reading/ |  |
| 78 | comprehension/ |  |
| 79 | or/70-78 |  |
| 80 | *health promotion/ |  |
| 81 | *health education/ |  |
| 82 | *patient education/ |  |
| 83 | *communication disorder/ |  |
| 84 | *interpersonal communication/ |  |
| 85 | *attitude to health/ |  |
| 86 | *comprehension/ and *educational status/ |  |
| 87 | (family and literacy).ti. |  |
| 88 | (drug labeling.af. or prescription/) and comprehension.af. |  |
| 89 | ((cancer or diabetes or genetics) and (literacy or comprehension)).ti. |  |
| 90 | (limited and (educational status or (educational and status) or literacy)).af. |  |
| 91 | (patient$1 and (educational status or (educational and status) or literacy)).af. |  |
| 92 | (patient$1 and (comprehension or understanding)).ti. |  |
| 93 | or/80-92 |  |
| 94 | 79 and 93 |  |
| 95 | 69 or 94 |  |
| 96 | ((health or consumer or medical or scien*) adj2 information).tw. |  |
| 97 | risk information.tw. |  |
| 98 | ((health or medical or scien*) adj2 (claim? or statement? or message?)).tw. |  |
| 99 | ((health or medical or scien*) adj2 (paper? or article? or report? or literature? or journal? or periodical? or research)).tw. |  |
| 100 | (media* or television* or tv or radio or broadcast* or broadsides or news* or maga?ine? or ((print* or written) adj2 information) or pamphlet? or leaflet? or booklet? or brochure?).tw. |  |
| 101 | (internet* or world wide web or worldwide web or web site? or website? or web portal? or blog* or web log* or bulletin board? or bulletinboard? or message board? or messageboard? or forum? or ehealth or e-health or electronic health or weblog* or moblog* or vlog* or video blog* or microblog* or wiki* or web page? or webpage? or chat room? or chatroom?).tw. |  |
| 102 | or/96-101 |  |
| 103 | 95 and 102 |  |
| 104 | (((critical* adj read*) or apprais* or evaluat* or understand* or assess* or judg* or interpret*) adj7 ((health or consumer or medical or scien*) adj2 information)).tw. |  |
| 105 | (((critical* adj read*) or apprais* or evaluat* or understand* or assess* or judg* or interpret*) adj7 risk information).tw. |  |
| 106 | (((critical* adj read*) or apprais* or evaluat* or understand* or assess* or judg* or interpret*) adj7 ((health or medical or scien*) adj2 (claim? or statement? or message?))).tw. |  |
| 107 | (((critical* adj read*) or apprais* or evaluat* or understand* or assess* or judg* or interpret*) adj7 ((health or medical or scien*) adj2 (paper? or article? or report? or literature? or journal? or periodical? or research))).tw. |  |
| 108 | (((critical* adj read*) or apprais* or evaluat* or understand* or assess* or judg* or interpret*) adj7 (media* or television* or tv or radio or broadcast* or broadsides or news* or maga?ine? or ((print* or written) adj2 information) or pamphlet? or leaflet? or booklet? or brochure?)).tw. |  |
| 109 | (((critical* adj read*) or apprais* or evaluat* or understand* or assess* or judg* or interpret*) adj7 (internet* or world wide web or worldwide web or web site? or website? or web portal? or blog* or web log* or bulletin board? or bulletinboard? or message board? or messageboard? or forum? or ehealth or e-health or electronic health or weblog* or moblog* or vlog* or video blog* or microblog* or wiki* or web page? or webpage? or chat room? or chatroom?)).tw. |  |
| 110 | or/62,103-109 |  |
| 111 | 1 and 38 and 110 |  |
| 112 | 14 and 110 |  |
| 113 | 111 or 112 |  |
| 114 | randomized controlled trial/ |  |
| 115 | controlled clinical trial/ |  |
| 116 | multicenter study/ |  |
| 117 | pretest posttest control group design/ |  |
| 118 | pretest posttest design/ |  |
| 119 | crossover procedure/ |  |
| 120 | experimental design/ |  |
| 121 | experimental study/ |  |
| 122 | pilot study/ |  |
| 123 | quasi experimental study/ |  |
| 124 | controlled study/ |  |
| 125 | (randomis* or randomiz* or randomly or random allocat*).ti,ab. |  |
| 126 | (group? and (random* or between* or control* or intervent*)).ab. |  |
| 127 | (multicenter or multi center or multicentre or multi centre).ti. or trial.ti,ab. |  |
| 128 | (intervention* or controlled or control group or compare or comparison* or compared or ((prospectiv* or crossover) adj5 (study or studies or design)) or (before adj5 after) or (pre adj5 post) or pretest or pre test or posttest or post test or quasiexperiment* or quasi experiment* or evaluat* or effect? or effectiveness or impact or time series or time point? or repeated measur*).ti,ab. |  |
| 129 | or/114-128 |  |
| 130 | exp animal/ |  |
| 131 | exp human/ |  |
| 132 | 130 not (130 and 131) |  |
| 133 | 129 not 132 |  |
| 134 | "review"/ |  |
| 135 | meta analysis/ |  |
| 136 | editorial/ |  |
| 137 | letter/ |  |
| 138 | ("cochrane database of systematic reviews" or "cochrane database of systematic reviews online").jn. |  |
| 139 | (systematic review or literature review).ti. |  |
| 140 | or/134-139 |  |
| 141 | 133 not 140 |  |
| 142 | 113 and 141 |  |

| PsycINFO via Ovid – 15.4.2016 | | |
| --- | --- | --- |
| **PsycINFO 1806 to May Week 4 2015 via Ovid** | | |
| **#** | **Searches** |  |
| 1 | (age? adj2 ("11" or "12" or "13" or "14" or "15" or "16" or "17" or "18" or eleven or twelve or thirteen or fourteen or fifteen or sixteen or seventeen or eighteen)).tw. |  |
| 2 | schools/ |  |
| 3 | boarding schools/ |  |
| 4 | charter schools/ |  |
| 5 | high schools/ |  |
| 6 | institutional schools/ |  |
| 7 | junior high schools/ |  |
| 8 | middle schools/ |  |
| 9 | School Based Intervention/ |  |
| 10 | students/ |  |
| 11 | high school students/ |  |
| 12 | junior high school students/ |  |
| 13 | special education students/ |  |
| 14 | middle school students/ |  |
| 15 | Intermediate School Students/ |  |
| 16 | (schoolchild* or school-child* or school student* or pupil?).ab,ti. |  |
| 17 | ((middle or secondary or high) adj school?).ab,ti. |  |
| 18 | ((six or sixth or seven* or eight? or nine or ninth or ten or tenth or eleven* or twelve or twelfth) adj3 grade*).ab,ti. |  |
| 19 | (grade? 6 or grade? 7 or grade? 8 or grade? 9 or grade? 10 or grade? 11 or grade? 12).ab,ti. |  |
| 20 | (year? 6 or year? 7 or year? 8 or year? 9 or year? 10 or year? 11 or year? 12 or year? 13).ab,ti. |  |
| 21 | or/2-20 |  |
| 22 | education/ |  |
| 23 | distance education/ |  |
| 24 | high school education/ |  |
| 25 | middle school education/ |  |
| 26 | private school education/ |  |
| 27 | public school education/ |  |
| 28 | secondary education/ |  |
| 29 | special education/ |  |
| 30 | curriculum/ |  |
| 31 | exp health education/ |  |
| 32 | physical education/ |  |
| 33 | exp psychology education/ |  |
| 34 | science education/ |  |
| 35 | social studies education/ |  |
| 36 | client education/ |  |
| 37 | programmed instruction/ |  |
| 38 | exp teaching/ |  |
| 39 | curriculum development/ |  |
| 40 | educational program planning/ |  |
| 41 | exp educational programs/ |  |
| 42 | (educat* or train* or teach* or workshop? or work-shop? or seminar? or course? or curricul* or learn* or instruct* or self-instruct* or selfinstruct* or coach* or skill* or problem-based or pedagog* or class or classes or lesson? or taught or module?).ab,ti. |  |
| 43 | or/22-42 |  |
| 44 | information services/ |  |
| 45 | sciences/ |  |
| 46 | experimental design/ |  |
| 47 | Statistical Analysis/ |  |
| 48 | Statistics/ |  |
| 49 | epidemiology/ |  |
| 50 | exp communications media/ |  |
| 51 | internet/ |  |
| 52 | or/44-51 |  |
| 53 | information seeking/ |  |
| 54 | decision making/ |  |
| 55 | problem solving/ |  |
| 56 | problem based learning/ |  |
| 57 | judgment/ |  |
| 58 | thinking/ |  |
| 59 | reasoning/ |  |
| 60 | evidence based practice/ |  |
| 61 | or/53-60 |  |
| 62 | 52 and 61 |  |
| 63 | science achievement/ or literacy/ or health literacy/ or information literacy/ or critical thinking/ or *sciences/ or *reasoning/ |  |
| 64 | 62 or 63 |  |
| 65 | ((health or information or mathematical or quantitative or science or scientific* or media) adj2 (literacy or literate? or illiteracy or illiterate?)).ab,ti. |  |
| 66 | (information adj2 competen*).ab,ti. |  |
| 67 | numeracy.ab,ti. |  |
| 68 | (scientific adj2 (skill? or think* or reason*)).ab,ti. |  |
| 69 | (critical adj2 (think* or reason*)).ab,ti. |  |
| 70 | evidence-based.ab,ti. |  |
| 71 | or/65-70 |  |
| 72 | health litera$2.af. |  |
| 73 | medical literacy.af. |  |
| 74 | (health and literacy).ti. |  |
| 75 | (functional and health and literacy).tw. |  |
| 76 | low-litera$2.ti. |  |
| 77 | litera$2.ti. |  |
| 78 | illitera$2.ti. |  |
| 79 | reading/ |  |
| 80 | comprehension/ |  |
| 81 | number comprehension/ |  |
| 82 | or/72-81 |  |
| 83 | *Health Promotion/ |  |
| 84 | *health education/ |  |
| 85 | *client education/ |  |
| 86 | *communication barriers/ |  |
| 87 | *Communication/ |  |
| 88 | *Health Knowledge/ |  |
| 89 | *Health Attitudes/ |  |
| 90 | *Health Behavior/ |  |
| 91 | *academic achievement/ |  |
| 92 | *educational attainment level/ |  |
| 93 | *comprehension/ and (*academic achievement/ or *educational attainment level/) |  |
| 94 | (family and literacy).ti. |  |
| 95 | (drug labeling.af. or prescription drugs/) and comprehension.af. |  |
| 96 | ((cancer or diabetes or genetics) and (literacy or comprehension)).ti. |  |
| 97 | (limited and (educational status or (educational and status) or literacy)).af. |  |
| 98 | (patient$1 and (educational status or (educational and status) or literacy)).af. |  |
| 99 | (patient$1 and (comprehension or understanding)).ti. |  |
| 100 | or/83-99 |  |
| 101 | 82 and 100 |  |
| 102 | 71 or 101 |  |
| 103 | ((health or consumer or medical or scien*) adj2 information).ab,ti. |  |
| 104 | risk information.ab,ti. |  |
| 105 | ((health or medical or scien*) adj2 (claim? or statement? or message?)).ab,ti. |  |
| 106 | ((health or medical or scien*) adj2 (paper? or article? or report? or literature? or journal? or periodical? or research)).ab,ti. |  |
| 107 | (media* or television* or tv or radio or broadcast* or broadsides or news* or maga?ine? or ((print* or written) adj2 information) or pamphlet? or leaflet? or booklet? or brochure?).ab,ti. |  |
| 108 | (internet* or world wide web or worldwide web or web site? or website? or web portal? or blog* or web log* or bulletin board? or bulletinboard? or message board? or messageboard? or forum? or ehealth or e-health or electronic health or weblog* or moblog* or vlog* or video blog* or microblog* or wiki* or web page? or webpage? or chat room? or chatroom?).ab,ti. |  |
| 109 | or/103-108 |  |
| 110 | 102 and 109 |  |
| 111 | (((critical* adj read*) or apprais* or evaluat* or understand* or assess* or judg* or interpret*) adj7 ((health or consumer or medical or scien*) adj2 information)).ab,ti. |  |
| 112 | (((critical* adj read*) or apprais* or evaluat* or understand* or assess* or judg* or interpret*) adj7 risk information).ab,ti. |  |
| 113 | (((critical* adj read*) or apprais* or evaluat* or understand* or assess* or judg* or interpret*) adj7 ((health or medical or scien*) adj2 (claim? or statement? or message?))).ab,ti. |  |
| 114 | (((critical* adj read*) or apprais* or evaluat* or understand* or assess* or judg* or interpret*) adj7 ((health or medical or scien*) adj2 (paper? or article? or report? or literature? or journal? or periodical? or research))).ab,ti. |  |
| 115 | (((critical* adj read*) or apprais* or evaluat* or understand* or assess* or judg* or interpret*) adj7 (media* or television* or tv or radio or broadcast* or broadsides or news* or maga?ine? or ((print* or written) adj2 information) or pamphlet? or leaflet? or booklet? or brochure?)).ab,ti. |  |
| 116 | (((critical* adj read*) or apprais* or evaluat* or understand* or assess* or judg* or interpret*) adj7 (internet* or world wide web or worldwide web or web site? or website? or web portal? or blog* or web log* or bulletin board? or bulletinboard? or message board? or messageboard? or forum? or ehealth or e-health or electronic health or weblog* or moblog* or vlog* or video blog* or microblog* or wiki* or web page? or webpage? or chat room? or chatroom?)).ab,ti. |  |
| 117 | or/64,110-116 |  |
| 118 | 1 and 43 and 117 |  |
| 119 | 21 and 117 |  |
| 120 | 118 or 119 |  |
| 121 | ("0451" or "2000").md. |  |
| 122 | between groups design/ |  |
| 123 | clinical trials/ |  |
| 124 | experimental methods/ |  |
| 125 | quasi experimental methods/ |  |
| 126 | program evaluation/ |  |
| 127 | educational program evaluation/ |  |
| 128 | treatment effectiveness evaluation/ |  |
| 129 | (randomis* or randomiz* or randomly or random allocat*).ti,ab. |  |
| 130 | (group? and (random* or between* or control* or intervent*)).ab. |  |
| 131 | (multicenter or multi center or multicentre or multi centre).ti. or trial.ti,ab. |  |
| 132 | (intervention* or controlled or control group or compare or comparison* or compared or ((prospectiv* or crossover) adj5 (study or studies or design)) or (before adj5 after) or (pre adj5 post) or pretest or pre test or posttest or post test or quasiexperiment* or quasi experiment* or evaluat* or effect? or effectiveness or impact or time series or time point? or repeated measur*).ti,ab. |  |
| 133 | or/121-132 |  |
| 134 | animal.po. |  |
| 135 | human.po. |  |
| 136 | 134 not (134 and 135) |  |
| 137 | 133 not 136 |  |
| 138 | ("0800" or "0830" or "1200").md. |  |
| 139 | ("Column/Opinion" or "Comment/Reply" or Editorial or "Erratum/Correction" or Letter or Review-Book or Review-Media or Review-Software & Other).dt. |  |
| 140 | (systematic review or literature review).ti. |  |
| 141 | or/138-140 |  |
| 142 | 137 not 141 |  |
| 143 | 120 and 142 |  |

| AMED via Ovid – 15.4.2016 | | |
| --- | --- | --- |
| **AMED (Allied and Complementary Medicine) 1985 to May 2015 via Ovid** | | |
| **#** | **Searches** |  |
| 1 | (age? adj2 ("11" or "12" or "13" or "14" or "15" or "16" or "17" or "18" or eleven or twelve or thirteen or fourteen or fifteen or sixteen or seventeen or eighteen)).ab,ti. |  |
| 2 | schools/ |  |
| 3 | school health services/ |  |
| 4 | students/ |  |
| 5 | (schoolchild* or school-child* or school student* or pupil?).ab,ti. |  |
| 6 | ((middle or secondary or high) adj school?).ab,ti. |  |
| 7 | ((six or sixth or seven* or eight? or nine or ninth or ten or tenth or eleven* or twelve or twelfth) adj3 grade*).ab,ti. |  |
| 8 | (grade? 6 or grade? 7 or grade? 8 or grade? 9 or grade? 10 or grade? 11 or grade? 12).ab,ti. |  |
| 9 | (year? 6 or year? 7 or year? 8 or year? 9 or year? 10 or year? 11 or year? 12 or year? 13).ab,ti. |  |
| 10 | or/2-9 |  |
| 11 | education/ |  |
| 12 | health education/ |  |
| 13 | sex education/ |  |
| 14 | exp patient education/ |  |
| 15 | education nonprofessional/ |  |
| 16 | exp education special/ |  |
| 17 | curriculum/ |  |
| 18 | teaching/ |  |
| 19 | (educat* or train* or teach* or workshop? or work-shop? or seminar? or course? or curricul* or learn* or instruct* or self-instruct* or selfinstruct* or coach* or skill* or problem-based or pedagog* or class or classes or lesson? or taught or module?).ab,ti. |  |
| 20 | or/11-19 |  |
| 21 | exp information services/ |  |
| 22 | research/ |  |
| 23 | exp models theoretical/ |  |
| 24 | research design/ |  |
| 25 | exp statistics/ |  |
| 26 | epidemiology/ |  |
| 27 | exp communications media/ |  |
| 28 | internet/ |  |
| 29 | or/21-28 |  |
| 30 | problem solving/ |  |
| 31 | learning/ |  |
| 32 | evidence based medicine/ |  |
| 33 | or/30-32 |  |
| 34 | 29 and 33 |  |
| 35 | science/ or exp decision making/ or thinking/ |  |
| 36 | 34 or 35 |  |
| 37 | ((health or information or mathematical or quantitative or science or scientific* or media) adj2 (literacy or literate? or illiteracy or illiterate?)).ab,ti. |  |
| 38 | (information adj2 competen*).ab,ti. |  |
| 39 | numeracy.ab,ti. |  |
| 40 | (scientific adj2 (skill? or think* or reason*)).ab,ti. |  |
| 41 | (critical adj2 (think* or reason*)).ab,ti. |  |
| 42 | evidence-based.ab,ti. |  |
| 43 | or/37-42 |  |
| 44 | health litera$2.af. |  |
| 45 | medical literacy.af. |  |
| 46 | (health and literacy).ti. |  |
| 47 | (functional and health and literacy).tw. |  |
| 48 | low-litera$2.ti. |  |
| 49 | litera$2.ti. |  |
| 50 | illitera$2.ti. |  |
| 51 | reading/ |  |
| 52 | or/44-51 |  |
| 53 | Health promotion/ |  |
| 54 | Health education/ |  |
| 55 | Patient education/ |  |
| 56 | communication/ |  |
| 57 | attitude to health/ |  |
| 58 | Educational status/ |  |
| 59 | (family and literacy).ti. |  |
| 60 | ((cancer or diabetes or genetics) and (literacy or comprehension)).ti. |  |
| 61 | (limited and (educational status or (educational and status) or literacy)).af. |  |
| 62 | (patient$1 and (educational status or (educational and status) or literacy)).af. |  |
| 63 | (patient$1 and (comprehension or understanding)).ti. |  |
| 64 | or/53-63 |  |
| 65 | 52 and 64 |  |
| 66 | 43 or 65 |  |
| 67 | ((health or consumer or medical or scien*) adj2 information).ab,ti. |  |
| 68 | risk information.ab,ti. |  |
| 69 | ((health or medical or scien*) adj2 (claim? or statement? or message?)).ab,ti. |  |
| 70 | ((health or medical or scien*) adj2 (paper? or article? or report? or literature? or journal? or periodical? or research)).ab,ti. |  |
| 71 | (media* or television* or tv or radio or broadcast* or broadsides or news* or maga?ine? or ((print* or written) adj2 information) or pamphlet? or leaflet? or booklet? or brochure?).ab,ti. |  |
| 72 | (internet* or world wide web or worldwide web or web site? or website? or web portal? or blog* or web log* or bulletin board? or bulletinboard? or message board? or messageboard? or forum? or ehealth or e-health or electronic health or weblog* or moblog* or vlog* or video blog* or microblog* or wiki* or web page? or webpage? or chat room? og chatroom?).ab,ti. |  |
| 73 | or/67-72 |  |
| 74 | 66 and 73 |  |
| 75 | (((critical* adj read*) or apprais* or evaluat* or understand* or assess* or judg* or interpret*) adj7 ((health or consumer or medical or scien*) adj2 information)).ab,ti. |  |
| 76 | (((critical* adj read*) or apprais* or evaluat* or understand* or assess* or judg* or interpret*) adj7 risk information).ab,ti. |  |
| 77 | (((critical* adj read*) or apprais* or evaluat* or understand* or assess* or judg* or interpret*) adj7 ((health or medical or scien*) adj2 (claim? or statement? or message?))).ab,ti. |  |
| 78 | (((critical* adj read*) or apprais* or evaluat* or understand* or assess* or judg* or interpret*) adj7 ((health or medical or scien*) adj2 (paper? or article? or report? or literature? or journal? or periodical? or research))).ab,ti. |  |
| 79 | (((critical* adj read*) or apprais* or evaluat* or understand* or assess* or judg* or interpret*) adj7 (media* or television* or tv or radio or broadcast* or broadsides or news* or maga?ine? or ((print* or written) adj2 information) or pamphlet? or leaflet? or booklet? or brochure?)).ab,ti. |  |
| 80 | (((critical* adj read*) or apprais* or evaluat* or understand* or assess* or judg* or interpret*) adj7 (internet* or world wide web or worldwide web or web site? or website? or web portal? or blog* or web log* or bulletin board? or bulletinboard? or message board? or messageboard? or forum? or ehealth or e-health or electronic health or weblog* or moblog* or vlog* or video blog* or microblog* or wiki* or web page? or webpage? or chat room? or chatroom?)).ab,ti. |  |
| 81 | or/36,74-80 |  |
| 82 | 1 and 20 and 81 |  |
| 83 | 10 and 81 |  |
| 84 | 82 or 83 |  |
| 85 | randomized controlled trials/ |  |
| 86 | comparative study/ |  |
| 87 | clinical trials/ |  |
| 88 | program evaluation/ |  |
| 89 | follow up studies/ |  |
| 90 | longitudinal studies/ |  |
| 91 | prospective studies/ |  |
| 92 | pilot projects/ |  |
| 93 | (randomis* or randomiz* or randomly or random allocat*).ti,ab. |  |
| 94 | (group? and (random* or between* or control* or intervent*)).ab. |  |
| 95 | (multicenter or multi center or multicentre or multi centre).ti. or trial.ti,ab. |  |
| 96 | (intervention* or controlled or control group or compare or comparison* or compared or ((prospectiv* or crossover) adj5 (study or studies or design)) or (before adj5 after) or (pre adj5 post) or pretest or pre test or posttest or post test or quasiexperiment* or quasi experiment* or evaluat* or effect? or effectiveness or impact or time series or time point? or repeated measur*).ti,ab. |  |
| 97 | or/85-96 |  |
| 98 | 84 and 97 |  |

| Cochrane Central Register of Controlled Trials (CENTRAL) via The Cochrane Library (Wiley) – 15.4.2016 | | |
| --- | --- | --- |
|  |  |  |
| **ID** | **Search** |  |
| #1 | (age* near/2 (11 or 12 or 13 or 14 or 15 or 16 or 17 or 18 or eleven or twelve or thirteen or fourteen or fifteen or sixteen or seventeen or eighteen)):ti,ab |  |
| #2 | MeSH descriptor: [Schools] this term only |  |
| #3 | MeSH descriptor: [School Health Services] this term only |  |
| #4 | MeSH descriptor: [Students] this term only |  |
| #5 | (schoolchild* or school-child* or school student* or pupil?):ti,ab |  |
| #6 | ((middle or secondary or high) next school?):ti,ab |  |
| #7 | ((six or sixth or seven* or eight? or nine or ninth or ten or tenth or eleven* or twelve or twelfth) near/3 grade*):ti,ab |  |
| #8 | ((grade*) next (6 or 7 or 8 or 9 or 10 or 11 or 12)):ti,ab |  |
| #9 | ((year*) next (6 or 7 or 8 or 9 or 10 or 11 or 12 or 13)):ti,ab |  |
| #10 | #2 or #3 or #4 or #5 or #6 or #7 or #8 or #9 |  |
| #11 | MeSH descriptor: [Education] this term only |  |
| #12 | MeSH descriptor: [Health Education] this term only |  |
| #13 | MeSH descriptor: [Health Education, Dental] this term only |  |
| #14 | MeSH descriptor: [Health Fairs] this term only |  |
| #15 | MeSH descriptor: [Sex Education] this term only |  |
| #16 | MeSH descriptor: [Patient Education as Topic] this term only |  |
| #17 | MeSH descriptor: [Education, Distance] this term only |  |
| #18 | MeSH descriptor: [Education, Nonprofessional] this term only |  |
| #19 | MeSH descriptor: [Education, Special] this term only |  |
| #20 | MeSH descriptor: [Competency-Based Education] this term only |  |
| #21 | MeSH descriptor: [Programmed Instruction as Topic] explode all trees |  |
| #22 | MeSH descriptor: [Teaching] this term only |  |
| #23 | MeSH descriptor: [Curriculum] this term only |  |
| #24 | MeSH descriptor: [Teaching Materials] explode all trees |  |
| #25 | MeSH descriptor: [Educational Technology] explode all trees |  |
| #26 | Any MeSH descriptor with qualifier(s): [Education - ED] |  |
| #27 | (educat* or train* or teach* or workshop? or work-shop? or seminar? or course? or curricul* or learn* or instruct* or self-instruct* or selfinstruct* or coach* or skill* or problem-based or pedagog* or class or classes or lesson? or taught or module?):ti,ab |  |
| #28 | #11 or #12 or #13 or #14 or #15 or #16 or #17 or #18 or #19 or #20 or #21 or #22 or #23 or #24 or #25 or #26 or #27 |  |
| #29 | MeSH descriptor: [Consumer Health Information] explode all trees |  |
| #30 | MeSH descriptor: [Information Services] this term only |  |
| #31 | MeSH descriptor: [Research] this term only |  |
| #32 | MeSH descriptor: [Empirical Research] this term only |  |
| #33 | MeSH descriptor: [Research Design] this term only |  |
| #34 | MeSH descriptor: [Research Report] this term only |  |
| #35 | MeSH descriptor: [Statistics as Topic] this term only |  |
| #36 | MeSH descriptor: [Periodicals as Topic] this term only |  |
| #37 | MeSH descriptor: [Epidemiology] this term only |  |
| #38 | MeSH descriptor: [Pharmacoepidemiology] this term only |  |
| #39 | MeSH descriptor: [Pamphlets] this term only |  |
| #40 | MeSH descriptor: [Newspapers as Topic] this term only |  |
| #41 | MeSH descriptor: [Mass Media] this term only |  |
| #42 | MeSH descriptor: [Television] this term only |  |
| #43 | MeSH descriptor: [Radio] this term only |  |
| #44 | MeSH descriptor: [Internet] explode all trees |  |
| #45 | #29 or #30 or #31 or #32 or #33 or #34 or #35 or #36 or #37 or #38 or #39 or #40 or #41 or #42 or #43 or #44 |  |
| #46 | MeSH descriptor: [Information Seeking Behavior] this term only |  |
| #47 | MeSH descriptor: [Problem Solving] this term only |  |
| #48 | MeSH descriptor: [Problem-Based Learning] this term only |  |
| #49 | MeSH descriptor: [Data Interpretation, Statistical] this term only |  |
| #50 | MeSH descriptor: [Evidence-Based Practice] explode all trees |  |
| #51 | #46 or #47 or #48 or #49 or #50 |  |
| #52 | #45 and #51 |  |
| #53 | MeSH descriptor: [Science] this term only |  |
| #54 | MeSH descriptor: [Information Literacy] explode all trees |  |
| #55 | MeSH descriptor: [Judgment] this term only |  |
| #56 | MeSH descriptor: [Decision Making] this term only |  |
| #57 | MeSH descriptor: [Thinking] this term only |  |
| #58 | #52 or #53 or #54 or #55 or #56 or #57 |  |
| #59 | ((health or information or mathematical or quantitative or science or scientific* or media) near/2 (literacy or literate? or illiteracy or illiterate?)):ti,ab |  |
| #60 | (information near/2 competen*):ti,ab |  |
| #61 | numeracy:ti,ab |  |
| #62 | (scientific near/2 (skill? or think* or reason*)):ti,ab |  |
| #63 | (critical near/2 (think* or reason*)):ti,ab |  |
| #64 | evidence-based:ti,ab |  |
| #65 | #59 or #60 or #61 or #62 or #63 or #64 |  |
| #66 | health next litera* |  |
| #67 | medical next literacy |  |
| #68 | (health and literacy):ti |  |
| #69 | (functional health literacy):ti,ab,kw |  |
| #70 | low-litera*:ti |  |
| #71 | litera*:ti |  |
| #72 | illitera*:ti |  |
| #73 | MeSH descriptor: [Reading] this term only |  |
| #74 | MeSH descriptor: [Comprehension] this term only |  |
| #75 | #66 or 70 or #68 or #69 or #70 or #71 or #72 or #73 or #74 |  |
| #76 | MeSH descriptor: [Health Promotion] this term only |  |
| #77 | MeSH descriptor: [Health Education] this term only |  |
| #78 | MeSH descriptor: [Patient Education as Topic] this term only |  |
| #79 | MeSH descriptor: [Communication Barriers] this term only |  |
| #80 | MeSH descriptor: [Communication] this term only |  |
| #81 | MeSH descriptor: [Health Knowledge, Attitudes, Practice] this term only |  |
| #82 | MeSH descriptor: [Attitude to Health] this term only |  |
| #83 | MeSH descriptor: [Comprehension] this term only |  |
| #84 | MeSH descriptor: [Educational Status] this term only |  |
| #85 | #83 and #84 |  |
| #86 | (family and literacy):ti |  |
| #87 | drug labeling:ti,ab,kw |  |
| #88 | MeSH descriptor: [Prescription Drugs] this term only |  |
| #89 | comprehension:ti,ab,kw |  |
| #90 | (#87 or #88) and #89 |  |
| #91 | ((cancer or diabetes or genetics) and (literacy or comprehension)):ti |  |
| #92 | (limited and (educational status or (educational and status) or literacy)):ti,ab,kw |  |
| #93 | (patient* and (educational status or (educational and status) or literacy)):ti,ab,kw |  |
| #94 | (patient* and (comprehension or understanding)):ti,ab,kw |  |
| #95 | #76 or #77 or #78 or #79 or #80 or #81 or #82 or #85 or #86 or #90 or #91 or #92 or #93 or #94 |  |
| #96 | #75 and #95 |  |
| #97 | #65 or #96 |  |
| #98 | ((health or consumer or medical or scien*) near/2 information):ti,ab |  |
| #99 | risk next information:ti,ab |  |
| #100 | ((health or medical or scien*) near/2 (claim? or statement? or message?)):ti,ab |  |
| #101 | ((health or medical or scien*) near/2 (paper? or article? or report? or literature? or journal? or periodical? or research)):ti,ab |  |
| #102 | (media* or television* or tv or radio or broadcast* or broadsides or news* or maga?ine? or ((print* or written) near/2 information) or pamphlet? or leaflet? or booklet? or brochure?):ti,ab |  |
| #103 | (internet* or (world next wide next web) or (worldwide next web) or (web next site?) or website? or (web next portal?) or blog* or (web next log*) or (bulletin next board?) or bulletinboard? or (message next board?) or messageboard? or forum? or ehealth or e-health or (electronic next health) or weblog* or moblog* or vlog* or video blog* or microblog* or wiki* or (web next page?) or webpage? or (chat next room?) or chatroom?):ti,ab |  |
| #104 | #98 or #99 or #100 or #101 or #102 or #103 |  |
| #105 | #97 and #104 |  |
| #106 | (((critical* next read*) or apprais* or evaluat* or understand* or assess* or judg* or interpret*) near/7 ((health or consumer or medical or scien*) near/2 information)):ti,ab |  |
| #107 | (((critical* next read*) or apprais* or evaluat* or understand* or assess* or judg* or interpret*) near/7 risk information):ti,ab |  |
| #108 | (((critical* next read*) or apprais* or evaluat* or understand* or assess* or judg* or interpret*) near/7 ((health or medical or scien*) near/2 (claim? or statement? or message?))):ti,ab |  |
| #109 | (((critical* next read*) or apprais* or evaluat* or understand* or assess* or judg* or interpret*) near/7 ((health or medical or scien*) near/2 (paper? or article? or report? or literature? or journal? or periodical? or research))):ti,ab |  |
| #110 | (((critical* next read*) or apprais* or evaluat* or understand* or assess* or judg* or interpret*) near/7 (media* or television* or tv or radio or broadcast* or broadsides or news* or maga?ine? or ((print* or written) near/2 information) or pamphlet? or leaflet? or booklet? or brochure?)):ti,ab |  |
| #111 | (((critical* next read*) or apprais* or evaluat* or understand or assess* or judg* or interpret*) near/7 (internet* or (world next wide next web) or (worldwide next web) or (web next site?) or website? or (web next portal?) or blog* or (web next log*) or (bulletin next board?) or bulletinboard? or (message next board?) or messageboard? or forum? or ehealth or e-health or (electronic next health) or weblog* or moblog* or vlog* or video blog* or microblog* or wiki* or (web next page?) or webpage? or (chat next room?) or chatroom?)):ti,ab |  |
| #112 | #58 or #105 or #106 or #107 or #108 or #109 or #110 or #111 |  |
| #113 | #1 and #28 and #112 |  |
| #114 | #10 and #112 |  |

| Education Resource Information Center (ERIC) via EBSCOhost – 3.6.2015 | | |
| --- | --- | --- |
|  | | |
| **#** | **Query** |  |
| S1 | DE "Middle Schools" |  |
| S2 | DE "Secondary Schools" |  |
| S3 | DE "High Schools" |  |
| S4 | DE "Vocational High Schools" |  |
| S5 | DE "Junior High Schools" |  |
| S6 | DE "Elementary Secondary Education" |  |
| S7 | DE "Elementary Education" |  |
| S8 | DE "Secondary Education" |  |
| S9 | DE "College Preparation" |  |
| S10 | DE "Elementary School Curriculum" |  |
| S11 | DE "Elementary School Science" |  |
| S12 | DE "Secondary School Curriculum" |  |
| S13 | DE "Secondary School Science" |  |
| S14 | DE "Intermediate Grades" |  |
| S15 | DE "Grade 10" OR DE "Grade 11" OR DE "Grade 12" OR DE "Grade 6" OR DE "Grade 7" OR DE "Grade 8" OR DE "Grade 9" |  |
| S16 | Limiters - Educational Level: Elementary Education, Elementary Secondary Education, Grade 6, Grade 7, Grade 8, Grade 9, Grade 10, Grade 11, Grade 12, High Schools, Intermediate Grades, Junior High Schools, Middle Schools, Secondary Education |  |
| S17 | TI (schoolchild* or school-child* or school student# or pupil#) OR AB (schoolchild* or school-child* or school student# or pupil#) |  |
| S18 | TI ((middle or secondary or high ) N0 School#) OR AB ((middle or secondary or high) N0 School#) |  |
| S19 | TI ((six or sixth or seven* or eight# or nine or ninth or ten or tenth or eleven* or twelve or twelfth) N0 grade#) OR AB ((six or sixth or seven* or eight# or nine or ninth or ten or tenth or eleven* or twelve or twelfth) N0 grade#) |  |
| S20 | TI ( (grade# 6 or year 6 or grade# 7 or year 7 or grade# 8 or year 8 or grade# 9 or year 9 or grade# 10 or year 10 or grade# 11 or year 11 or grade# 12 or year 12 or year 13) ) OR AB ( (grade# 6 or year 6 or grade# 7 or year 7 or grade# 8 or year 8 or grade# 9 or year 9 or grade# 10 or year 10 or grade# 11 or year 11 or grade# 12 or year 12 or year 13) ) |  |
| S21 | TI (age# N2 ("11" or "12" or "13" or "14" or "15" or "16" or "17" or "18" or eleven or twelve or thirteen or fourteen or fifteen or sixteen or seventeen or eighteen)) OR AB (age# N2 ("11" or "12" or "13" or "14" or "15" or "16" or "17" or "18" or eleven or twelve or thirteen or fourteen or fifteen or sixteen or seventeen or eighteen)) |  |
| S22 | S1 OR S2 OR S3 OR S4 OR S5 OR S6 OR S7 OR S8 OR S9 OR S10 OR S11 OR S12 OR S13 OR S14 OR S15 OR S16 OR S17 OR S18 OR S19 OR S20 OR S21 |  |
| S23 | DE "Health Education" |  |
| S24 | DE "Comprehensive School Health Education" |  |
| S25 | DE "Alcohol Education" |  |
| S26 | DE "Drug Education" |  |
| S27 | DE "Physical Education" |  |
| S28 | DE "Sex Education" |  |
| S29 | DE "Patient Education" |  |
| S30 | DE "Nutrition Instruction" |  |
| S31 | DE "Foods Instruction" |  |
| S32 | DE "Home Economics" |  |
| S33 | DE "School Health Services" |  |
| S34 | DE "Controversial Issues (Course Content)" |  |
| S35 | DE "Health Materials" |  |
| S36 | DE "Evidence" |  |
| S37 | DE "Information Services" |  |
| S38 | DE "Community Information Services" |  |
| S39 | DE "Information Dissemination" |  |
| S40 | DE "Information Sources" |  |
| S41 | DE "Information Utilization" |  |
| S42 | DE "Evaluation Utilization" |  |
| S43 | DE "Research Utilization" |  |
| S44 | DE "Epidemiology" |  |
| S45 | DE "Research" |  |
| S46 | DE "Medical Research" |  |
| S47 | DE "Scientific Research" |  |
| S48 | DE "Research Design" |  |
| S49 | DE "Research Reports" |  |
| S50 | DE "Scientific and Technical Information" |  |
| S51 | DE "Science Materials" |  |
| S52 | DE "Sciences" |  |
| S53 | DE "Statistics" |  |
| S54 | DE "Periodicals" |  |
| S55 | DE "Electronic Journals" |  |
| S56 | DE "Journal Articles" |  |
| S57 | DE "Pamphlets" |  |
| S58 | DE "Mass Media" |  |
| S59 | DE "Newspapers" |  |
| S60 | DE "News Media" |  |
| S61 | DE "Newsletters" |  |
| S62 | DE "Radio" |  |
| S63 | DE "Educational Radio" |  |
| S64 | DE "Television" |  |
| S65 | DE "Educational Television" |  |
| S66 | DE "Public Television" |  |
| S67 | DE "Internet" |  |
| S68 | DE "Web 2.0 Technologies" |  |
| S69 | DE "Printed Materials" |  |
| S70 | DE "Reference Materials" |  |
| S71 | S23 OR S24 OR S25 OR S26 OR S27 OR S28 OR S29 OR S30 OR S31 OR S32 OR S33 OR S34 OR S35 OR S36 OR S37 OR S38 OR S39 OR S40 OR S41 OR S42 OR S43 OR S44 OR S45 OR S46 OR S47 OR S48 OR S49 OR S50 OR S51 OR S52 OR S53 OR S54 OR S55 OR S56 OR S57 OR S58 OR S59 OR S60 OR S61 OR S62 OR S63 OR S64 OR S65 OR S66 OR S67 OR S68 OR S69 OR S70 |  |
| S72 | DE "Credibility" |  |
| S73 | DE "Critical Thinking" |  |
| S74 | DE "Evaluative Thinking" |  |
| S75 | DE "Thinking Skills" |  |
| S76 | DE "Logical Thinking" |  |
| S77 | DE "Critical Literacy" |  |
| S78 | DE "Critical Viewing" |  |
| S79 | DE "Critical Reading" |  |
| S80 | DE "Criticism" |  |
| S81 | DE "Information Literacy" |  |
| S82 | DE "Information Skills" |  |
| S83 | DE "Media Literacy" |  |
| S84 | DE "Numeracy" |  |
| S85 | DE "Data Interpretation" |  |
| S86 | DE "Scientific Literacy" |  |
| S87 | DE "Science Process Skills" |  |
| S88 | DE "Scientific Principles" |  |
| S89 | DE "Problem Solving" |  |
| S90 | DE "Problem Based Learning" |  |
| S91 | DE "Decision Making Skills" |  |
| S92 | S72 OR S73 OR S74 OR S75 OR S76 OR S77 OR S78 OR S79 OR S80 OR S81 OR S82 OR S83 OR S84 OR S85 OR S86 OR S87 OR S88 OR S89 OR S90 OR S91 |  |
| S93 | TI ((health or information or mathematical or quantitative or science or scientific* or media) N2 (literacy or literate* or illiteracy or illiterate*)) OR AB ((health or information or mathematical or quantitative or science or scientific* or media) N2 (literacy or literate* or illiteracy or illiterate*)) |  |
| S94 | TI (information N2 competen*) OR AB (information N2 competen*) |  |
| S95 | TI numeracy OR AB numeracy |  |
| S96 | TI ( (scientific N2 (skill* or think* or reason*)) ) OR AB ( (scientific N2 (skill* or think* or reason*)) ) |  |
| S97 | TI evidence-based OR AB evidence-based |  |
| S98 | TI (critical N2 (think* or reason*)) OR AB (critical N2 (think* or reason*)) |  |
| S99 | S93 OR S94 OR S95 OR S96 OR S97 OR S98 |  |
| S100 | TI ((health or consumer or medical or scien*) N2 information) OR AB ((health or consumer or medical or scien*) N2 information) |  |
| S101 | TI risk information OR AB risk information |  |
| S102 | TI ( ((health or medical or scien*) N2 (claim# or statement* or message*)) ) OR AB ( ((health or medical or scien*) N2 (claim# or statement* or message*)) ) |  |
| S103 | TI ( ((health or medical or scien*) N0 (paper# or article* or report# or literature* or journal# or periodical# or research)) ) OR AB ( ((health or medical or scien*) N0 (paper# or article* or report# or literature* or journal# or periodical# or research)) ) |  |
| S104 | TI ((media* or television* or tv or radio or broadcast* or broadsides or news* or magazine# or magasine# or ((print* or written) N2 information) or pamphlet# or leaflet# or booklet# or brochure#) ) OR AB ( (media* or television* or tv or radio* or broadcast* or broadsides or news* or magazine# or magasine# or ((print* or written) N2 information) or pamphlet# or leaflet# or booklet# or brochure#) ) |  |
| S105 | TI ( (internet* or world wide web or worldwide web or web site# or website# or web page# or webpage# or web portal# or blog* or web log* or bulletin board# or bulletinboard# or message board# or messageboard# or forum* or ehealth or e-health or electronic health or weblog* or moblog* or vlog* or video blog* or microblog* or wiki* or chat room# or chatroom#) ) OR AB ( (internet* or world wide web or worldwide web or web site# or website# or web page# or webpage# or web portal# or blog* or web log* or bulletin board# or bulletinboard# or message board# or messageboard# or forum* or ehealth or e-health or electronic health or weblog* or moblog* or vlog* or video blog* or microblog* or wiki* or chat room# or chatroom#) ) |  |
| S106 | S100 OR S101 OR S102 OR S103 OR S104 OR S105 |  |
| S107 | S99 AND S106 |  |
| S108 | TI (((critical* N0 read*) or apprais* or evaluat* or understand* or assess* or judg* or interpret*) N10 ((health or consumer or medical or scien*) N2 information)) OR AB (((critical* N0 read*) or apprais* or evaluat* or understand* or assess* or judg* or interpret*) N10 ((health or consumer or medical or scien*) N2 information)) |  |
| S109 | TI (((critical* N0 read*) or apprais* or evaluat* or understand* or assess* or judg* or interpret*) N10 risk information) OR AB (((critical* N0 read*) or apprais* or evaluat* or understand* or assess* or judg* or interpret*) N10 risk information) |  |
| S110 | TI ( (((critical* N0 read*) or apprais* or evaluat* or understand* or assess* or judg* or interpret*) N10 ((health or medical or scien*) N0 (claim# or statement* or message*))) ) OR AB ( (((critical* N0 read*) or apprais* or evaluat* or understand* or assess* or judg* or interpret*) N10 ((health or medical or scien*) N0 (claim# or statement* or message*))) ) |  |
| S111 | TI ( (((critical* N0 read*) or apprais* or evaluat* or understand* or assess* or judg* or interpret*) N10 ((health or medical or scien*) N0 (paper# or article* or report# or literature* or journal# or periodical# or research))) ) OR AB ( (((critical* N0 read*) or apprais* or evaluat* or understand* or assess* or judg* or interpret*) N10 ((health or medical or scien*) N0 (paper# or article* or report# or literature* or journal# or periodical# or research))) ) |  |
| S112 | TI (((critical* N0 read*) or apprais* or evaluat* or understand* or assess* or judg* or interpret*) N10 (media* or television* or tv or radio or broadcast* or broadsides or news* or magazine# or magasine# or ((print* or written) N2 information) or pamphlet# or leaflet# or booklet# or brochure#)) OR AB (((critical* N0 read*) or apprais* or evaluat* or understand* or assess* or judg* or interpret*) N10 (media* or television* or tv or radio or broadcast* or broadsides or news* or magazine# or magasine# or ((print* or written) N2 information) or pamphlet# or leaflet# or booklet# or brochure#)) |  |
| S113 | TI ( (((critical* N0 read*) or apprais* or evaluat* or understand* or assess* or judg* or interpret*) N10 (internet* or world wide web or worldwide web or web site# or website# or web page# or web page# or web portal# or blog* or web log* or bulletin board# or bulletinboard# or message board# or messageboard# or forum* or ehealth or e-health or electronic health or weblog* or moblog* or vlog* or video blog* or microblog* or wiki* or chat room# or chatroom#)) ) OR AB ( (((critical* N0 read*) or apprais* or evaluat* or understand* or assess* or judg* or interpret*) N10 (internet* or world wide web or worldwide web or web site# or website# or web page# or web page# or web portal# or blog* or web log* or bulletin board# or bulletinboard# or message board# or messageboard# or forum* or ehealth or e-health or electronic health or weblog* or moblog* or vlog* or video blog* or microblog* or wiki* or chat room# or chatroom#)) ) |  |
| S114 | (S71 AND S92) OR S107 OR S108 OR S109 OR S110 OR S111 OR S112 OR S113 |  |
| S115 | S22 AND S114 |  |
| S116 | DE "Experimental Groups" |  |
| S117 | DE "Quasiexperimental Design" |  |
| S118 | DE "Control Groups" |  |
| S119 | DE "Matched Groups" |  |
| S120 | DE "Evaluation Research" |  |
| S121 | DE "Pretests Posttests" |  |
| S122 | DE "Pilot Projects" |  |
| S123 | DE "Comparative Analysis" |  |
| S124 | TI ( (randomis* or randomiz* or randomly or random allocat*) ) OR AB ( (randomis* or randomiz* or randomly or random allocat*) ) |  |
| S125 | AB (group# and (random* or between* or control* or intervent*)) |  |
| S126 | TI ( (multicenter or multi center or multicentre or multi centre) ) OR TI trial OR AB trial |  |
| S127 | TI ( (intervention* or controlled or control group or compare or comparison* or compared or ((prospectiv* or crossover) N5 (study or studies or design)) or (before N5 after) or (pre N5 post) or pretest or pre test or posttest or post test or quasiexperiment* or quasi experiment* or evaluat* or effect* or effectiveness or impact or time series or time point* or repeated measur*) ) OR AB ( (intervention* or controlled or control group or compare or comparison* or compared or ((prospectiv* or crossover) N5 (study or studies or design)) or (before N5 after) or (pre N5 post) or pretest or pre test or posttest or post test or quasiexperiment* or quasi experiment* or evaluat* or effect* or effectiveness or impact or time series or time point* or repeated measur*) ) |  |
| S128 | S116 OR S117 OR S118 OR S119 OR S120 OR S121 OR S122 OR S123 OR S124 OR S125 OR S126 OR S127 |  |
| S129 | S115 AND S128 |  |

| Teacher Reference Center (TRC) via EBSCOhost – 15.4.2016 | | |
| --- | --- | --- |
|  |  |  |
| **#** | **Query** |  |
| S1 | ZU "secondary schools" |  |
| S2 | ZU "middle schools" |  |
| S3 | ZU "high schools" |  |
| S4 | ZU "junior high schools" |  |
| S5 | ZU "special education schools" |  |
| S6 | ZU "elementary schools" |  |
| S7 | ZU "secondary education" |  |
| S8 | ZU "elementary education" |  |
| S9 | ZU "middle school students" |  |
| S10 | ZU "high school students" |  |
| S11 | ZU "secondary school students" |  |
| S12 | ZU "sixth grade (education)" |  |
| S13 | ZU "seventh grade (education)" |  |
| S14 | ZU "eighth grade (education)" |  |
| S15 | ZU "ninth grade (education)" |  |
| S16 | ZU "tenth grade (education)" |  |
| S17 | ZU "eleventh grade (education)" |  |
| S18 | ZU "twelfth grade (education)" |  |
| S19 | TI (schoolchild* or school-child* or school student* or pupil#) OR AB (schoolchild* or school-child* or school student* or pupil#) |  |
| S20 | TI ((middle or secondary or high ) N0 school#) OR AB ((middle or secondary or high) N0 school#) |  |
| S21 | TI ((six or sixth or seven* or eight# or nine or ninth or ten or tenth or eleven* or twelve or twelfth) N0 grade*) OR AB ((six or sixth or seven* or eight# or nine or ninth or ten or tenth or eleven* or twelve or twelfth) N0 grade*) |  |
| S22 | TI ( (grade# 6 or year 6 or grade# 7 or year 7 or grade# 8 or year 8 or grade# 9 or year 9 or grade# 10 or year 10 or grade# 11 or year 11 or grade# 12 or year 12) ) OR AB ( (grade# 6 or year 6 or grade# 7 or year 7 or grade# 8 or year 8 or grade# 9 or year 9 or grade# 10 or year 10 or grade# 11 or year 11 or grade# 12 or year 12) ) |  |
| S23 | TI (age# N2 ("11" or "12" or "13" or "14" or "15" or "16" or "17" or "18" or eleven or twelve or thirteen or fourteen or fifteen or sixteen or seventeen or eighteen)) OR AB (age# N2 ("11" or "12" or "13" or "14" or "15" or "16" or "17" or "18" or eleven or twelve or thirteen or fourteen or fifteen or sixteen or seventeen or eighteen)) |  |
| S24 | S1 OR S2 OR S3 OR S4 OR S5 OR S6 OR S7 OR S8 OR S9 OR S10 OR S11 OR S12 OR S13 OR S14 OR S15 OR S16 OR S17 OR S18 OR S19 OR S20 OR S21 OR S22 OR S23 |  |
| S25 | ZU "health education" |  |
| S26 | ZU "health education (middle school)" |  |
| S27 | ZU "health education (secondary)" |  |
| S28 | ZU "dental health education" |  |
| S29 | ZU "sex education" |  |
| S30 | ZU "health fairs" |  |
| S31 | ZU "physical education" |  |
| S32 | ZU "patient education" |  |
| S33 | ZU "home economics" |  |
| S34 | ZU "school health services" |  |
| S35 | ZU "evidence" |  |
| S36 | ZU "information services" |  |
| S37 | ZU "community information services" |  |
| S38 | ZU "information dissemination" |  |
| S39 | ZU "information resources" |  |
| S40 | ZU "information retrieval" |  |
| S41 | ZU "information science" |  |
| S42 | ZU "information-seeking behavior" |  |
| S43 | ZU "information-seeking strategies" |  |
| S44 | ZU "evaluation utilization" |  |
| S45 | ZU "research -- evaluation" |  |
| S46 | ZU "research -- methodology" |  |
| S47 | ZU "research use" |  |
| S48 | ZU "scientific experimentation" |  |
| S49 | ZU "scientific knowledge" |  |
| S50 | ZU "epidemiology" |  |
| S51 | ZU "medical research" |  |
| S52 | ZU "medical sciences" |  |
| S53 | ZU "science" |  |
| S54 | ZU "science classrooms & equipment" |  |
| S55 | ZU "science experiments" |  |
| S56 | ZU "science in literature" |  |
| S57 | ZU "science in mass media" |  |
| S58 | ZU "science journalism" |  |
| S59 | ZU "science on television" |  |
| S60 | ZU "science projects" |  |
| S61 | ZU "science students" |  |
| S62 | ZU "science television programs" |  |
| S63 | ZU "scientific apparatus & instruments" |  |
| S64 | ZU "scientific development" |  |
| S65 | ZU "scientific experimentation" |  |
| S66 | (ZU "statistics") |  |
| S67 | ZU "periodicals" |  |
| S68 | ZU "pamphlets" |  |
| S69 | ZU "electronic journals" |  |
| S70 | ZU "mass media" |  |
| S71 | ZU "newspapers" |  |
| S72 | ZU "newspapers in education" |  |
| S73 | ZU "newsletters" |  |
| S74 | ZU "radio" |  |
| S75 | ZU "educational television programs" |  |
| S76 | ZU "public television" |  |
| S77 | ZU "internet" |  |
| S78 | ZU "web 2.0" |  |
| S79 | ZU "reference sources" |  |
| S80 | ZU "web portals" |  |
| S81 | S25 OR S26 OR S27 OR S28 OR S29 OR S30 OR S31 OR S32 OR S33 OR S34 OR S35 OR S36 OR S37 OR S38 OR S39 OR S40 OR S41 OR S42 OR S43 OR S44 OR S45 OR S46 OR S47 OR S48 OR S49 OR S50 OR S51 OR S52 OR S53 OR S54 OR S55 OR S56 OR S57 OR S58 OR S59 OR S60 OR S61 OR S62 OR S63 OR S64 OR S65 OR S66 OR S67 OR S68 OR S69 OR S70 OR S71 OR S72 OR S73 OR S74 OR S75 OR S76 OR S77 OR S78 OR S79 OR S80 |  |
| S82 | ZU "health literacy" |  |
| S83 | ZU "health literacy -- research" |  |
| S84 | ZU "information literacy" |  |
| S85 | ZU "information literacy -- research" |  |
| S86 | ZU "information literacy -- study & teaching" |  |
| S87 | ZU "critical literacy" |  |
| S88 | ZU "judgment" |  |
| S89 | ZU "decision making" |  |
| S90 | ZU "problem solving" |  |
| S91 | ZU "problem-based learning" |  |
| S92 | ZU "critical thinking" |  |
| S93 | ZU "critical thinking -- study & teaching" |  |
| S94 | ZU "critical thinking -- research" |  |
| S95 | ZU "criticism" |  |
| S96 | ZU "information skills" |  |
| S97 | ZU "media literacy" |  |
| S98 | ZU "numeracy" |  |
| S99 | ZU "numeracy -- study & teaching" |  |
| S100 | ZU "scientific literacy" |  |
| S101 | ZU "problem solving -- research" |  |
| S102 | ZU "problem solving -- study & teaching" |  |
| S103 | ZU "decision making in adolescence" |  |
| S104 | ZU "decision making in children" |  |
| S105 | ZU "decision making in children" |  |
| S106 | ZU "decision making" |  |
| S107 | (ZU "critical thinking in adolescence") |  |
| S108 | (ZU "critical thinking in children") |  |
| S109 | (ZU "critical thinking in children -- study & teaching") |  |
| S110 | (ZU "critical analysis") |  |
| S111 | S82 OR S83 OR S84 OR S85 OR S86 OR S87 OR S88 OR S89 OR S90 OR S91 OR S92 OR S93 OR S94 OR S95 OR S96 OR S97 OR S98 OR S99 OR S100 OR S101 OR S102 OR S103 OR S104 OR S105 OR S106 OR S107 OR S108 OR S109 OR S110 |  |
| S112 | S81 AND S111 |  |
| S113 | TI ( ((health or information or mathematical or quantitative or science or scientific* or media) N2 (literacy or literate# or illiteracy or illiterate#)) ) OR AB ( ((health or information or mathematical or quantitative or science or scientific* or media) N2 (literacy or literate# or illiteracy or illiterate#)) ) |  |
| S114 | TI (information N2 competen*) OR AB (information N2 competen*) |  |
| S115 | TI numeracy OR AB numeracy |  |
| S116 | TI ( (scientific N2 (skill# or think* or reason*)) ) OR AB ( (scientific N2 (skill# or think* or reason*)) ) |  |
| S117 | TI ( (critical N2 (think* or reason*)) ) OR AB ( (critical N2 (think* or reason*)) ) |  |
| S118 | TI evidence-based OR AB evidence-based |  |
| S119 | S113 OR S114 OR S115 OR S116 OR S117 OR S118 |  |
| S120 | TI ( ((health or consumer or medical or scien*) N2 information) ) OR AB ( ((health or consumer or medical or scien*) N2 information) ) |  |
| S121 | TI risk information OR AB risk information |  |
| S122 | TI ( ((health or medical or scien*) N2 (claim# or statement# or message#)) ) OR AB ( ((health or medical or scien*) N2 (claim# or statement# or message#)) ) |  |
| S123 | TI ( ((health or medical or scien*) N2 (paper# or article# or report# or literature# or journal# or periodical# or research)) ) OR AB ( ((health or medical or scien*) N2 (paper# or article# or report# or literature# or journal# or periodical# or research)) ) |  |
| S124 | TI ( (media* or television* or tv or radio or broadcast* or broadsides or news* or maga?ine# or ((print* or written) N2 information) or pamphlet# or leaflet# or booklet# or brochure#) ) OR AB ( (media* or television* or tv or radio or broadcast* or broadsides or news* or maga?ine# or ((print* or written) N2 information) or pamphlet# or leaflet# or booklet# or brochure#) ) |  |
| S125 | TI ( (internet* or world wide web or worldwide web or web site# or website# or web portal# or blog* or web log* or bulletin board# or bulletinboard# or message board# or messageboard# or forum# or ehealth or e-health or electronic health or weblog* or moblog* or vlog* or video blog* or microblog* or wiki* or web page# or webpage# or chat room# or chatroom#) ) OR AB ( (internet* or world wide web or worldwide web or web site# or website# or web portal* or blog* or web log* or bulletin board# or bulletinboard# or message board# or messageboard# or forum# or ehealth or e-health or electronic health or weblog* or moblog* or vlog* or video blog* or microblog* or wiki* or web page# or webpage# or chat room# or chatroom#) ) |  |
| S126 | S120 OR S121 OR S122 OR S123 OR S124 OR S125 |  |
| S127 | S119 AND S126 |  |
| S128 | TI ( (((critical* N0 read*) or apprais* or evaluat* or understand* or assess* or judg* or interpret*) N7 ((health or consumer or medical or scien*) N2 information)) ) OR AB ( (((critical* N0 read*) or apprais* or evaluat* or understand* or assess* or judg* or interpret*) N7 ((health or consumer or medical or scien*) N2 information)) ) |  |
| S129 | TI ( (((critical* N0 read*) or apprais* or evaluat* or understand* or assess* or judg* or interpret*) N7 risk information) ) OR AB ( (((critical* N0 read*) or apprais* or evaluat* or understand* or assess* or judg* or interpret*) N7 risk information) ) |  |
| S130 | TI ( (((critical* N0 read*) or apprais* or evaluat* or understand* or assess* or judg* or interpret*) N7 ((health or medical or scien*) N2 (claim# or statement# or message#))) ) OR AB ( (((critical* N0 read*) or apprais* or evaluat* or understand* or assess* or judg* or interpret*) N7 ((health or medical or scien*) N2 (claim# or statement# or message#))) ) |  |
| S131 | TI ( (((critical* N0 read*) or apprais* or evaluat* or understand* or assess* or judg* or interpret*) N7 ((health or medical or scien*) N2 (paper# or article# or report# or literature# or journal# or periodical# or research))) ) OR AB ( (((critical* N0 read*) or apprais* or evaluat* or understand* or assess* or judg* or interpret*) N7 ((health or medical or scien*) N2 (paper# or article# or report# or literature# or journal# or periodical# or research))) ) |  |
| S132 | TI ( (((critical* N0 read*) or apprais* or evaluat* or understand* or assess* or judg* or interpret*) N7 (media* or television* or tv or radio or broadcast* or broadsides or news* or maga?ine# or ((print* or written) N2 information) or pamphlet# or leaflet# or booklet# or brochure#)) ) OR AB ( (((critical* N0 read*) or apprais* or evaluat* or understand* or assess* or judg* or interpret*) N10 (media* or television* or tv or radio or broadcast* or broadsides or news* or maga?ine# or ((print* or written) N2 information) or pamphlet# or leaflet# or booklet# or brochure#)) ) |  |
| S133 | TI ( (((critical* N0 read*) or apprais* or evaluat* or understand* or assess* or judg* or interpret*) N7 (internet* or world wide web or worldwide web or web site# or website# or web portal# or blog* or web log* or bulletin board# or bulletinboard# or message board# or messageboard# or forum# or ehealth or e-health or electronic health or weblog* or moblog* or vlog* or video blog* or microblog* or wiki* or web page# or webpage# or chat room# or chatroom#)) ) OR AB ( (((critical* N0 read*) or apprais* or evaluat* or understand* or assess* or judg* or interpret*) N7 (internet* or world wide web or worldwide web or web site# or website# or web portal# or blog* or web log* or bulletin board# or bulletinboard# or message board# or messageboard# or forum# or ehealth or e-health or electronic health or weblog* or moblog* or vlog* or video blog* or microblog* or wiki* or web page# or webpage# or chat room# or chatroom#)) ) |  |
| S134 | S112 OR S127 OR S128 OR S129 OR S130 OR S131 OR S132 OR S133 |  |
| S135 | S24 AND S134 |  |
| S136 | ZU "randomized controlled trials" |  |
| S137 | ZU "clinical trials" |  |
| S138 | ZU "pre-tests & post-tests" |  |
| S139 | ZU "crossover trials" |  |
| S140 | ZU "time series analysis" |  |
| S141 | ZU "pilot projects" |  |
| S142 | ZU "repeated measures design" |  |
| S143 | TI ( (randomis* or randomiz* or randomly or random allocat*) ) OR AB ( (randomis* or randomiz* or randomly or random allocat*) ) |  |
| S144 | AB (group? and (random* or between* or control* or intervent*)) |  |
| S145 | TI ( (multicenter or multi center or multicentre or multi centre) ) OR TI trial OR AB trial |  |
| S146 | TI ( (intervention* or controlled or control group or compare or comparison* or compared or ((prospectiv* or crossover) N5 (study or studies or design)) or (before N5 after) or (pre N5 post) or pretest or pre test or posttest or post test or quasiexperiment* or quasi experiment* or evaluat* or effect* or effectiveness or impact or time series or time point* or repeated measur*) ) OR AB ( (intervention* or controlled or control group or compare or comparison* or compared or ((prospectiv* or crossover) N5 (study or studies or design)) or (before N5 after) or (pre N5 post) or pretest or pre test or posttest or post test or quasiexperiment* or quasi experiment* or evaluat* or effect* or effectiveness or impact or time series or time point* or repeated measur*) ) |  |
| S147 | S136 OR S137 OR S138 OR S139 OR S140 OR S141 OR S142 OR S143 OR S144 OR S145 OR S146 |  |
| S148 | S135 AND S147 |  |

|  | | |
| --- | --- | --- |
| Library, Information Science & Technology Abstracts (LISTA) via EBSCOhost – 15.4.2016 | | |
| **#** | **Query** |  |
| S1 | TI ( (age# N2 (11 or 12 or 13 or 14 or 15 or 16 or 17 or 18 or eleven or twelve or thirteen or fourteen or fifteen or sixteen or seventeen or eighteen)) ) OR AB ( (age# N2 (11 or 12 or 13 or 14 or 15 or 16 or 17 or 18 or eleven or twelve or thirteen or fourteen or fifteen or sixteen or seventeen or eighteen)) ) |  |
| S2 | DE "SCHOOLS" |  |
| S3 | DE "STUDENTS" |  |
| S4 | TI ( (schoolchild* or school-child* or school student* or pupil#) ) OR AB ( (schoolchild* or school-child* or school student* or pupil#) ) |  |
| S5 | TI ( ((middle or secondary or high) N0 school#) ) OR AB ( ((middle or secondary or high) N0 school#) ) |  |
| S6 | TI ( ((six or sixth or seven* or eight# or nine or ninth or ten or tenth or eleven* or twelve or twelfth) N3 grade*) ) OR AB ( ((six or sixth or seven* or eight# or nine or ninth or ten or tenth or eleven* or twelve or twelfth) N3 grade*) ) |  |
| S7 | TI ( (grade# 6 or grade# 7 or grade# 8 or grade# 9 or grade# 10 or grade# 11 or grade# 12) ) OR AB ( (grade# 6 or grade# 7 or grade# 8 or grade# 9 or grade# 10 or grade# 11 or grade# 12) ) OR TI ( (year# 6 or year# 7 or year# 8 or year# 9 or year# 10 or year# 11 or year# 12 or year# 13) ) OR AB ( (year# 6 or year# 7 or year# 8 or year# 9 or year# 10 or year# 11 or year# 12 or year# 13) ) |  |
| S8 | S2 OR S3 OR S4 OR S5 OR S6 OR S7 |  |
| S9 | DE "EDUCATION" |  |
| S10 | DE "CURRICULA (Courses of study)" |  |
| S11 | DE "DISTANCE education" |  |
| S12 | DE "LITERACY education" |  |
| S13 | DE "USER education" |  |
| S14 | DE "COMPUTER assisted instruction" |  |
| S15 | DE "EDUCATIONAL technology" |  |
| S16 | DE "AUDIOVISUAL education" |  |
| S17 | TI ( (educat* or train* or teach* or workshop# or work-shop# or seminar# or course# or curricul* or learn* or instruct* or self-instruct* or selfinstruct* or coach* or skill* or problem-based or pedagog* or class or classes or lesson# or taught or module#) ) OR AB ( (educat* or train* or teach* or workshop# or work-shop# or seminar# or course# or curricul* or learn* or instruct* or self-instruct* or selfinstruct* or coach* or skill* or problem-based or pedagog* or class or classes or lesson# or taught or module#) ) |  |
| S18 | S9 OR S10 OR S11 OR S12 OR S13 OR S14 OR S15 OR S16 OR S17 |  |
| S19 | DE "INFORMATION services" |  |
| S20 | DE "RESEARCH" |  |
| S21 | DE "COMPUTER assisted research" |  |
| S22 | DE "EXPERIMENTAL design" |  |
| S23 | DE "EXPERIMENTS" |  |
| S24 | DE "INFORMATION science -- Research" |  |
| S25 | DE "INFORMATION resources -- Research" |  |
| S26 | DE "INFORMATION resources -- Use studies" |  |
| S27 | DE "INTERNET research" |  |
| S28 | DE "OPERATIONS research" |  |
| S29 | DE "QUALITATIVE research" |  |
| S30 | DE "QUANTITATIVE research" |  |
| S31 | DE "NEWSPAPERS" |  |
| S32 | DE "SERIAL publications" |  |
| S33 | DE "ELECTRONIC newspapers" |  |
| S34 | DE "PERIODICALS" |  |
| S35 | DE "PAMPHLETS" |  |
| S36 | DE "BROCHURES" |  |
| S37 | DE "DIGITAL media" |  |
| S38 | DE "INTERNET" |  |
| S39 | DE "INTERNET in education" |  |
| S40 | DE "VIRTUAL communities" |  |
| S41 | DE "MASS media" |  |
| S42 | DE "WEB 2.0" |  |
| S43 | DE "BLOGS" |  |
| S44 | DE "WIKIS (Computer science)" |  |
| S45 | DE "AUDIOVISUAL materials" |  |
| S46 | S19 OR S20 OR S21 OR S22 OR S23 OR S24 OR S25 OR S26 OR S27 OR S28 OR S29 OR S30 OR S31 OR S32 OR S33 OR S34 OR S35 OR S36 OR S37 OR S38 OR S39 OR S40 OR S41 OR S42 OR S43 OR S44 OR S45 |  |
| S47 | DE "PROBLEM solving" |  |
| S48 | DE "SEARCHING behavior" |  |
| S49 | S47 OR S48 |  |
| S50 | S46 AND S49 |  |
| S51 | DE "ELECTRONIC information resource literacy" |  |
| S52 | DE "HEALTH literacy" |  |
| S53 | DE "MEDIA literacy" |  |
| S54 | DE "INTERNET literacy" |  |
| S55 | DE "LITERACY" |  |
| S56 | DE "COMPUTER literacy" |  |
| S57 | DE "INFORMATION literacy" |  |
| S58 | DE "VISUAL literacy" |  |
| S59 | S50 OR S51 OR S52 OR S53 OR S54 OR S55 OR S56 OR S57 OR S58 |  |
| S60 | TI ( ((health or information or mathematical or quantitative or science or scientific* or media) N2 (literacy or literate# or illiteracy or illiterate#)) ) OR AB ( ((health or information or mathematical or quantitative or science or scientific* or media) N2 (literacy or literate# or illiteracy or illiterate#)) ) |  |
| S61 | TI (information N2 competen*) OR AB (information N2 competen*) |  |
| S62 | TI numeracy OR AB numeracy |  |
| S63 | TI ( (scientific N2 (skill# or think* or reason*)) ) OR AB ( (scientific N2 (skill# or think* or reason*)) ) |  |
| S64 | TI ( (critical N2 (think* or reason*)) ) OR AB ( (critical N2 (think* or reason*)) ) |  |
| S65 | TI evidence-based OR AB evidence-based |  |
| S66 | S60 OR S61 OR S62 OR S63 OR S64 OR S65 |  |
| S67 | TI ( ((health or consumer or medical or scien*) N2 information) ) OR AB ( ((health or consumer or medical or scien*) N2 information) ) |  |
| S68 | TI risk information OR AB risk information |  |
| S69 | TI ( ((health or medical or scien*) N2 (claim# or statement# or message#)) ) OR AB ( ((health or medical or scien*) N2 (claim# or statement# or message#)) ) |  |
| S70 | TI ( ((health or medical or scien*) N2 (paper# or article# or report# or literature# or journal# or periodical# or research)) ) OR AB ( ((health or medical or scien*) N2 (paper# or article# or report# or literature# or journal# or periodical# or research)) ) |  |
| S71 | TI ( (media* or television* or tv or radio or broadcast* or broadsides or news* or maga?ine# or ((print* or written) N2 information) or pamphlet# or leaflet# or booklet# or brochure#) ) OR AB ( (media* or television* or tv or radio or broadcast* or broadsides or news* or maga?ine# or ((print* or written) N2 information) or pamphlet# or leaflet# or booklet# or brochure#) ) |  |
| S72 | TI ( (internet* or world wide web or worldwide web or web site# or website# or web portal# or blog* or web log* or bulletin board# or bulletinboard# or message board# or messageboard# or forum# or ehealth or e-health or electronic health or weblog* or moblog* or vlog* or video blog* or microblog* or wiki* or web page# or webpage# or chat room# or chatroom#) ) OR AB ( (internet* or world wide web or worldwide web or web site# or website# or web portal* or blog* or web log* or bulletin board# or bulletinboard# or message board# or messageboard# or forum# or ehealth or e-health or electronic health or weblog* or moblog* or vlog* or video blog* or microblog* or wiki* or web page# or webpage# or chat room# or chatroom#) ) |  |
| S73 | S67 OR S68 OR S69 OR S70 OR S71 OR S72 |  |
| S74 | S66 AND S73 |  |
| S75 | TI ( (((critical* N0 read*) or apprais* or evaluat* or understand* or assess* or judg* or interpret*) N7 ((health or consumer or medical or scien*) N2 information)) ) OR AB ( (((critical* N0 read*) or apprais* or evaluat* or understand* or assess* or judg* or interpret*) N7 ((health or consumer or medical or scien*) N2 information)) ) |  |
| S76 | TI ( (((critical* N0 read*) or apprais* or evaluat* or understand* or assess* or judg* or interpret*) N7 risk information) ) OR AB ( (((critical* N0 read*) or apprais* or evaluat* or understand* or assess* or judg* or interpret*) N7 risk information) ) |  |
| S77 | TI ( (((critical* N0 read*) or apprais* or evaluat* or understand* or assess* or judg* or interpret*) N7 ((health or medical or scien*) N2 (claim# or statement# or message#))) ) OR AB ( (((critical* N0 read*) or apprais* or evaluat* or understand* or assess* or judg* or interpret*) N7 ((health or medical or scien*) N2 (claim# or statement# or message#))) ) |  |
| S78 | TI ( (((critical* N0 read*) or apprais* or evaluat* or understand* or assess* or judg* or interpret*) N7 ((health or medical or scien*) N2 (paper# or article# or report# or literature# or journal# or periodical# or research))) ) OR AB ( (((critical* N0 read*) or apprais* or evaluat* or understand* or assess* or judg* or interpret*) N7 ((health or medical or scien*) N2 (paper# or article# or report# or literature# or journal# or periodical# or research))) ) |  |
| S79 | TI ( (((critical* N0 read*) or apprais* or evaluat* or understand* or assess* or judg* or interpret*) N7 (media* or television* or tv or radio or broadcast* or broadsides or news* or maga?ine# or ((print* or written) N2 information) or pamphlet# or leaflet# or booklet# or brochure#)) ) OR AB ( (((critical* N0 read*) or apprais* or evaluat* or understand* or assess* or judg* or interpret*) N10 (media* or television* or tv or radio or broadcast* or broadsides or news* or maga?ine# or ((print* or written) N2 information) or pamphlet# or leaflet# or booklet# or brochure#)) ) |  |
| S80 | TI ( (((critical* N0 read*) or apprais* or evaluat* or understand* or assess* or judg* or interpret*) N7 (internet* or world wide web or worldwide web or web site# or website# or web portal# or blog* or web log* or bulletin board# or bulletinboard# or message board# or messageboard# or forum# or ehealth or e-health or electronic health or weblog* or moblog* or vlog* or video blog* or microblog* or wiki* or web page# or webpage# or chat room# or chatroom#)) ) OR AB ( (((critical* N0 read*) or apprais* or evaluat* or understand* or assess* or judg* or interpret*) N7 (internet* or world wide web or worldwide web or web site# or website# or web portal# or blog* or web log* or bulletin board# or bulletinboard# or message board# or messageboard# or forum# or ehealth or e-health or electronic health or weblog* or moblog* or vlog* or video blog* or microblog* or wiki* or web page# or webpage# or chat room# or chatroom#)) ) |  |
| S81 | S59 OR S74 OR S75 OR S76 OR S77 OR S78 OR S79 OR S80 |  |
| S82 | S1 AND S18 AND S81 |  |
| S83 | S8 AND S81 |  |
| S84 | DE "EXPERIMENTAL design" |  |
| S85 | TI ( (randomis* or randomiz* or randomly or random allocat*) ) OR AB ( (randomis* or randomiz* or randomly or random allocat*) ) |  |
| S86 | AB (group? and (random* or between* or control* or intervent*)) |  |
| S87 | TI ( (multicenter or multi center or multicentre or multi centre) ) OR TI trial OR AB trial |  |
| S88 | TI ( (intervention* or controlled or control group or compare or comparison* or compared or ((prospectiv* or crossover) N5 (study or studies or design)) or (before N5 after) or (pre N5 post) or pretest or pre test or posttest or post test or quasiexperiment* or quasi experiment* or evaluat* or effect* or effectiveness or impact or time series or time point* or repeated measur*) ) OR AB ( (intervention* or controlled or control group or compare or comparison* or compared or ((prospectiv* or crossover) N5 (study or studies or design)) or (before N5 after) or (pre N5 post) or pretest or pre test or posttest or post test or quasiexperiment* or quasi experiment* or evaluat* or effect* or effectiveness or impact or time series or time point* or repeated measur*) ) |  |
| S89 | S84 OR S85 OR S86 OR S87 OR S88 |  |
| S90 | S82 AND S89 |  |
| S91 | S83 AND S89 |  |
| S92 | S90 OR S91 |  |

|  | | |
| --- | --- | --- |
| Cumulative Index to Nursing and Allied Health Literature (Cinahl) via EBSCOhost – 15.4.2016 | | |
|  | | |
| **#** | **Query** |  |
| S1 | TI ( (age# N2 (11 or 12 or 13 or 14 or 15 or 16 or 17 or 18 or eleven or twelve or thirteen or fourteen or fifteen or sixteen or seventeen or eighteen)) ) OR AB ( (age# N2 (11 or 12 or 13 or 14 or 15 or 16 or 17 or 18 or eleven or twelve or thirteen or fourteen or fifteen or sixteen or seventeen or eighteen)) ) |  |
| S2 | (MH "Schools") |  |
| S3 | (MH "Schools, Middle") |  |
| S4 | (MH "Schools, Secondary") |  |
| S5 | (MH "Schools, Special") |  |
| S6 | (MH "School Health Services") |  |
| S7 | (MH "Students") |  |
| S8 | (MH "Students, High School") |  |
| S9 | (MH "Students, Middle School") |  |
| S10 | TI ( (schoolchild* or school-child* or school student* or pupil#) ) OR AB ( (schoolchild* or school-child* or school student* or pupil#) ) |  |
| S11 | TI ( ((middle or secondary or high) N0 school#) ) OR AB ( ((middle or secondary or high) N0 school#) ) |  |
| S12 | TI ( ((six or sixth or seven* or eight# or nine or ninth or ten or tenth or eleven* or twelve or twelfth) N3 grade*) ) OR AB ( ((six or sixth or seven* or eight# or nine or ninth or ten or tenth or eleven* or twelve or twelfth) N3 grade*) ) |  |
| S13 | TI ( (grade# 6 or grade# 7 or grade# 8 or grade# 9 or grade# 10 or grade# 11 or grade# 12) ) OR AB ( (grade# 6 or grade# 7 or grade# 8 or grade# 9 or grade# 10 or grade# 11 or grade# 12) ) OR TI ( (year# 6 or year# 7 or year# 8 or year# 9 or year# 10 or year# 11 or year# 12 or year# 13) ) OR AB ( (year# 6 or year# 7 or year# 8 or year# 9 or year# 10 or year# 11 or year# 12 or year# 13) ) |  |
| S14 | S2 OR S3 OR S4 OR S5 OR S6 OR S7 OR S8 OR S9 OR S10 OR S11 OR S12 OR S13 |  |
| S15 | (MH "Education") |  |
| S16 | (MH "Health Education") |  |
| S17 | (MH "School Health Education") |  |
| S18 | (MH "Dental Health Education") |  |
| S19 | (MH "Health Fairs") |  |
| S20 | (MH "Sex Education") |  |
| S21 | (MH "Nutrition Education") |  |
| S22 | (MH "Patient Education") |  |
| S23 | (MH "Education, Non-Traditional") |  |
| S24 | (MH "Education, Special") |  |
| S25 | (MH "Education, Nonprofessional") |  |
| S26 | (MH "Education, Competency-Based") |  |
| S27 | (MH "Curriculum+") |  |
| S28 | (MH "Programmed Instruction+") |  |
| S29 | (MH "Teaching") |  |
| S30 | (MH "Teaching Materials+") |  |
| S31 | (MH "Educational Technology") |  |
| S32 | TI ( (educat* or train* or teach* or workshop# or work-shop# or seminar# or course# or curricul* or learn* or instruct* or self-instruct* or selfinstruct* or coach* or skill* or problem-based or pedagog* or class or classes or lesson# or taught or module#) ) OR AB ( (educat* or train* or teach* or workshop# or work-shop# or seminar# or course# or curricul* or learn* or instruct* or self-instruct* or selfinstruct* or coach* or skill* or problem-based or pedagog* or class or classes or lesson# or taught or module#) ) |  |
| S33 | S15 OR S16 OR S17 OR S18 OR S19 OR S20 OR S21 OR S22 OR S23 OR S24 OR S25 OR S26 OR S27 OR S28 OR S29 OR S30 OR S31 OR S32 |  |
| S34 | (MH "Health Information+") |  |
| S35 | (MH "Information Services") |  |
| S36 | (MH "Research") |  |
| S37 | (MH "Empirical Research") |  |
| S38 | (MH "Study Design") |  |
| S39 | (MH "Statistics") |  |
| S40 | (MH "Epidemiology") |  |
| S41 | (MH "Newspapers") |  |
| S42 | (MH "Pamphlets") |  |
| S43 | (MH "Television") |  |
| S44 | (MH "Radio") |  |
| S45 | (MH "Internet+") |  |
| S46 | (MH "Social Media") |  |
| S47 | S34 OR S35 OR S36 OR S37 OR S38 OR S39 OR S40 OR S41 OR S42 OR S43 OR S44 OR S45 OR S46 |  |
| S48 | (MH "Information Seeking Behavior") |  |
| S49 | (MH "Problem Solving") |  |
| S50 | (MH "Problem-Based Learning") |  |
| S51 | (MH "Professional Practice, Evidence-Based") |  |
| S52 | S48 OR S49 OR S50 OR S51 |  |
| S53 | S47 AND S52 |  |
| S54 | (MH "Science") or (MH "Information Literacy") or (MH "Decision Making") or (MH "Critical Thinking") or (MH "Judgment") or (MH "Thinking") |  |
| S55 | S53 OR S54 |  |
| S56 | TI ( ((health or information or mathematical or quantitative or science or scientific* or media) N2 (literacy or literate# or illiteracy or illiterate#)) ) OR AB ( ((health or information or mathematical or quantitative or science or scientific* or media) N2 (literacy or literate# or illiteracy or illiterate#)) ) |  |
| S57 | TI (information N2 competen*) OR AB (information N2 competen*) |  |
| S58 | TI numeracy OR AB numeracy |  |
| S59 | TI ( (scientific N2 (skill# or think* or reason*)) ) OR AB ( (scientific N2 (skill# or think* or reason*)) ) |  |
| S60 | TI ( (critical N2 (think* or reason*)) ) OR AB ( (critical N2 (think* or reason*)) ) |  |
| S61 | TI evidence-based OR AB evidence-based |  |
| S62 | S56 OR S57 OR S58 OR S59 OR S60 OR S61 |  |
| S63 | TI ( ((health or consumer or medical or scien*) N2 information) ) OR AB ( ((health or consumer or medical or scien*) N2 information) ) |  |
| S64 | TI risk information OR AB risk information |  |
| S65 | TI ( ((health or medical or scien*) N2 (claim# or statement# or message#)) ) OR AB ( ((health or medical or scien*) N2 (claim# or statement# or message#)) ) |  |
| S66 | TI ( ((health or medical or scien*) N2 (paper# or article# or report# or literature# or journal# or periodical# or research)) ) OR AB ( ((health or medical or scien*) N2 (paper# or article# or report# or literature# or journal# or periodical# or research)) ) |  |
| S67 | TI ( (media* or television* or tv or radio or broadcast* or broadsides or news* or maga?ine# or ((print* or written) N2 information) or pamphlet# or leaflet# or booklet# or brochure#) ) OR AB ( (media* or television* or tv or radio or broadcast* or broadsides or news* or maga?ine# or ((print* or written) N2 information) or pamphlet# or leaflet# or booklet# or brochure#) ) |  |
| S68 | TI ( (internet* or world wide web or worldwide web or web site# or website# or web portal# or blog* or web log* or bulletin board# or bulletinboard# or message board# or messageboard# or forum# or ehealth or e-health or electronic health or weblog* or moblog* or vlog* or video blog* or microblog* or wiki* or web page# or webpage# or chat room# or chatroom#) ) OR AB ( (internet* or world wide web or worldwide web or web site# or website# or web portal* or blog* or web log* or bulletin board# or bulletinboard# or message board# or messageboard# or forum# or ehealth or e-health or electronic health or weblog* or moblog* or vlog* or video blog* or microblog* or wiki* or web page# or webpage# or chat room# or chatroom#) ) |  |
| S69 | S63 OR S64 OR S65 OR S66 OR S67 OR S68 |  |
| S70 | S62 AND S69 |  |
| S71 | TI ( (((critical* N0 read*) or apprais* or evaluat* or understand* or assess* or judg* or interpret*) N7 ((health or consumer or medical or scien*) N2 information)) ) OR AB ( (((critical* N0 read*) or apprais* or evaluat* or understand* or assess* or judg* or interpret*) N7 ((health or consumer or medical or scien*) N2 information)) ) |  |
| S72 | TI ( (((critical* N0 read*) or apprais* or evaluat* or understand* or assess* or judg* or interpret*) N7 risk information) ) OR AB ( (((critical* N0 read*) or apprais* or evaluat* or understand* or assess* or judg* or interpret*) N7 risk information) ) |  |
| S73 | TI ( (((critical* N0 read*) or apprais* or evaluat* or understand* or assess* or judg* or interpret*) N7 ((health or medical or scien*) N2 (claim# or statement# or message#))) ) OR AB ( (((critical* N0 read*) or apprais* or evaluat* or understand* or assess* or judg* or interpret*) N7 ((health or medical or scien*) N2 (claim# or statement# or message#))) ) |  |
| S74 | TI ( (((critical* N0 read*) or apprais* or evaluat* or understand* or assess* or judg* or interpret*) N7 ((health or medical or scien*) N2 (paper# or article# or report# or literature# or journal# or periodical# or research))) ) OR AB ( (((critical* N0 read*) or apprais* or evaluat* or understand* or assess* or judg* or interpret*) N7 ((health or medical or scien*) N2 (paper# or article# or report# or literature# or journal# or periodical# or research))) ) |  |
| S75 | TI ( (((critical* N0 read*) or apprais* or evaluat* or understand* or assess* or judg* or interpret*) N7 (media* or television* or tv or radio or broadcast* or broadsides or news* or maga?ine# or ((print* or written) N2 information) or pamphlet# or leaflet# or booklet# or brochure#)) ) OR AB ( (((critical* N0 read*) or apprais* or evaluat* or understand* or assess* or judg* or interpret*) N10 (media* or television* or tv or radio or broadcast* or broadsides or news* or maga?ine# or ((print* or written) N2 information) or pamphlet# or leaflet# or booklet# or brochure#)) ) |  |
| S76 | TI ( (((critical* N0 read*) or apprais* or evaluat* or understand* or assess* or judg* or interpret*) N7 (internet* or world wide web or worldwide web or web site# or website# or web portal# or blog* or web log* or bulletin board# or bulletinboard# or message board# or messageboard# or forum# or ehealth or e-health or electronic health or weblog* or moblog* or vlog* or video blog* or microblog* or wiki* or web page# or webpage# or chat room# or chatroom#)) ) OR AB ( (((critical* N0 read*) or apprais* or evaluat* or understand* or assess* or judg* or interpret*) N7 (internet* or world wide web or worldwide web or web site# or website# or web portal# or blog* or web log* or bulletin board# or bulletinboard# or message board# or messageboard# or forum# or ehealth or e-health or electronic health or weblog* or moblog* or vlog* or video blog* or microblog* or wiki* or web page# or webpage# or chat room# or chatroom#)) ) |  |
| S77 | S55 OR S70 OR S71 OR S72 OR S73 OR S74 OR S75 OR S76 |  |
| S78 | S1 AND S33 AND S77 |  |
| S79 | S14 AND S77 |  |
| S80 | S78 OR S79 |  |
| S81 | (MH "Experimental Studies+") |  |
| S82 | (MH "Crossover Design") |  |
| S83 | (MH "Repeated Measures") |  |
| S84 | (MH "Quasi-Experimental Studies+") |  |
| S85 | (MH "Multicenter Studies") |  |
| S86 | (MH "Pilot Studies") |  |
| S87 | (MH "Program Evaluation") |  |
| S88 | TI ( (randomis* or randomiz* or randomly or random allocat*) ) OR AB ( (randomis* or randomiz* or randomly or random allocat*) ) |  |
| S89 | AB (group? and (random* or between* or control* or intervent*)) |  |
| S90 | TI ( (multicenter or multi center or multicentre or multi centre) ) OR TI trial OR AB trial |  |
| S91 | TI ( (intervention* or controlled or control group or compare or comparison* or compared or ((prospectiv* or crossover) N5 (study or studies or design)) or (before N5 after) or (pre N5 post) or pretest or pre test or posttest or post test or quasiexperiment* or quasi experiment* or evaluat* or effect* or effectiveness or impact or time series or time point* or repeated measur*) ) OR AB ( (intervention* or controlled or control group or compare or comparison* or compared or ((prospectiv* or crossover) N5 (study or studies or design)) or (before N5 after) or (pre N5 post) or pretest or pre test or posttest or post test or quasiexperiment* or quasi experiment* or evaluat* or effect* or effectiveness or impact or time series or time point* or repeated measur*) ) |  |
| S92 | S81 OR S82 OR S83 OR S84 OR S85 OR S86 OR S87 OR S88 OR S89 OR S90 OR S91 |  |
| S93 | S80 AND S92 |  |

| Science Citation Index Expanded and Social Sciences Citation Index – 15.4.2016 | |
| --- | --- |
| **#** | **Query** |
| # 39 | #38 AND #37 |
| # 38 | PY=(2014 or 2015) |
| # 37 | #36 AND #32 |
| # 36 | #35 OR #34 OR #33 |
| # 35 | TS=(intervention* or controlled or (control NEAR/0 Group) or compare or comparison* or compared or ((prospectiv* or crossover) NEAR/5 (study or studies or design)) or (before NEAR/5 after) or (pre NEAR/5 post) or pretest or (pre NEAR/0 test) or posttest or (post NEAR/0 test) or quasiexperiment* or (quasi NEAR/0 experiment*) or evaluat* or effect$ or effectiveness or impact or (time NEAR/0 series) or (time NEAR/0 point$) or (repeated NEAR/0 measur*)) |
| # 34 | TI=(multicenter or (multi NEAR/0 center) or multicentre or (multi NEAR/0 centre)) OR TS=trial |
| # 33 | TS=(randomis* or randomiz* or randomly or random allocat*) OR TS=(Group$ and (random* or between* or control* or intervent*)) |
| # 32 | #31 OR #30 |
| # 31 | #29 AND #6 |
| # 30 | #29 AND #7 AND #1 |
| # 29 | #28 OR #27 OR #26 OR #25 OR #24 OR #23 OR #22 |
| # 28 | TS=(((critical* NEAR/0 read*) or apprais* or evaluat* or understand* or assess* or judg* or interpret*) NEAR/7 (internet* or (world NEAR/0 wide NEAR/0 web) or (worldwide NEAR/0 web) or (web NEAR/0 site$) or website$ or (web NEAR/0 portal$) or blog* or (web NEAR/0 log*) or (bulletin NEAR/0 board$) or bulletinboard$ or (message NEAR/0 board$) or messageboard$ or forum$ or ehealth or e-health or (electronic NEAR/0 health) or weblog* or moblog* or vlog* or (video NEAR/0 blog*) or microblog* or wiki* or (web NEAR/0 page$) or webpage$ or (chat NEAR/0 room$) or chatroom$)) |
| # 27 | TS=(((critical* NEAR/0 read*) or apprais* or evaluat* or understand* or assess* or judg* or interpret*) NEAR/7 (media* or television* or tv or radio or broadcast* or broadsides or news* or maga?ine$ or ((print* or written) NEAR/2 information) or pamphlet$ or leaflet$ or booklet$ or brochure$)) |
| # 26 | TS=(((critical* NEAR/0 read*) or apprais* or evaluat* or understand* or assess* or judg* or interpret*) NEAR/7 ((health or medical or scien*) NEAR/2 (paper$ or article$ or report$ or literature$ or journal$ or periodical$ or research))) |
| # 25 | TS=(((critical* NEAR/0 read*) or apprais* or evaluat* or understand* or assess* or judg* or interpret*) NEAR/7 ((health or medical or scien*) NEAR/2 (claim$ or statement$ or message$))) |
| # 24 | TS=(((critical* NEAR/0 read*) or apprais* or evaluat* or understand* or assess* or judg* or interpret*) NEAR/7 (risk NEAR/0 information)) |
| # 23 | TS=((((critical* NEAR/0 read*) or apprais* or evaluat* or understand* or assess* or judg* or interpret*) NEAR/7 ((health or consumer or medical or scien*) NEAR/2 information))) |
| # 22 | #21 AND #14 |
| # 21 | #20 OR #19 OR #18 OR #17 OR #16 OR #15 |
| # 20 | TS=((internet* or (world NEAR/0 wide NEAR/0 web) or (worldwide NEAR/0 web) or (web NEAR/0 site$) or website$ or (web NEAR/0 portal$) or blog* or (web NEAR/0 log*) or (bulletin NEAR/0 board$ or bulletinboard$ or (message NEAR/0 board$) or messageboard$ or forum$ or ehealth or e-health or (electronic NEAR/0 health) or weblog* or moblog* or vlog* or (video NEAR/0 blog*) or microblog* or wiki* or (web NEAR/0 page$) or webpage$ or (chat NEAR/0 room$) or chatroom$) )) |
| # 19 | TS=((media* or television* or tv or radio or broadcast* or broadsides or news* or magazine$ or ((print* or written) NEAR/2 information) or pamphlet$ or leaflet$ or booklet$ or brochure$)) |
| # 18 | TS= (((health or medical or scien*) NEAR/2 (paper$ or article$ or report$ or literature$ or journal$ or periodical$ or research))) |
| # 17 | TS= (((health or medical or scien*) NEAR/2 (claim$ or statement$ or message$))) |
| # 16 | TS= (risk NEAR/0 information) |
| # 15 | TS= (((health or consumer or medical or scien*) NEAR/2 information)) |
| # 14 | #13 OR #12 OR #11 OR #10 OR #9 OR #8 |
| # 13 | TS= (evidence-based) |
| # 12 | TS= ((critical NEAR/2 (think* or reason*))) |
| # 11 | TS= ((scientific NEAR/2 (skill$ or think* or reason*))) |
| # 10 | TS= (numeracy) |
| # 9 | TS=((information NEAR/2 competen*)) |
| # 8 | TS=(((health or information or mathematical or quantitative or science or scientific* or media) NEAR/2 (literacy or literate$ or illiteracy or illiterate$))) |
| # 7 | TS=((educat* or train* or teach* or workshop$ or work-shop$ or seminar$ or course$ or curricul* or learn* or instruct* or self-instruct* or selfinstruct* or coach* or skill* or problem-based or pedagog* or class or classes or lesson$ or taught or module$)) |
| # 6 | #5 OR #4 OR #3 OR #2 |
| # 5 | (TS=((grade$ NEAR/2 6) or (grade$ NEAR/2 7) or (grade$ NEAR/2 8) or (grade$ NEAR/2 9) or (grade$ NEAR/2 10) or (grade$ NEAR/2 11) or (grade$ NEAR/2 12))) OR (TS=("year$ 6" or "year$ 7" or "year$ 8" or "year$ 9" or "year$ 10" or "year$ 11" or "year$ 12" or "year$ 13")) |
| # 4 | TS=(((six or sixth or seven* or eight$ or nine or ninth or ten or tenth or eleven* or twelve or twelfth) NEAR/3 grade*)) |
| # 3 | TS=(((middle or secondary or high) NEAR/0 School$)) |
| # 2 | TS=((schoolchild* or school-child* or (school NEAR/2 student*) or pupil$)) |
| # 1 | TS=((age$ NEAR/2 (11 or 12 or 13 or 14 or 15 or 16 or 17 or 18 or eleven or twelve or thirteen or fourteen or fifteen or sixteen or seventeen or eighteen))) |

| Sociological Abstracts (ProQuest) – 15.4.2016 |
| --- |
|  |
| (((SU.EXACT("Longitudinal Studies") OR SU.EXACT("Research Design") OR SU.EXACT("Experiments") OR SU.EXACT("Empirical Methods") OR SU.EXACT("Quantitative Methods")) OR (randomise* OR randomize* OR randomly OR random allocate*) OR (group* AND (random* OR between* OR control* OR intervene*)) OR (interveneion* OR controlled OR control group OR compare OR comparison* OR compared OR prospective* OR crossover OR pretest OR "pre test" OR posttest OR post test OR quasiexperiment* OR quasi experiment* OR evaluate* OR effect* OR effectiveness OR impact OR time series OR time point* OR repeated measure*) OR ((before NEAR/5 after) OR ("pre" NEAR/5 post))) AND ((SU.EXACT("Secondary Education") OR SU.EXACT("Education") OR SU.EXACT("Special Education") OR SU.EXACT("Health Education") OR SU.EXACT("Sex Education") OR SU.EXACT("Courses") OR SU.EXACT("Curriculum") OR SU.EXACT("Teaching Methods") OR SU.EXACT("Teaching") OR SU.EXACT("Computer Assisted Instruction") OR SU.EXACT("Educational Programs") OR SU.EXACT("Literacy Programs") OR SU.EXACT("Educational Plans") OR SU.EXACT("Schools") OR SU.EXACT("High Schools") OR SU.EXACT("Junior High Schools") OR SU.EXACT("Secondary Schools") OR SU.EXACT("Junior High School Students") OR SU.EXACT("High School Students") OR SU.EXACT("Students")) AND (SU.EXACT("Literacy") OR SU.EXACT("Moral Judgment") OR SU.EXACT("Judgment") OR SU.EXACT("Participative Decision Making") OR SU.EXACT("Decision Making") OR SU.EXACT("Problem Solving") OR SU.EXACT("Thinking") OR SU.EXACT("Deduction") OR SU.EXACT("Inference") OR SU.EXACT("Reasoning") OR SU.EXACT("Induction") OR SU.EXACT("Evidence Based Practice")))) AND (CYR(2014* or 2015*) OR DL(2014* or 2015*) OR PD(2014* or 2015*) OR YR(2014* or 2015*)) |

| Social Services Abstracts (ProQuest) – 15.4.2016 |
| --- |
|  |
| (((SU.EXACT("Longitudinal Studies") OR SU.EXACT("Research Design") OR SU.EXACT("Experiments") OR SU.EXACT("Empirical Methods") OR SU.EXACT("Quantitative Methods")) OR (randomise* OR randomize* OR randomly OR random allocate*) OR (group* AND (random* OR between* OR control* OR intervene*)) OR (interveneion* OR controlled OR control group OR compare OR comparison* OR compared OR prospective* OR crossover OR pretest OR "pre test" OR posttest OR post test OR quasiexperiment* OR quasi experiment* OR evaluate* OR effect* OR effectiveness OR impact OR time series OR time point* OR repeated measure*) OR ((before NEAR/5 after) OR ("pre" NEAR/5 post))) AND ((SU.EXACT("Secondary Education") OR SU.EXACT("Education") OR SU.EXACT("Special Education") OR SU.EXACT("Health Education") OR SU.EXACT("Sex Education") OR SU.EXACT("Courses") OR SU.EXACT("Curriculum") OR SU.EXACT("Teaching Methods") OR SU.EXACT("Teaching") OR SU.EXACT("Computer Assisted Instruction") OR SU.EXACT("Educational Programs") OR SU.EXACT("Literacy Programs") OR SU.EXACT("Educational Plans") OR SU.EXACT("Schools") OR SU.EXACT("High Schools") OR SU.EXACT("Junior High Schools") OR SU.EXACT("Secondary Schools") OR SU.EXACT("Junior High School Students") OR SU.EXACT("High School Students") OR SU.EXACT("Students")) AND (SU.EXACT("Literacy") OR SU.EXACT("Moral Judgment") OR SU.EXACT("Judgment") OR SU.EXACT("Participative Decision Making") OR SU.EXACT("Decision Making") OR SU.EXACT("Problem Solving") OR SU.EXACT("Thinking") OR SU.EXACT("Deduction") OR SU.EXACT("Inference") OR SU.EXACT("Reasoning") OR SU.EXACT("Induction") OR SU.EXACT("Evidence Based Practice")))) AND (CYR(2014* or 2015*) OR DL(2014* or 2015*) OR PD(2014* or 2015*) OR YR(2014* or 2015*)) |

| OpenGrey.eu – 15.4.2016 |
| --- |
|  |
| (((health or information or mathematical or quantitative or science or scientific* or media) NEAR/2 (literacy or literate* or illiteracy or illiterate*)) OR (information NEAR/2 competen*) OR numeracy OR ((scientific OR critical) NEAR/2 (skill* or think* or reason*))) |
| Social Care Online – 15.4.2016 |
|  |
| - SubjectTerms:'"children"' including this term only  - OR SubjectTerms:'"school children"' including this term only  - OR SubjectTerms:'"adolescence"' including this term only  - OR SubjectTerms:'"young people"' including this term only  - OR SubjectTerms:'"adolescent boys"'  - OR SubjectTerms:'"adolescent girls"'  - OR SubjectTerms:'"young people"'  AND  - SubjectTerms:'"schools"' including narrower terms  - OR SubjectTerms:'"education"' including this term only  - OR SubjectTerms:'"health education"' including narrower terms  - OR SubjectTerms:'"sex education"' including this term only  - OR SubjectTerms:'"teaching"' including narrower terms  AND  - SubjectTerms:'"literacy"' including this term only  - OR SubjectTerms:'"numeracy"' including this term only  - OR SubjectTerms:'"critical thinking"' including this term only  - OR Title:'literac*'  - OR Abstract:'literac*'  - OR Title: 'critical thinking'  - OR Abstract: 'critical thinking' |

| Social Science Research Network – 15.4.2016 |
| --- |
|  |
| “critical thinking”  “health literacy”  "scientific literacy"  "information literacy"  “media literacy”  “science literacy” |

| Google Scholar – 15.4.2016 |
| --- |
|  |
| **Version 1, first 200 hits screened** |
| ((literacy) or (information competency) or (scientific thinking) or (scientific reasoning) or (critical thinking) or (critical reasoning) or numeracy) AND (education or school or student or teaching or training or course or class or curriculum or instruction or lesson) |
| **Version 2,** **first 200 hits screened** |
| ((health literacy) or (critical literacy) or (scientific literacy) or (critical thinking)) AND (education or school or student or teaching or training or class or curriculum or lesson or instruction) |
| **Version 2,** **first 200 hits screened** |
| ((health literacy) or (critical literacy) or (scientific literacy) or (critical thinking)) AND (education or school or student or teaching or training or class or curriculum or lesson or instruction) AND (health or body or medicine or human) |

| clinicaltrials.gov – 15.4.2016 |
| --- |
|  |
| Advanced search: (Literacy OR literacies OR appraising OR appraisal OR critical thinking) AND Interventional Studies AND Child |

| clinicaltrials.gov – 15.4.2016 |
| --- |
|  |
| Advanced search: (Literacy OR literacies OR appraising OR appraisal OR critical thinking): in title, condition or intervention. Search including all recruitment statuses. |
