# Supplementary material for: Effects of School-Based Educational Interventions for Enhancing Adolescents Abilities in Critical Appraisal of Health Claims: A Systematic Review
Source: PLoS One. 2016 Aug 24;11(8):e0161485. doi: 10.1371/journal.pone.0161485 (PMC4996462; doi:10.1371/journal.pone.0161485)
Supplement: S1 Table — (DOCX) [file pone.0161485.s003.docx]

**S1 Table. Excluded studies**

| Study | **Reason for exclusion** |
| --- | --- |
| Belland 2011 [1] | Human Genome Project used as case, but the intervention targeted ethical reasoning rather than critical appraisal. |
| Brand 2011 [2] | Intervention comprised information seeking for diagnostic and treatment information but did not involve critical appraisal skills, i.e. using knowledge about science methodology to appraise claims and information. |
| Chowning 2012 [3] | Intervention concerned ethical dilemmas and ethical reasoning related to medical cases but did not involve critical appraisal skills, i.e. using knowledge about science methodology to appraise claims and information. |
| Froman 1994 [4] | Irrelevant study design (no control group) and outcomes measured are knowledge of medical science (facts) rather than critical appraisal skills. |
| Gegner 2009 [5] | Intervention aimed at comprehending health science articles, yet not specifically on science methodology and critical appraisal skills. |
| Jacque 2016 [6] | The instrument used to measure claims evaluation and risk assessment seems to be concerned with students’ use of factual knowledge related to health, rather than knowledge about science methodology to appraise claims. |
| Keselman 2007 [7] | Intervention involved critical reasoning and problem solving using scientific content knowledge (facts) about HIV and AIDS, but did not target critical appraisal skills, i.e. using knowledge about science methodology to appraise claims and information. |
| Khisfe 2015 [8] | Irrelevant study design (uncontrolled before-after study). The intervention aimed at increasing students’ knowledge about the nature of science in the context of genetic engineering but with no particular focus on health issues. Before-after study. |
| Khisfe 2013 [9]  Khisfe 2012 [10] | Intervention aimed at increasing students’ knowledge about the nature of science in the context of genetic engineering but with no particular focus on health issues. |
| Larson 2012 [11] | Intervention involved component about critical use of health-related information but did not involve critical appraisal skills, i.e. using knowledge about science methodology to appraise claims and information. |
| Marin 2011 [12] | Intervention targeted critical thinking skills using cases from several topic areas but no specific focus on health or knowledge about science methodology. . |
| Mason 2014 [13] | Intervention involved appraisal of information on controversial science-related topics, many of them health-related, but appraisal does not involve knowledge about science methodology. |
| Mbajiorgu 2003 [14] | Intervention aimed at information evaluation and decision-making in socioscientific issues in biology but no particular focus on health or knowledge about science methodology. |
| Ross 1993 [15] | Intervention comprised knowledge about science methodology (correlational reasoning) in a range of topics, no specific health focus. |
| Steckelberg 2013 [16] | Intervention involved facilitation of health information enhancement, not critical appraisal. Outcome related to girls’ factual knowledge of HPV and risks and not their ability to appraise risk information critically. |
| Tsai 2015 [17] | The intervention include some aspects of health, but the instrument used to measure students’ knowledge about science comprises many items related to non-health contexts (e.g. environmental issues). |
| Tsai 2013 [18] | Intervention involved appraisal of non-health related science news articles in genetics and reproduction. |
| Wilson 2010 [19] | Irrelevant setting: Study laboratory-based summer school over two weeks. |
| Zeidler 2009 [20] | Irrelevant study type. Classes randomised to intervention and control groups, but outcome only measured in some students from each class. |
| Zohar 2002 [21] | Intervention involved using knowledge about human genetics in developing arguments and claims and to take standpoints in ethical dilemmas, but did not target critical appraisal skills, i.e. using knowledge about science methodology to appraise claims and information. |

1. Belland BR, Glazewski KD, Richardson JC. Problem-based learning and argumentation: testing a scaffolding framework to support middle school students' creation of evidence-based arguments. Instr Sci. 2011; 39: 667-94. PMID: EJ935168.
2. Brand LG. Evaluating the effects of medical explorers: a case study curriculum on critical thinking, attitude toward life science, and motivational learning strategies in rural high school students. Doctoral Thesis, Ball State University. 2011.
3. Chowning JT, Griswold JC, Kovarik DN, Collins LJ. Fostering critical thinking, reasoning, and argumentation skills through bioethics education. PLoS One. 2012; 78: e36791. doi: 10.1371/journal.pone.0036791. PMID: 22615814.
4. Froman RD, Owen SV. Can we improve science literacy? Paper presented at the Annual Meeting of the American Educational Research Association; 1994 April 5-8; New Orleans, LA. Available: http://files.eric.ed.gov/fulltext/ED404129.pdf.
5. Gegner J, Mackay D, Mayer R. Computer-supported aids to making sense of scientific articles: cognitive, motivational, and attitudinal effects. ETR&D-Educ Tech Res Dev. 2009; 57: 79-97. doi: 10.1007/s11423-008-9088-3.
6. Jacque B., Koch-Weser S., Faux R., Meiri K. Addressing health literacy challenges with a cutting-edge infectious disease curriculum for the high school biology classroom. Health Educ Behav. 2016; 43: 43-53. doi: http://dx.doi.org/10.1177/1090198115596163
7. Keselman A, Kaufman DR, Kramer S, Patel VL. Fostering conceptual change and critical reasoning about HIV and AIDS. J Res Sci Teach. 2007; 44: 844-63. doiI: 10.1002/tea.20173.
8. Khishfe R. A Look into students' retention of acquired nature of science understandings. Int J Sci Educ. 2015; 37: 1639-1667. doi: 10.1080/09500693.2015.1049241
9. Khishfe R. Transfer of nature of science understandings into similar contexts: promises and possibilities of an explicit reflective approach. Int J Sci Educ. 2013; 35: 2928-53. doi: 10.1080/09500693.2012.672774
10. Khishfe R. Nature of science and decision-making. Int J Sci Educ. 2012; 34: 67-100. doi: 10.1080/09500693.2011.559490.
11. Larson SC. The effects of academic literacy instruction on engagement and conceptual understanding of biology of ninth-grade students. Doctoral Thesis, Aurora University. 2011.
12. Marin LM, Halpern DF. Pedagogy for developing critical thinking in adolescents: explicit instruction produces greatest gains. Think Skills Creat. 2011; 6: 1-13. doi:10.1016/j.tsc.2010.08.002.
13. Mason L, Junyent AA, Tornatora MC. Epistemic evaluation and comprehension of web-source information on controversial science-related topics: effects of a short-term instructional intervention. Comput Educ. 2014; 76:143-57. doi:10.1016/j.compedu.2014.03.016
14. Mbajiorgu NM, Ali A. Relationship between STS approach, scientific literacy, and achievement in biology. Sci Educ. 2003; 87: 31-9. doi: 10.1002/sce.10012.
15. Ross JA, Cousins J. Enhancing secondary school students' acquisition of correlational reasoning skills. Res Sci Techl Educ. 1993; 11: 191-205. doi: http://dx.doi.org/10.1080/0263514930110208.
16. Steckelberg A, Albrecht M, Kezle A, Kasper J, Muhlhauser I. Impact of numerical information on risk knowledge regarding human papillomavirus (HPV) vaccination among schoolgirls: a randomised controlled trial. Ger Med Sci. 2013;1. doi: 10.3205/000183. PMID: 24198750.
17. Tsai C-Y. Improving students' PISA scientific competencies through online argumentation Int J Sci Educ. 2015; 37: 321-339. doi: 10.1080/09500693.2014.987712
18. Tsai P-Y, Chen S, Chang H-P, Chang W-H. Effects of prompting critical reading of science news on seventh graders' cognitive achievement. Int J Environ Sci Educ. 2013; 8: 85-107. Available: http://files.eric.ed.gov/fulltext/EJ1008596.pdf
19. Wilson CD, Taylor JA, Kowalski SM, Carlson J. The relative effects and equity of inquiry-based and commonplace science teaching on students' knowledge, reasoning, and argumentation. J Res Sci Teach. 2010; 47: 276-301. doi: 10.1002/tea.20329.
20. Zeidler DL, Sadler TD, Applebaum S, Callahan BE. Advancing reflective judgment through socioscientific Issues. J Res Sci Teach. 2009; 46: 74-101. doi: 10.1002/tea.20281.
21. Zohar A. Fostering students' knowledge and argumentation skills through dilemmas in human genetics. J Res Sci Teach. 2002; 39: 35-62. doi: http://dx.doi.org/10.1002/tea.10008.
