# Supplementary material for: Effects of School-Based Educational Interventions for Enhancing Adolescents Abilities in Critical Appraisal of Health Claims: A Systematic Review
Source: PLoS One. 2016 Aug 24;11(8):e0161485. doi: 10.1371/journal.pone.0161485 (PMC4996462; doi:10.1371/journal.pone.0161485)
Supplement: S2 Table — (DOCX) [file pone.0161485.s004.docx]

**S2 Table. GRADE Summary of findings**

| **School-based educational intervention for enhancing adolescents’ critical appraisal skills** | | | | | |  |
| --- | --- | --- | --- | --- | --- | --- |
| **Population**: Adolescents in grades 7 to 12 **Setting**: Lower and upper secondary schools in the US and Germany **Intervention**: School-based educational interventions  **Comparison**: Other intervention or instruction as usual | | | | | |  |
| **Educational interventions that compare different teaching modalities** | | | | | |  |
| **Outcomes** | **Comparison of groups** | | **Relative effect**  **(95%)** | **No. of participants**  **(studies)** | **Quality of the evidence**  **(GRADE)** | **Comment** |
|  | Abstracted instruction in causal reasoning | Situated instruction in causal reasoning |  |  |  |  |
| **Knowledge and understanding relevant for critical appraisal** (immediately post-intervention) |  |  |  |  |  |  |
| **Basic knowledge of causality**  Direct testing: Percentage score ≥80 | 77 of 100 | 45 of 100 | RR 1.71  (1.35 to 2.16) | 220 students  (1 RCT) [42] | ⨁◯◯◯ VERY LOW^1,2,4^ |  |
| **Understanding causality**  Direct testing: Given percentage score ≥80, proportion who correctly explained causality | 77 of 100 | 26 of 100 | RR 3.03  (1.83 to 5) | 220 students  (1 RCT) [42] | ⨁◯◯◯ VERY LOW^1,2,4^ |  |
| **Critical appraisal skills: Ability to scientifically evaluate claims** (immediately of shortly following instruction) |  |  |  |  |  |  |
| Self-report. Scale: 0 to 100 points  (100 best) | See comment | See comment | - | 45 students  (1 Non-RCT) [47] | ⨁◯◯◯ VERY LOW^1,2,3,4^ | Statistically significant difference in score in favour of the intervention group (p-value 0.028, means and SDs not provided) |
| Direct testing  Heterogeneous measurement scales | See comment | See comment | - | 239 students (2 Non-RCTs)  [42, 47] | ⨁◯◯◯ VERY LOW^1,3,4^ | Both studies found no difference between groups at posttest (see details in main text). |
| **Behaviour** | Not reported | | - | - | - |  |
| **Educational interventions compared to instruction as usual** | | | | | |  |
| **Outcomes** | **Comparison of groups** | | **Relative effect**  **(95%)** | **No. of participants**  **(studies)** | **Quality of the evidence**  **(GRADE)** | **Comment** |
|  | Instruction as usual | Educational intervention in critical appraisal-related topics |  |  |  |  |
| **Knowledge and understanding relevant for critical appraisal** (shortly following instruction) |  |  |  |  |  |  |
| **Understanding epidemiology**  Self-report. Scale: 5 to 25 points  (25 best) | Mean posttest scores in the control group was  (subgroups only^5^)  17.94 in C_1_ / 17.54 in C_2_ | Mean posttest score was (subgroups only^5^):  3.15* / 3.55* points higher in I_1_  0.74 / 1.14* points higher in I_2_  0.20 / 0.60* points higher in I_3_  (CIs not reported) | - | 998 students  (1 Non-RCT) [43] | ⨁◯◯◯ VERY ^LOW1,2,3^ | Posttest scores adjusted for pretest score, gender, ethnicity, first language, final grades, special education coded and unexcused absence  (* = statistically significant difference, p<.0.5) |
| Direct testing. Scale: 0 to 11 points  (11 best) | Mean posttest scores in the control group was  (subgroups only^5^)  4.17 in C_1_ / 4.18 in C_2_ | Mean posttest score was (subgroups only^5^):  0.71* / 0.70* points higher in I_1_  0.80* / 0.79* points higher in I_2_  0.26 / 0.25 points higher in I_3_  (CIs not reported) |  | 998 students  (1 Non-RCT) [43] | ⨁◯◯◯ VERY ^LOW1,2,3^ |  |
| **Understanding EBM aspects**  Direct skills (test): Total score calculated as person parameters (Rasch model). | Mean posttest person parameters in the control group was 483 (SD 94) | Mean posttest person parameters in the intervention group was 114 parameters higher (85.65 to 142.35 higher). | - | 255 students  (1 Non-RCT) [48] | ⨁◯◯◯ VERY LOW^1,2,3,4^ | An increase in 100 person parameters regarded a relevant difference |
| **Critical appraisal skills: Causal reasoning**  Direct testing immediately post-intervention  Heterogeneous measurements. | See comment | See comment | - | Study 1: 9 classes  Study 2-4: 250 students  (4 Non-RCTs) [40, 44, 45, 46] | ⨁◯◯◯ VERY LOW^1,3,4^ | Statistically significant differences in causal reasoning skills in favour of the intervention group found in all studies (see details in main text). |
| **Behaviour** | Not reported | | - | - | - |  |

^1^Insufficient reliability and validity of outcome measurements.

^2^Only one study

^3^No adjustment for clustering effects and/or possible confounders

^4^Indirectness due to student population (e.g. special education) or education providers (researchers), and/or heterogeneity in measurement scales

^5^**Intervention group 1 (I_1_):** Students of one experienced teacher, 16 lessons (of 34 in total). **Intervention group 2 (I_2_):** Students of four teachers, 16-18 lessons. **Intervention group 3 (I_3_):** Students of one teacher, 6-10 lessons. **Control group 1 (C_1_)**: Students of two teachers who were randomised to control group. **Control group 2 (C_2_)**: Students of twelve teachers who volunteered to take pre- and post-tests.
