# Supplementary material for: Effects of School-Based Educational Interventions for Enhancing Adolescents Abilities in Critical Appraisal of Health Claims: A Systematic Review
Source: PLoS One. 2016 Aug 24;11(8):e0161485. doi: 10.1371/journal.pone.0161485 (PMC4996462; doi:10.1371/journal.pone.0161485)
Supplement: S3 Table — (DOCX) [file pone.0161485.s005.docx]

**S3 Table. Characteristics of included studies** *(ordered by study ID)*

**Derry 1998** [40]

| **Methods** | **Study design:** Non-randomised group study with pre- and post-test  **Unit of allocation:** Teachers  **Setting:** One lower secondary school, Midwest, US | |
| --- | --- | --- |
| **Participants** | **N:** 9 classes (2 intervention group, 7 control group, no. of students not reported)  **Age of learners:** Not reported; grades 8  **Gender:** Not reported  **Ethnicity:** Not specified (“racially and socioeconomically diverse”, p. 177)  **Socioeconomic status:** Not specified  **School performance:** Not reported | |
| **Intervention** | **Description:** Statistical reasoning unit  **Pedagogical framework:** Situated learning/cognition (Lave, 1991; Vygotsky, 1978), Dewey’s (1938) ideas of a school practice that extends adult society, and radical constructivism (e.g. von Glasersfeld, 1990).  **Learning objectives:** To improve students' abilities to think and reason statistically about real-world issues  **Content:**  Day 1-4: Ethics in medical research, experimental control, informed single-case observations versus randomized clinical trials, and governmental regulation of the scientific and lay community.  Day 5-7: Instructions on making presentations; credible scientific research and valid statistical inferences; thinking as evidential argument taught by teachers.  8-15: Student simulation game (roleplay) of a mock legislative hearing dealing with government regulation of the dietary supplement and vitamin industry.  8-11: Students analysed data, developed arguments and designed presentations to support their stakeholder positions (e.g. consumers, health professions, medical researchers, journalists/reporters, state legislators)  12-15: Mock legislative hearing where students presented and defended their arguments.  **Teaching methods:** Small group discussions of popular media presentations that required statistical reasoning, simulation/roleplay of authentic activities in small groups (e.g. conducting research, participating in hearings and conferences) mentored and modelled by teachers, direct instruction combined with class discussions/activities.  **Dosage:** Fifteen lessons of 70 minutes in length over three weeks.  **Provider characteristics:** One teacher for each class respectively (science, social studies), expert scientist (one day), and lead researchers. Teachers’ age, gender and experience not stated. Prior to instruction, teachers and researchers collaborated in planning and in-service training sessions.  **Control group:** Instruction as usual. Teachers of control classes taught a variety of subjects, but most of them likely not science or social studies. | |
| **Outcomes** | **Outcome:** Causal (statistical) reasoning skills  **Methods of assessing outcome:** Test that consisted of a printed dialogue of a fictitious court trial about a health-related case. Two “why” questions followed the dialogue:   - One question that required causal reasoning. Scoring rubric: 2, 1, 0 or -1 points (max score 13, min score -1). Mean score calculated. In addition, proportion of inappropriate responses counted. - One question in which causal reasoning was neither required nor appropriate. Proportion of inappropriate causal explanations counted.   Students could write ≥ 3 statements per question to justify answers.  **Timing of outcome assessment:** Baseline and approximately 10 days post-intervention. | |
| ***Risk of bias*** | | |
| **Bias** | **Authors’ judgement** | **Support for judgement** |
| Sequence generation | High risk | No random sequence generation due to study design: non-randomised controlled study, allocation based on self-selection (volunteers) |
| Allocation concealment | High risk | No allocation concealment, allocation based on self-selection (volunteers). |
| Baseline characteristics & outcome measurements comparable | Unclear risk | Group characteristics at baseline only described in text (p. 177, 189), no data. Although authors adjust for pretest score , no details about differences/similarities between groups are provided. |
| Blinding of students and education providers | Low risk | Blinding of teachers and students not possible. The outcome measures are direct (task-based assessment) and not likely to be influenced by lack of blinding. The post-test was administrated 10 days after the end of intervention (p. 181). Although this might have allowed time for control students to prepare themselves for the test we consider this unlikely. |
| Departures from intended interventions | High risk | Intervention and control teachers worked at the same school. It is possible that communication between teachers could have occurred. The intervention teachers did not teach the control classes. The instruction unit was not implemented as ideally planned. Challenges with regard to student attitudes, discipline and physical arrangements of classrooms were greater in one intervention classroom than the other (p. 191). |
| Blinding of outcome assessment | Unclear risk | Interpretation of students’ answers to test questions required judgment, No information about whether the outcome assessor was blinded. |
| Incomplete outcome data | High risk | Administrative error resulted in an entire comparison class receiving the same task at pre- and post-test. The class was excluded from analysis (p. 189). |
| Selective reporting | High risk | The number of students in each group, the SDs or CIs for mean differences between intervention and control classrooms, and p-values are not reported. |
| Outcome measures reliable | Unclear risk | No information on reliability measures for the test instrument. |
| Outcome measures valid | Unclear risk | No information on validity of the test instrument. One of the tests (John’s Trial) might be less valid because it possibly is conceptually more diverse from the instructional unit contents (p. 189). |
| Other bias? | Unclear risk | No information about teacher characteristics and demographics. Insufficient information about adjustment for confounding factors at student-level. |
| Overall assessment | High risk |  |

**Hill 1998** [42]

| **Methods** | **Study design:** Situated vs abstracted instruction: Randomised controlled study with post-test only.  Transfer instruction: Non-randomised group study with post-test only (Non-RCT)  **Unit of allocation:** RCT: Students. Non-RCT: Class periods  **Setting:** One suburban, public lower secondary school, Charleston, South Carolina, US |
| --- | --- |
| **Participants** | **N:** Situated vs abstracted instruction: 220 (115 situated condition, 105 abstracted condition)  Transfer instruction: Five class periods á 220 students, 194 students completed post-test (situated +transfer/no transfer instruction: 60/41; abstracted +transfer/no transfer instruction: 34/59)  **Age of learners:** Not reported, grades 7  **Gender:** Not reported  **Ethnicity:** 56% African American, 40% Caucasian, 4% Hispanic and Asian  **Socioeconomic status:** 64% of students came from low income and 25% from middle income households  **School performance:** Low average level (more than half the students scored below national average in reading, mathematics and science) |
| **Intervention** | **Description:** Situated instruction or abstracted instruction (comparison) in causal reasoning.  Transfer instruction – applying causal reasoning to real-life situations.  **Pedagogical framework:** Situated learning (Vygotsky, 1978; Lave and Wenger, 1991, and others)  **Learning objectives:** To enable students to identify whether studies encountered in real-life situations are studies of causation or merely association.  **Content:**  Situated instruction: Process of research, differences between causality and correlation, cause-and–effect-factors, nuisance factors, experimental manipulation, random assignment. Examples and practice problems from real-life contexts, i.e. exact copies of magazine articles or pamphlets.  Abstracted instruction: Same contents and examples as situated instruction group, but examples presented in text-form (re-typed).  Transfer instruction: Additional instruction in how to apply the principle of causality to any real-world situation involving claims made in a study e.g. medical studies, advertisement claims, media reports)  **Teaching methods:**  Situated instruction: Teacher as mentor, not lecturer. Teacher modelled how to find important pieces of information to assess causality in real examples. She used coaching, scaffolding and fading to increase independent problem solving. Students compared assessments with those of their teacher (reflection), and applied learning to real examples (exploration). Lesson plan partly scripted to allow modifications to different student knowledge and experience.  Abstracted instruction: Whole class lectures. Lesson plan completely scripted, instruction identical for all students.  Transfer instruction: Whole class instruction on how to apply the principle of causality to various real-world situations. Students worked in small-groups work to apply their own strategies on examples in current magazines. Lesson plan scripted and identical for both the situated and abstracted groups.  **Dosage:**  Situated and abstracted instruction: Four lessons of 50 minutes in length over two weeks  Transfer instruction: One lesson of 50 minutes in length (one week after situated/abstracted instruction).  **Provider characteristics:** Two first-year female science teachers (age not reported). No significant differences in student grades among teachers (no p-value). One teacher reported to be lively, using many hands-on activities in sessions. The other teacher typically used a lecture-based approach. The study author thus assigned the latter teacher to the abstracted instruction (p. 133). The same teachers provided transfer instruction. Both teachers received training two weeks before instruction started.  **Control group:**  Situated vs abstracted instruction: None (comparison of the two approaches).  Transfer instruction: Instruction as usual (no transfer instruction) |

| **Outcomes** | **Outcome:**  Situated vs abstracted instruction: 1) Basic knowledge of causality; 2) Understanding causality.  Transfer instruction: Scientific reasoning: Ability to transfer knowledge and understanding to real-life examples.  **Methods of assessing outcome:**  Situated vs abstracted instruction: Test including three constructed reports of health research in the media. For each scenario students were asked to answer five closed-response questions (e.g. determine whether the researchers manipulated the cause factor and used random assignment to control confounding) and an open-ended question about how they knew a cause-effect relationship was shown. Scores: 0 to 15 points. Basic knowledge of causality: ≥ 12 points, i.e. percentage score ≥ 80). Understanding of causality: percentage score ≥ 80 + correct answer to open-ended question (i.e. explanation of need for experimental manipulation and control of confounding factors).  Transfer instruction: Open-ended test including a study reported by the CNN (“Grapes inhibit cancer growth”). Students were instructed to write a short summary judging the believability of the claim in the story with support for conclusions (“After reading this story, do you think grapes inhibit cancer growth? Why or why not?”). Scoring rubric: 0 to 3. Responses that included reflections about causality as taught in science sessions received a score of 1, 2, or 3, depending on level of accuracy.  **Timing of outcome assessment:**  Situated vs abstracted instruction: Immediately post-intervention.  Transfer instruction: Two weeks post-intervention | |
| --- | --- | --- |
| ***Risk of bias*** | | |
| **Bias** | **Authors’ judgement** | **Support for judgement** |
| Sequence generation  *Situated vs abstracted* | Low risk | Students were randomly assigned to each condition either using a table of random numbers or pulling names out of a hat. |
| Sequence generation  *Transfer* | High risk | Original randomization broken. Allocation of class periods based on researcher’s decision. |
| Allocation concealment  *Situated vs abstracted* | Unclear risk | Unclear whether the study author used an open random allocation schedule (see above). |
| Allocation concealment  *Transfer* | High risk | Allocation of class periods based on researcher’s decision. |
| Baseline characteristics & outcome measurements comparable | Unclear risk | Group characteristics at baseline are not reported, only characteristics for the whole population. No pretest measurement of outcomes. |
| Blinding of students and education providers | Low risk | Blinding of teachers and students not possible. Both groups received an active intervention. Outcomes measured are direct (test) and not likely to be influenced by lack of blinding. Measures were taken to ensure intended delivery of the instructional interventions (see below). |
| Departures from intended interventions | Low risk | Teachers received training beforehand (including transfer instruction). Lesson plans were (partly) scripted and provided to teachers. Instruction in both groups audiotaped to ensure fidelity of implementation. Non-instructional events and interruptions equally distributed across groups. Transfer instruction lesson scripted but not audiotaped. Only one session, thus unlikely that departures from intervention were substantial. |
| Blinding of outcome assessment | Low risk | Study author reports that scorers were blinded |
| Incomplete outcome data | Low risk | According to the author 216 students consented to participate (Hill, p. 133). However, there are 220 students accounted for in the analysis (Hill, p. 142). This number is also reported in the article (Hendricks, 2001). We assume 220 students consented to participate and completed the post-test given that the author specifically report the number of students lost-to-follow-up with regard to the transfer instruction.  Transfer instruction: 194 (11%) lost-to-follow-up. Distribution across groups similar: 12/105 in AI-group, 14/115 in SI-group (Hill, p. 149). |
| Selective reporting | Low risk | No study protocol available, but no reason to suspect selective reporting. |
| Outcome measures reliable? | High risk | Knowledge and understanding of causality: The reliability indices are too low (<0.85) for reliable measures at the individual level and, possibly, for groups of respondents. One would anticipate artificial high reliability indices (here: 0.72-0.81) as only changing the scenarios across units would result in dependent items and consequently redundancy in the data.  Transferring knowledge to real-life contexts: The sample size for measuring test-rest reliability is too small (18 students only). |
| Outcome measures valid? | High risk | We consider the instruments as not being reliable and hence not valid. |
| Other bias?  *Situated vs abstracted* | Unclear risk | Teachers assigned to conditions according to their preferred instructional approaches: One teacher was more comfortable using lectures, and thus assigned the abstracted condition. |
| Other bias?  *Transfer* | High risk | The author did not correct for matching, clustering effects or other confounding factors. |
| Overall assessment  *Situated vs abstracted* | Moderate risk |  |
| Overall assessment  *Transfer instruction* | High risk |  |

**Kaelin 2007** [43]

| **Methods** | **Study design:** Non-randomised group study with pre- and post-test  **Unit of allocation:** Teachers  **Setting:** 16 lower secondary school, New Jersey, US | |
| --- | --- | --- |
| **Participants** | **N:** 16 schools, 998 students (6 schools á 378 students in intervention group, 10 schools á 620 students in control group)  **Age of learners:** 12.3 years (12.2 intervention group, 12.4 control group), grades 7  **Gender:** 47% males (45% intervention group, 48% control group)  **Ethnicity:** 54% Hispanic, 37% African American, 7% Caucasian, 2% Asian (evenly distributed across groups)  **Socioeconomic status:** Low, 88% received free or reduced lunch (91% intervention group, 87% control group)  **School performance:** Diverse, mean grade close to C (evenly distributed across groups) | |
| **Intervention** | **Description:** Epidemiology curriculum (Detectives in the Classroom)  **Pedagogical framework:** Understanding by Design: Building effective curricula based on essential questions than can be answered by enduring understandings, i.e. big disciplinary ideas of lasting value outside school (Wiggins & McTighe, 1998).  **Learning objectives:** “..to help students “uncover” the epidemiologic principles and apply what they learn to health issues of interest in their personal and public lives” (p. 17).  **Content:** Five modules, each focusing on one of five epidemiologic essential questions (e.g. “Is s there an association between the hypothesized cause and the disease?”) and its enduring understanding. Content topics:   1. Using descriptive epidemiological information to generate hypotheses about distribution of a disease in a group of people. 2. Using analytic epidemiology and study designs to investigate associations; testing hypothesis by observing the exposures and diseases in people. 3. Evaluation of causality, considering flaws in observational studies and identifying other explanations. 4. The important, yet also limited, role of epidemiological science in societal decisions about risk and prevention. Competing values, social, economic, and political factors. 5. Assessing prevention strategy effectiveness by comparing the risks of disease in populations of people who were and were not exposed to the strategy. Costs, trade-offs, and alternative strategies.   (p. 17 and Table 1). Curriculum can be accessed from: [www.montclair.edu/Detectives/curriculum/](http://www.montclair.edu/Detectives/curriculum/).  **Teaching methods:** Module investigations in small groups (Epi Teams). Individual completion of Investigation worksheets. Worksheets kept in a portfolio (Epi Logs). Concept maps, quizzes, and assessment (pre-test/post-tests).  **Dosage:** Total curriculum: 34 lessons of 1-2 periods in length. Teachers free to decide number of lessons per module. In study: 6 to 18 lessons taught. Intervention group divided into subgroups depending on teachers’ experience with curriculum and lessons taught: Intervention_1_ (one more experienced teacher, 16 lessons), Intervention_2_ (four teachers, 16 to 18 lessons), and Intervention_3_ (one teacher, 6-10 lessons).  **Provider characteristics:** Two male and six female science teachers. Teachers’ age and years of experience not available. Intervention teachers attended preparation workshops prior to each module, which included demonstrations lessons and discussions of implementation in classrooms, revisions, and experiences teaching the previous module.  **Control group:** Science instruction as usual. | |
| **Outcomes** | **Outcome:** Epidemiological knowledge and understanding  **Methods of assessing outcome:** Multidimensional questionnaire of 62 items, grouped into five constructs. Two constructs relevant to this review:   - Enduring understanding of epidemiology (self-report, five items, 5-point Likert scale of “Definitely do not understand” to “Definitely understand”, min score 5, max score 25) - Knowledge of epidemiology (direct measure, 11 multiple-choice questions, max score 11)   **Timing of outcome assessment:** Baseline (Nov/Dec 2002), post-test (late May – mid-June). Nov/Dec), “My best estimate of average time between pre- and post-tests is 5-6 months” (personal communication with author) | |
| **Risk of bias** | | |
| **Bias** | **Authors’ judgement** | **Support for judgement** |
| Sequence generation | High risk | Nine teachers volunteered to participate in the study and were randomised to experimental group (n=7) and control group (n=2). Teachers who volunteered their students to take pre-test/post-test (n=12) constituted a second control group (self selection). |
| Allocation concealment | High risk | No random sequence generation due to study design: Nine teachers randomly assigned to intervention and control groups. Twelve teachers volunteered as control group only (self-selected). |
| Baseline characteristics & outcome measurements comparable | Unclear risk | The author adjust pot-test scores for gender, nationality, first language, final grades in health, mathematics, and science, special education code, unexcused absences, and pre-test score. There are no details about pre-test scores at baseline. |
| Blinding of students and education providers | Low risk | Blinding of teachers and students not possible. Outcomes unlikely to be influenced by lack of blinding because intervention and control groups were located at different schools and informed consent from students was waived (i.e. students unaware they participated in a study). |
| Departures from intended interventions | High risk | The number of lessons taught varied considerably between teachers (from 6 to 18 lessons, p. 25) and none of the teachers taught the suggested 30+ lessons. No measures taken to validate instruction delivery (e.g. video- or audiotaping). |
| Blinding of outcome assessment | Low risk | The two outcomes were assessed using a Likert-scale (self-reported knowledge) and multiple-choice test. It is not likely that unblinded outcome assessors make a difference. |
| Incomplete outcome data | High risk | Substantial attrition and unevenly distributed between groups: 34% in intervention group, and 27% in control group (17%). These students may be different from students who participated. |
| Selective reporting | Low risk | No study protocol available, but no reason to suspect selective reporting. |
| Outcome measures reliable | Unclear risk | The internal consistency (Cronbach’s alpha) coefficients ranged from 0.72 to 0.82. The sample size is too small (< 50 students) for stable item estimates based on the current data. Reliability indices reported are too low for reliable measures at the individual level but might work for groups of respondents. |
| Outcome measures valid | Unclear risk | Insufficient data on validity. |
| Other bias? | High risk | Teacher characteristics and demographics (other than gender) not factored into analysis. Statistical analysis does not correct for clustering effects. |
| Overall assessment | High risk |  |

**Kuhn 2015 – Study A and B** [44, 45]

| **Methods** | **Study design:** Non-randomised group study with post-test only  **Unit of allocation:**  A**:** Schools.  B: Classes in one school.  **Setting:**  A: One public middle school in Harlem, New York and one independent middle school in New York City, US.  B: The public middle school described in study A. | |
| --- | --- | --- |
| **Participants** | **N:**  A: 106 students (62 in intervention group, 44 in control group).  B: 89 students in 3 classes (58 students (2 classes) in I, 31 students (1 class) in C)  **Age of learners:** Not reported. Grades 8 in study A. Grades 7 in study B.  **Gender:** Groups reported to be equally divided by gender, no data.  **Ethnicity:** Not specified at group level.  A: Intervention school population predominantly Hispanic and African-American, the lowest proportion Caucasian and Asian. No information about control group.  B: Same population as intervention group in study A.  **Socioeconomic status:**  A: Intervention school population came from socioeconomically disadvantaged backgrounds, 60% qualified for free or reduced-price lunch. Control school high SES.  B: Same population as intervention group in study A.  **School performance:**  A: Intervention and control groups came from academically disadvantaged and advantaged backgrounds respectively.  B: Not reported. School population came from an academically disadvantaged background. | |
| **Intervention** | **Description:** Multivariable causal reasoning unit  **Pedagogical framework:** None specified.  **Learning objectives:** Promote the development of scientific thinking skills through recognising that multiple variables may interact and contribute to an outcome.  **Content:**  A: Two topics, obesity in childhood and teen crime. *Obesity:* Students reflected on possible factors contributing to obesity (hypothesis generation) and collected data on weight, height, and lifestyle (food intake and exercise) for three adults of their own choice. They entered data into a database that allowed multiple ways of representing data graphically to facilitate analysis. Following analysis, students prepared a report of results. The intervention ended with a final individual assignment where students wrote an essay about childhood obesity. *Teen crime:* Same arrangement as obesity topic, but students analysed pre-collected data (63 cases drawn from authentic sources) already registered in the database.  B: Less extended intervention, *obesity* topic only.  **Teaching methods:** Whole-class discussions, data collection and analysis, report writing, blackboard logs, individual assignments.  **Dosage:**  A: 24 lessons over 6 weeks, i.e. four lessons per week. Duration of lessons not reported.  B: 9 lessons over 3 weeks.  **Provider characteristics:**  A: Two of the lead researchers and an assistant taught the unit to the intervention classes, in consultation with their science teacher. The control group was taught by their regular science teacher (female).  B: Students’ science teacher delivered the instruction, assisted by the 2nd author.  **Control group:**  A: Instruction as usual. The science teacher reported that scientific inquiry activities were a significant part of the curriculum.  B: Instruction as usual – genetics unit. Instruction delivered by the same science teacher as in the intervention group. | |
| **Outcomes** (same in A and B) | **Outcome:** Causal reasoning: 1) Understanding that multiple variables influence an outcome; 2) Understanding the need for comparisons to make inferences about causation  **Methods of assessing outcome:** Open response test that consisted of a public health scenario about the difference in prevalence of cancer between an urban and suburban area in Ohio. Students asked to describe a study to identify potential causes. Students’ responses coded as to whether they recognised zero, one, or two or more (multivariable) variables as potential causes, and whether they recognised the need to make a comparison of the groups (areas).  **Timing of outcome assessment:** Immediately post-intervention. | |
| ***Risk of bias*** | | |
| **Bias** | **Authors’ judgement** | **Support for judgement** |
| Sequence generation | High risk | A: No random sequence generation due to study design: non-randomised controlled study, allocation based on self-selection (volunteers)  B: Only three classes randomly assigned to intervention and control conditions. Randomisation cannot balance out any characteristics of the classes that might affect the outcomes. |
| Allocation concealment | High risk | A: No allocation concealment, allocation based on self-selection (volunteers).  B: No true randomisation, see above. |
| Baseline characteristics & outcome measurements comparable | High risk | A: Students in the intervention group came from socioeconomically and academically disadvantaged backgrounds, while the opposite was the case in the control group. Although justified by author as “doing so worked against our hypothesis of an effect of the intervention, thus providing a more rigorous test of it” (p. 98), it still presents high risk of bias as the groups were not similar at baseline. The intervention group had previously been exposed to a scientific inquiry unit (Life Expectancy) targeting the learning aims in the curriculum tested. No pretest measurements of outcomes.  B: The authors state that there was “ample evidence of the initial equivalence” between the three classes (p. 105) but there is no data to support this. No pretest measurements of outcomes. |
| Blinding of students and education providers | Low risk | A and B: Blinding of teachers and students not possible. The outcome measures are direct (task-based assessment) and not likely to be influenced by lack of blinding. Moreover, the outcomes were measured immediately after educational intervention. |
| Departures from intended interventions | A: Unclear risk  B: High risk | A: There is no information about co-interventions or whether the units were delivered as intended. The risk of bias due to contamination is low because the control group attended another schools.  B: There is no information about co-interventions or whether the units were delivered as intended, i.e. no information about whether lessons were observed, audiotaped etc., or what kind of training the teacher received ahead on implementing the unit in his classes. Contamination is possible because the same teacher taught both the intervention and comparison classes. |
| Blinding of outcome assessment | Unclear risk | A and B: Interpretation of students’ answers to test questions required judgment, No information about whether the outcome assessor was blinded. |
| Incomplete outcome data | High risk | A and B: The number of subjects reported were only those who completed the post-test. Dropouts were excluded from analysis (personal communication with author) |
| Selective reporting | Low risk | A and B: No study protocol available, but no reason to suspect selective reporting. |
| Outcome measures reliable | Unclear risk | A and B: The authors describe the instrument used and how they score participants’ responses, but there is no information on reliability of the test instrument. |
| Outcome measures valid | Unclear risk | A and B: The authors describe the instrument used and how they score participants’ responses, but there is no information on validity of the test instrument. |
| Other bias? | High risk | A and B: Insufficient information about adjustment for confounding factors and clustering effects at student-level. The authors probably present unadjusted estimates only. |
| Overall assessment | A and B:  High risk |  |

**Leshowitz 1993** [46]

| **Methods** | **Study design:** Non-randomised group study with post-test only  **Unit of allocation:** Teachers  **Setting:** One lower and one upper secondary school in a suburban area of Arizona, US | |
| --- | --- | --- |
| **Participants** | **N:** 55 students (22 special education students in intervention group, 33 general education students in control group)  **Age of learners:** Range 16-20, grades 7-12  **Gender:** Not reported (67% males intervention group, control group not reported)  **Ethnicity:** 68% Caucasian and 32% Hispanic in intervention group, ethnicity in control group reported as similar.  **Socioeconomic status:** Low middle class in intervention group, SES in control group reported as similar.  **School performance:** Grade equivalents ranged from 2^nd^ to 10^th^ grade in intervention group, not reported for control group | |
| **Intervention** | **Description:** Instructional unit in critical thinking that emphasised statistical-methodological reasoning.  **Pedagogical framework:** Not reported.  **Learning objectives:** “..to facilitate the development of critical thinking skills through the application of rules of scientific reasoning to analysis of information and events encountered in everyday life.” (p. 484). Specific objectives included learning the following elements of scientific reasoning: defining independent and dependent variables, depicting relationships between variables in X-Y plots, and analysing experimental design, including the need for control groups and distinguishing between random assignment to groups and self-selected groups.  **Content:** The first part of the unit was devoted to the evaluation of facts and opinions in authentic advertisements. Students identified the claim, labelled it as either fact or opinion, and determined whether the information provided was relevant to the claim made. In the second part of the unit, students evaluated various topical and scientific studies described in brief newspaper and magazine articles. They defined the bottom-line conclusion, determined whether the data obtained in the reported study supported the conclusion, and summarised the evidence by drawing graphs that related the independent to the dependent variable. Additionally, students determined the causal relationship between variables by applying rules of causality, i.e. “does the independent variable precede the dependent variable in time?” and “can the effect be attributed to the independent variable? Alternatively, is the influence of extraneous ‘third’ variables or ‘rival hypotheses’ controlled or removed in the study by the use of a control group?” (p. 486).  **Teaching methods:** The teachers used a Socratic dialogue approach (teacher-guided discussions) rather than a traditional lecture format “to foster a learning environment of critical inquiry” (p. 484).  **Dosage:** Twenty-five lessons of 45 minutes in length every school day over four to six weeks.  **Provider characteristics:** Two female special education teacher students in their early 30’s and 40’s respectively. Both students delivered the intervention as part of their student teaching practicum. They had been student teaching for a few months at the time of the study, but did not have any prior full-time teaching experience.  **Control group:** Science instruction as usual. | |
| **Outcomes** | **Outcome:** Causal reasoning  **Methods of assessing outcome:** Short-answer open-response test consisting of two parts, including an advertisement from magazine or newspaper and an article that summarised scientific data. Student answers were judged on the following three categories (subtests): (1) Identification of the claim; (2) Graph of the actual or hypothetical data; (3) Explanation of whether the data constituted proof of the claim. Each category was scored on a 2-point scale (0-2 points). Total score: 6 points.  **Timing of outcome assessment:** Immediately post-intervention | |
| **Risk of bias** | | |
| **Bias** | **Authors’ judgement** | **Support for judgement** |
| Sequence generation | High risk | No random sequence generation due to study design: non-randomised controlled study, allocation based on self-selection (volunteers) |
| Allocation concealment | High risk | No allocation concealment, allocation based on self-selection (volunteers). |
| Baseline characteristics & outcome measurements comparable | High risk | Intervention: Special education students; Control: General education students. Although justified by study author “…to effect a more stringent test of the effectiveness of the instructional intervention than (…) had a comparable group of special education students served as a control group” we consider risk of bias to be high due to differences in learning abilities between groups. |
| Blinding of students and education providers | Unclear risk | Blinding of teachers and students not possible. The teachers who provided the instruction were also participating researchers (study authors, p. 484). This may have influenced how they acted towards students (e.g. did they try harder than other teachers would have done?) There is no information about whether the lessons were scripted and/or observed to detect this possible performance bias. |
| Departures from intended interventions | Unclear risk | Information about co-intervention or fidelity of implementation not provided. The control class attended the same high school as some of the intervention group students and teachers, but contamination unlikely because intervention teachers were part of research team and intervention students were attending special education classes. |
| Blinding of outcome assessment | High risk | Interpretation of students’ answers to test questions required judgment. Tests were scored independently by the two participating teachers. They were not blinded because they assisted some students in reading the test. |
| Incomplete outcome data | Unclear risk | No attrition in intervention group. We do not know if any students in the control class were absent because only students who were present the day of testing were included. |
| Selective reporting | Low risk | No study protocol available, but no reason to suspect selective reporting. |
| Outcome measures reliable | High risk | Too few items. Although interrater reliability is high (0.89) It does not follow that the outcome measure is reliable. The authors claim that items tap into “independent dimensions” (interpreted as multidimensional trait and not uncorrelated traits as actually stated). The reported reliability index (Cronbach’s alpha = 0.51) is only a valid measure of reliability given unidimensional data, and the authors claim that their data consist of “independent dimensions”. |
| Outcome measures valid | High risk | We consider the instruments as not being reliable (see above) and hence not valid. The “graph test”, assessing ability to interchange between different forms of representations (represent data in tables as graphs), might be interpreted as being part of the cognitive category “reasoning” but does it measure “critical thinking skills” specifically? |
| Other bias? | High risk | Student teachers in intervention group had little full-time teaching experience. Teacher characteristics and demographics not collected and factored into analysis. Statistical analysis does not correct for clustering effects or confounding factors. |
| Overall assessment of risk of bias | High risk |  |

**Powell 2014** [47]

| **Methods** | **Study design:** Non-randomised group study with pre- and post-test  **Unit of allocation:** Classes  **Setting:** One suburban high school, Tampa Bay, Florida, US | |
| --- | --- | --- |
| **Participants** | **N:** 2 classes á 45 students (1 class á 25 students in intervention group, 1 class á 20 students in control group)  **Age of learners:** Not reported, grades 9  **Gender:** Not reported  **Ethnicity:** 61% Caucasian, 25% Hispanic, 10% African American, 5% Asian/others  **Socioeconomic status:** Various backgrounds. School data: More than 50% economically disadvantaged  **School performance:** Biology Honors students. Students’ 8^th^ grade Science and English scores similar in both groups. | |
| **Intervention** | **Description:** Integrated biology curriculum comprising three units in socioscientific issues (SSI). The first unit, “Evaluation of Evidence”, was relevant to the systematic review.  **Pedagogical framework:** SSI instruction, i.e. using real-world situations with a scientific component to increase relevance of science. Integrating political, economic and ethical issues of science to engage students in discourse with peers and teachers, negotiate different points of view and conducting research to enhance understanding of science in society (Zeidler & Nichols, 2009; Zeidler et al., 2011)  **Learning objectives:** To enable students to evaluate evidence, make informed decisions on SSI and integrate scientific content knowledge when reasoning about SSI.  **Content:** The evidence unit included three sub-units. (1) Extrasensory perception: Sensational scientific claims, evaluating information and data to determine scientific evidence. (2) Thinking inside the box: Making indirect observations and reporting on those observations, conducting peer review of research proposals. (3) Cell phone use and cancer: The basic elements of scientific studies, evaluating studies, analysing news headlines in relation to their content and determine their accuracy based on information from scientific studies.  **Teaching methods:** Teacher as mentor rather than lecturer. Individual and group presentations and assignments, debates and discussions, lectures and laboratory activities to reinforce students’ learning of different concepts, computers and iPads, clicker activities. Magazine headlines, articles, advertisements and YouTube videos used to introduce topics.  **Dosage:** Twelve lessons of 55 minutes in length over five weeks.  **Provider characteristics:** One science teacher taught both groups (classes). She had four years of teaching experience, held a Bachelor’s degree in Animal science, and was certified to teach biology in grades 6-12. The teacher was chosen “because she was always looking for new ways to enhance her pedagogical strategies with her students” (p. 66). The principal investigator provided initial and ongoing training on SSI throughout the study, and helped leading some discussions in the intervention class. He had been a teacher at the school for over nine years and had five years’ experience in using SSI instruction.  **Control group:** Sub-units 1 and 2 in the evidence unit (see descriptions of content above) were integrated into a traditional biology curriculum introducing topics (properties of life, cells, genetics) as organised in students’ textbook. | |
| **Outcomes** | **Outcome:** Ability to evaluate claims using scientific evidence  **Methods of assessing outcome:** *Direct skills:* Open-response test that contained a fictitious news brief describing the conclusion and results from a scientific study about stem cell therapy. Students were asked to generate questions they wanted answered to determine the conclusion’s validity. Their responses were scored using a coding scheme adapted from Korpan (1994). Maximum total score was 34 points. *Self-report:* Students were also asked to rate their ability to evaluate evidence on a scale ranging from 0-100.  **Timing of outcome assessment:** Immediately post-intervention, i.e. after the end of the unit | |
| **Risk of bias** | | |
| **Bias** | **Authors’ judgement** | **Support for judgement** |
| Sequence generation | High risk | Only two classes randomly assigned to intervention and control conditions. Randomisation cannot balance out any characteristics of the classes that might affect the outcomes. |
| Allocation concealment | High risk | No true randomisation, see above. |
| Baseline characteristics & outcome measurements comparable | Unclear | *Baseline characteristics:* Author state that students in both groups had various background experiences and compared the two groups with regard to academic scores (Science, English, Algebra). However, no data provided to allow comparison of groups (p. 80). No information about gender distribution across groups/classes. *Outcome measurements:* No information about pre-test results in the two classes. |
| Blinding of students and education providers | O1: High risk  O2: Low risk | Blinding of teachers and students not possible.  *Outcome 1 - Evaluation of evidence (self-assessment):* High risk of bias. Lack of blinding might have influenced students’ self-assessment of their ability to evaluate evidence and they knew they were participating in a study. *Outcome – Outcome 2 - Evaluation of evidence (direct skills):* Low risk of bias. Both groups received an active intervention. Outcome (direct skills) not likely to be influenced by lack of blinding. |
| Departures from intended interventions | Unclear risk | The skills tested are advanced, “contamination” of knowledge and skills unlikely to have occurred. The researcher observed some sessions in the intervention group to ensure fidelity. He also observed sessions in the control class, the frequency of observations is however unclear. No video- or audiorecording of all sessions in both groups. It is also unclear whether the teacher started implementation of SSI-instruction in the intervention class before the start of the study, which increases the risk of co-intervention. |
| Blinding of outcome assessment | O1: Low risk  O2: Low risk | *Outcome 1 - Evaluation of evidence (self-assessment):* Low risk of bias. Since students are the outcome assessors, the risk of bias is high for this domain. However, this is already considered under “Blinding of students and education providers”.  *Outcome – Outcome 2 - Evaluation of evidence (direct skills):* Low risk of bias. Both groups received an active intervention. Outcome (direct skills) not likely to be influenced by lack of blinding. Potential student identifiers were removed before the data were presented to analysts for review. |
| Incomplete outcome data | Low risk | All the students from both classes started and completed the investigation (personal communication with author). |
| Selective reporting | O1: High risk  O2: High risk | *Outcome 1- Evaluation of evidence (self-assessment):* No means and SDs for each of the groups, only p-value.  *Outcome 2 - Evaluation of evidence (direct skills):* No means and SDs for each of the groups, no p-values. |
| Outcome measures reliable | Unclear risk | The authors describe the instrument used and how they score participants’ responses, but no data on assessment of reliability is provided. In scoring students’ responses the author reports that disagreements occurred that were resolved by consensus. However, there are no data on inter-rater reliability. |
| Outcome measures valid | Unclear risk | The authors describe the instrument used and how they score participants’ responses, but no data on assessment of validity is provided. |
| Other bias? | High risk | There is insufficient information about whether the author adjusted for confounders or clustering effects. The authors probably present unadjusted estimates only. |
| Overall assessment of risk of bias | High risk |  |

**Steckelberg 2009** [48]

| **Methods** | **Study design:** Non-randomised group study with post-test only  **Unit of allocation:** Classes  **Setting:** Twelve classes in upper secondary schools (Fachgymnasium, Gesamtschule, Gymnasium) in Hamburg, Germany | |
| --- | --- | --- |
| **Participants** | **N:** 255 students (37 intervention group, 218 control group)  **Age of learners:** Range 16-18 (mean age 17.6 intervention group, 17.4 control group), grades 7-12  **Gender:** 38% males (44% intervention group, 36% control group)  **Ethnicity:** 18% non-ethnically German (24% intervention group, 17% control group).  **Socioeconomic status:** Not reported.  **School performance:** Not reported | |
| **Intervention** | **Description:** ebm@school curriculum.  **Pedagogical framework:** Klafki’s five questions. The questions “promote systematic reflection regarding aims and intentions of instruction as a prerequisite for the development process of the curriculum” (p. 159).  **Learning objectives:** Overall objective was “to enhance critical health literacy, which implies the recognition of the benefit of independent acquisition and critical appraisal of information” (p. 160-1). In addition, the authors define 33 specific learning objectives relating to the six module topics (see below), for example differentiate between expert based and evidence based information, understand RRR, ARR and NNT, define precision and accuracy of diagnostic tests, know criteria for analysing systematic reviews (p. 160).  **Content:**   - Module 1 - Fallacies and misinterpretations of data representations: differences between observational studies and RCTs: Expert-based versus evidence-based information; consequences of misleading health information; fallacies of medical/health issues; study designs for investigating effectiveness; methodological and statistical terms; RRR vs ARR. - Module 2 - Critical appraisal of RCTs: Scientific articles and vocabulary; epidemiological statistics (e.g. incidence, prevalence, CIs, p-values); surrogate versus patient-relevant outcomes; framing data; NNT; question formulation; ethics in clinical research. - Module 3 - Informed choice in diagnostic tests: Test results; quality criteria (e.g. sensitivity); precision and accuracy of tests; question formulation; benefit/harm, ethical aspects of screening; framing of diagnostic data. - Module 4 - Understanding systematic reviews: Aims and methods of SRs; access; critical analysis - Module 5- Searching the Internet and databases: Internet and MedPilot; Boolean operators; search techniques (e.g. thesaurus); question formulation. - Module 6 - Appraising patient information: EBM information; critical appraisal; sources; primary vs secondary literature; access to secondary literature; benefits/limits of quality codes   **Teaching methods:** Wide range, including workbook with worksheets for the six modules, lectures with discussion, brainstorming, class and small group discussion, flip charts, posters, overhead transparencies, project work over several days, Metaplan (card technique system for collecting ideas during group work) and computer projections.  **Dosage:** Twenty-two lessons + 10-12 project work lessons over one week (could also be taught over a longer period). Duration of lessons not reported.  **Provider characteristics:** Delivered by researchers. Teachers of intervention classes only offered to be present.  **Control group:** Instruction as usual. | |
| **Outcomes** | **Outcome:** Critical health competency, i.e. knowledge and skills in the areas of evidence-based medicine as specified under the interventions contents (see above)  **Methods of assessing outcome:** Critical Health Competency Test (Steckelberg et al., 2009): 72 multiple-choice and short-answer open-response items embedded in four different scenarios (echinea and common cold, MRI in knee injuries, acne treatment, breast cancer screening). Measures four subareas of competences: Understanding medical concepts, searching skills, basic statistics, design of experiments. Total score calculated as person parameters (Rasch model).  **Timing of outcome assessment:** Immediately post-intervention | |
| **Risk of bias** | | |
| **Bias** | **Authors’ judgement** | **Support for judgement** |
| Sequence generation | High risk | No random sequence generation due to study design: non-randomised controlled study, allocation based on researchers’ decisions. |
| Allocation concealment | High risk | No allocation concealment, allocation based on researchers’ decisions. |
| Baseline characteristics & outcome measurements comparable | High risk | The control group comprised Gymnasium students and may therefore include more high-achieving students than the intervention group. No pretest measurement of outcomes. |
| Blinding of students and education providers | Unclear risk | Blinding of teachers and students not possible. Low motivation may have influenced test scores for intervention students in the second pilot because there were no more lessons before summer holidays for the parallel classes. |
| Departures from intended interventions | High risk | Although researchers undertook instruction, class teachers were offered to be present. Some of these teachers might also have taught control classes, making contamination possible. The curriculum changed from first to second pilot, which limit the interpretation of results. |
| Blinding of outcome assessment | Unclear risk | Interpretation of students’ answers to some of the test questions required judgment, and there was no information about whether outcome assessor was blinded. |
| Incomplete outcome data | Unclear risk | Eight students (18%) lost-to-follow-up in intervention group. We do not know if any students in control classes were absent because only student who were present the day of testing were included. |
| Selective reporting | Low risk | No study protocol available, but no reason to suspect selective reporting. |
| Outcome measures reliable | High risk | The instrument (Critical Health Competence Test) was still under development when study was conducted. According to the author the Rasch scalability was not yet achieved (Steckelberg et al., 2009). |
| Outcome measures valid | High risk | We consider the instruments as not being reliable (see above) and hence not valid. |
| Other bias? | High risk | Teacher characteristics and demographics not collected and factored into analysis. Control for other confounding factors probably not done. Statistical analysis does not correct for clustering effects. |
| Overall assessment of risk of bias | High risk |  |

**References**

Dewey J. (1938). *Experience and education*. New York: Collier.

Lave J. (1991). Situating learning in communities of practice. In: Resnick L, Levine J, Tealey S, editors. *Perspectives on socially shared cognition*. Washington, DC: APA Press, p. 63-82.

Steckelberg A, Hulfenhaus C, Kasper J, Rost J, Muhlhauser I. (2009). How to measure critical health competences: development and validation of the Critical Health Competence Test (CHC Test). *Advances in Health Sciences Education : Theory and Practice*, 14: 11-22.

von Glasersfield E. (1990). An exposition of constructivism: why some like it radical. In: Davis RB, Maher CA, Noddings N, editors. *Monographs of the Journal for Research in Mathematics Education, #4*. Reston, VA: National Council of Teachers of Mathematics, p. 9–29.

Vygotsky LS. (1978) *Mind in society: the development of higher-psychological processes* (M. Cole, V. John-Steiner, S. Scribner, & E. Souberman, Eds. and Trans). Cambridge, MA: Harvard University Press.

Wiggins G, McTighe J. (1998). *Understanding by design*. Alexandria, VA: Association for Supervision and Curriculum Development.

Zeidler D, Nichols B. (2009). Socioscientific issues: theory and practice. *Journal of Elementary Science Education*, 21: 49-58

Zeidler D, Applebaum S, Sadler T. (2011). Enacting a socioscientific issues classroom: Transformative transformations. In: Sadler T, editor. *Socio-scientific issues in science classrooms: Teaching, learning and research*. The Netherlands: Springer, p. 277-306.
